# A New Human-Based Metaheuristic Algorithm for Solving Optimization Problems Based on Technical and Vocational Education and Training

**DOI:** 10.3390/biomimetics8060508

**Published:** 2023-10-23

**Authors:** Marie Hubalovska, Stepan Major

**Affiliations:** Department of Technics, Faculty of Education, University of Hradec Kralove, CZ50003 Hradec Kralove, Czech Republic; stepan.major@uhk.cz

**Keywords:** optimization, human-based, metaheuristic, education, technical and vocational education and training, exploration, exploitation

## Abstract

In this paper, a new human-based metaheuristic algorithm called Technical and Vocational Education and Training-Based Optimizer (TVETBO) is introduced to solve optimization problems. The fundamental inspiration for TVETBO is taken from the process of teaching work-related skills to applicants in technical and vocational education and training schools. The theory of TVETBO is expressed and mathematically modeled in three phases: (i) theory education, (ii) practical education, and (iii) individual skills development. The performance of TVETBO when solving optimization problems is evaluated on the CEC 2017 test suite for problem dimensions equal to 10, 30, 50, and 100. The optimization results show that TVETBO, with its high abilities to explore, exploit, and create a balance between exploration and exploitation during the search process, is able to provide effective solutions for the benchmark functions. The results obtained from TVETBO are compared with the performances of twelve well-known metaheuristic algorithms. A comparison of the simulation results and statistical analysis shows that the proposed TVETBO approach provides better results in most of the benchmark functions and provides a superior performance in competition with competitor algorithms. Furthermore, in order to measure the effectiveness of the proposed approach in dealing with real-world applications, TVETBO is implemented on twenty-two constrained optimization problems from the CEC 2011 test suite. The simulation results show that TVETBO provides an effective and superior performance when solving constrained optimization problems of real-world applications compared to competitor algorithms.

## 1. Introduction

An optimization problem is a type of problem that has more than one feasible solution. The process of finding the most feasible solution among all available solutions for the problem is called optimization [1]. An optimization problem can be mathematically modeled using three parts: decision variables, constraints, and objective functions. The goal of optimization is to determine the values of the decision variables, respecting the constraints of the problem, in order to optimize (minimize/maximize) the objective function [2]. There are numerous optimization problems in many science, engineering, industry, and real-world applications that must be solved using appropriate optimization techniques. In optimization studies, problem solving techniques are classified into two groups: deterministic and stochastic approaches [3]. Deterministic approaches in gradient-based and non-gradient-based classes can successfully solve convex, linear, continuous, differentiable, and low-dimensional problems [4]. But, in dealing with complex and especially high-dimensional problems, they lose their efficiency by getting stuck in inappropriate local solutions [5]. Meanwhile, many optimization problems in science and real-world applications are non-convex, non-linear, discontinuous, non-differentiable, complex, and high-dimensional. The difficulties and disadvantages of deterministic approaches have led researchers to deal with these types of optimization problems by introducing stochastic approaches [6].

Metaheuristic algorithms are among the most widely used stochastic approaches that are able to provide suitable solutions for optimization problems based on random searches in the problem-solving space. Easy implementation, simple concepts, and efficiency in non-convex, non-linear, discontinuous, non-differentiable, complex, and high-dimensional problems are among the advantages of metaheuristic algorithms that have led to the popularity of these approaches among researchers. The optimization process in metaheuristic algorithms starts with the random generation of a number of candidate solutions. Then, based on the steps of updating the algorithm, these candidate solutions are improved in an iteration-based process. At the end, the best candidate solution is introduced as a solution to the problem [7]. The nature of the random search means that there is no guarantee of achieving the global optimum using metaheuristic algorithms. However, because the solutions obtained from metaheuristic algorithms are close to the global optimum, they are acceptable as quasi-optimal solutions. The desire of researchers to achieve more effective solutions for optimization problems has led to the design of countless metaheuristic algorithms [8].

Metaheuristic algorithms can perform the search process effectively when they scan the search space well at both global and local levels. Global search, with the concept of exploration, indicates the effectiveness of the metaheuristic algorithm in the accurate scanning of different areas of the search space with the aim of avoiding a stop in the process at local optima. Local search, with the concept of exploitation, indicates the efficiency of the metaheuristic algorithm in converging towards better solutions based on careful scanning near the discovered solutions and promising areas in the problem-solving space. In addition to having high exploration and exploitation abilities, the ability to balance exploration and exploitation is the main key to the success of metaheuristic algorithms in achieving more effective solutions for optimization problems [9].

The main research question is that, according to the metaheuristic algorithms that have been developed so far, is there still a need to design newer algorithms or not? In response to this question, the No Free Lunch (NFL) [10] theorem explains that the successful performance of a metaheuristic algorithm in solving a set of optimization problems is not a guarantee for the similar performance of that algorithm in solving other optimization problems. This means that there is no default result of implementing a metaheuristic algorithm on an optimization problem. According to the NFL theorem, there is no metaheuristic algorithm that performs best in solving all optimization problems. Therefore, the NFL theorem, by keeping open the studies of metaheuristic algorithms, encourages researchers to achieve more effective solutions for optimization problems by designing newer algorithms.

The novelty and innovation of this paper is in introducing a new human-based metaheuristic algorithm called Technical and Vocational Education and Training-Based Optimizer (TVETBO), which is used to handle optimization tasks. The proposed TVETBO approach can be used to solve all kinds of optimization problems in science, such as constrained, unconstrained, and real-world applications. The main contributions of this study are as follows:

- TVETBO is designed based on imitating the skill-training process in technical and vocational education and training schools.
- The fundamental inspiration of TVETBO is derived from the impacts of technical and vocational education and training in preparing young people for work based on training and improving work-related skills.
- The implementation steps of TVETBO are described and mathematically modeled in three phases of (i) theory education, (ii) practical education, and (iii) individual skills development.
- The efficiency of TVETBO in solving optimization problems has been challenged with the CEC 2017 test suite.
- The performance of TVETBO is compared against twelve well-known metaheuristic algorithms.
- TVETBO’s ability to handle real-world applications is evaluated on twenty-two constrained optimization problems from the CEC 2011 test suite.

The structure of the paper is as follows: A literature review is presented in Section 2. Then, the proposed Technical and Vocational Education and Training-Based Optimizer (TVETBO) is introduced and modeled in Section 3. Simulation studies and results are presented in Section 4. The effectiveness of TVETBO in solving real-world applications is investigated in Section 5. Conclusions and suggestions for future research are provided in Section 6.

## 2. Literature Review

Metaheuristic algorithms have been developed with inspiration from various natural phenomena, animal behavior in nature, biological, genetics, and physics sciences, game rules, human behavior, and other evolutionary phenomena. Based on the main design idea, metaheuristic algorithms are classified into five groups: swarm-based, evolutionary-based, physics-based, human-based, and game-based approaches.

Swarm-based metaheuristic algorithms are designed based on the modeling of natural behaviors and strategies of animals, aquatic animals, insects, and other living organisms in the wild. Particle Swarm Optimization (PSO) is one of the most widely used swarm-based metaheuristic algorithms, which was inspired by the collective movement of birds and fish that are searching for food [11]. Ant Colony Optimization (ACO) was inspired by the ability of ants to discover the optimal route between the nest and food sources [12]. Artificial Bee Colony (ABC) was inspired by the cooperation between honeybees in a colony to obtain food resources [13]. The Firefly Algorithm (FA) was inspired by the optical communication between fireflies [14]. The strategies of searching for and providing food resources through foraging and hunting, migration, and digging the ground are among the most common behaviors and strategies of wildlife that have been sources of inspiration when designing algorithms such as the Coati Optimization Algorithm (COA) [15], Grey Wolf Optimizer (GWO) [16], White Shark Optimizer (WSO) [17], Orca Predation Algorithm (OPA) [18], Walruses Optimization Algorithm (WOA) [19], Marine Predator Algorithm (MPA) [20], Red Panda Optimization Algorithm (RPOA) [21], African Vultures Optimization Algorithm (AVOA) [22], Honey Badger Algorithm (HBA) [23], Golden Jackal Optimization (GJO) [24], Subtraction-Average-Based Optimizer (SABO) [25], Whale Optimization Algorithm (WOA) [26], Pelican Optimization Algorithm (POA) [27], Tunicate Swarm Algorithm (TSA) [28], and Reptile Search Algorithm (RSA) [29].

Evolutionary-based metaheuristic algorithms are designed based on the modeling concepts of biology, genetics, survival of the fittest, natural selection, and evolutionary operators. The Genetic Algorithm (GA) [30] and Differential Evolution (DE) [31] are the most famous evolutionary-based metaheuristic algorithms whose designs are inspired by the generation process, Darwin’s theory of evolution, genetic concepts, natural selection, and evolutionary operators of random crossover, mutation, and selection. The body’s defense mechanism against microbes and diseases inspired the design of Artificial Immune Systems (AISs) [32]. Some other evolutionary-based metaheuristic algorithms are the Cultural Algorithm (CA) [33], Genetic Programming (GP) [34], and the Evolution Strategy (ES) [35].

Physics-based metaheuristic algorithms are designed based on the modeling of physics phenomena, forces, transformations, laws, and concepts. Simulated Annealing (SA) [36] is one of the most widely used physics-based metaheuristic algorithms, whose design was inspired by the phenomenon of metal annealing. Cosmological phenomena and concepts are employed in the design of algorithms such as the Black Hole Algorithm (BHA) [37] and the Multi-Verse Optimizer (MVO) [38]. Physical forces and Newton’s laws of motion have been sources of inspiration when designing algorithms such as the Momentum Search Algorithm (MSA) [39], Spring Search Algorithm (SSA) [40], and Gravitational Search Algorithm (GSA) [41]. Some other physics-based metaheuristic algorithms are the Lichtenberg Algorithm (LA) [42], Water Cycle Algorithm (WCA) [43], Equilibrium Optimizer (EO) [44], Nuclear Reaction Optimization (NRO) [45], Electro-Magnetism Optimization (EMO) [46], Thermal Exchange Optimization (TEO) [47], Henry Gas Optimization (HGO) [48], and the Archimedes Optimization Algorithm (AOA) [49].

Human-based metaheuristic algorithms are designed based on modeling communication, interactions, thoughts, decisions, and strategies of humans in their social and individual lives. Teaching-Learning Based Optimization (TLBO) is one of the most widely used human-based metaheuristic algorithms, whose design is inspired by the classroom environment and interactions between teachers and students [50]. The Mother Optimization Algorithm (MOA) is based on Eshrat’s care of her children in three phases: education, advice, and upbringing [51]. Doctor and Patient Optimization (DPO) is introduced by simulating therapeutic interactions and communication between patients and doctors [52]. Subsequently, the Optimization Algorithm (FOA) was proposed with the inspiration of community people following the leader in order to progress in that community [53]. The process of voting and holding elections is employed in the design of the Election-Based Optimization Algorithm (EBOA) [54]. The effort and practice of students in learning sewing from instructors in sewing schools has been a source of inspiration in the design of Sewing Training-Based Optimization (STBO) [55]. Some other human-based metaheuristic algorithms are War Strategy Optimization (WSO) [56], Ali Baba and the Forty Thieves (AFT) [57], the Teamwork Optimization Algorithm (TOA) [58], the Chef-Based Optimization Algorithm (CHBO) [59], the Drawer Algorithm (DA) [60], Driving Training-Based Optimization (DTBO) [5], the Gaining Sharing Knowledge-Based Algorithm (GSK) [61], and the Coronavirus Herd Immunity Optimizer (CHIO) [62].

Game-based metaheuristic algorithms are designed based on modeling the rules of games, and the behaviors of players, referees, coaches, and other persons who influence the games. The mathematical modeling of competitions and the efforts of clubs in different leagues has been a source of inspiration in algorithms such as Football Game-Based Optimization (FGBO) [63] and Volleyball Premier League (VPL) [64]. The skill and competition of players in throwing darts towards the scoreboard in the game of darts are the main ideas behind the design of Darts Game Optimizer (DGO) [65]. The competition among players when changing their movements based on the direction of the referee’s hand and running in different areas of the playing field is employed in the design of the Orientation Search Algorithm (OSA) [66]. Some other game-based metaheuristic algorithms are the Puzzle Optimization Algorithm (POA) [67], Dice Game Optimizer (DGO) [68], Ring Toss Game-Based Optimization (RTGBO) [69], the Golf Optimization Algorithm (GOA) [70], the Archery Algorithm (AA) [6], and the Hide Object Game Optimizer (HOGO) [71].

Based on the best knowledge obtained from the literature review, no metaheuristic algorithm has so far been inspired by the process of teaching skills to applicants in technical and vocational education and training schools. Meanwhile, educational interactions between applicants and instructors are intelligent processes that have special potential for designing a new optimizer. Therefore, in order to address this research gap in the studies of metaheuristic algorithms, in this paper, a new metaheuristic algorithm called Technical and Vocational Education and Training-Based Optimizer (TVETBO) is introduced based on the modeling of the educational space of technical and vocational education and training schools, which is discussed in the next section. 

The special advantage of TVETBO is that there are no control parameters in its design, and therefore, it does not need the parameter setting process. Another advantage of TVETBO is the allocation of separate phases in order to manage exploration and exploitation during the search process in the problem-solving space. TVETBO also has advantages such as easy concepts, a simple mathematical model, and easy implementation in optimization problems. The ability of TVETBO to handle optimization tasks in different sciences and real-world applications is another advantage of this proposed approach. However, TVETBO also has limitations and disadvantages. The first disadvantage of TVETBO is that, similar to all metaheuristic algorithms, since it is a type of stochastic approach, there is no guarantee that the global optimum will be achieved using TVETBO. Another disadvantage of TVETBO is that, based on the NFL theorem, it cannot be claimed that TVETBO is the best optimizer for all optimization applications. Another disadvantage of TVETBO is that there is always a possibility that newer metaheuristic algorithms may be designed that perform better compared to TVETBO.

## 3. Technical and Vocational Education and Training-Based Optimizer

In this section, the inspiration source and theory of the proposed Technical and Vocational Education and Training (TVETBO) approach are stated. Then, its implementation steps are mathematically modeled to show how it can be used to solve optimization problems. The proposed TVETBO approach can be used for all kinds of constrained and unconstrained optimization problems in science and real-world applications. In this paper, the original single-objective version of TVETBO is presented, which has applications in the optimization of single-objective optimization problems. In order to evaluate the performance of TVETBO in solving single-objective optimization problems, twenty-nine optimization problems from the CEC 2017 test suite and twenty-two real-world constrained optimization problems from the CEC 2011 test suite are considered in this paper.

### 3.1. Inspiration for TVETBO

Technical and vocational education and training refers to all forms and levels of training and education which provide skills and knowledge related to occupations in various sectors of economic and social life through formal and informal learning methods in both school-based and work-based learning contexts [72]. Technical and vocational education and training serves multiple goals. The main goal is the preparation of youth for work. This takes the form of learning and developing work-related skills and the mastery of underlying knowledge and scientific principles. Work is broadly defined and therefore refers to both formal employment and self-employment. With the aim of supporting self-employment, technical and vocational education and training curricula often include entrepreneurship education [73]. Applicants are trained by referring to technical and vocational education and training schools, and instructors try to teach the job topics and skills theoretically and practically.

Technical and vocational education and training has various details and scenarios, including the years of study and possible degrees achieved by students. The most important feature that should be considered in the design of metaheuristic algorithms is that the algorithm should be understandable and have simple equations so that readers and researchers can easily understand and use it. Therefore, by using simplified assumptions and ignoring the details in the process of technical and vocational education and training, an attempt was made to design a simple reader-friendly metaheuristic algorithm. Among the educational interactions between applicants and instructors in technical and vocational education and training schools, the activities of (i) theory education, (ii) practical education, and (iii) individual skills development are much more significant. The mathematical modeling of these intelligent activities is employed in the design of the proposed TVETBO approach, which is discussed below.

### 3.2. Algorithm Initialization

The proposed TVETBO approach is a population-based metaheuristic algorithm whose members are applicants who refer to technical and vocational education and training in order to learn skills. Each TVETBO member specifies values for decision variables based on their position in the search space. Therefore, each TVETBO member is a candidate solution for the problem and can be mathematically modeled using a vector so that each element of this vector represents a decision variable. TVETBO members together form the population of the algorithm, which can be modeled from a mathematical point of view using a matrix according to Equation (1). The initial positions of the applicants in the search space are randomly initialized using Equation (2).

(1) $$A={\left[\begin{array}{c} A_{1}\\ \vdots \\ A_{i}\\ \vdots \\ A_{N} \end{array}\right]}_{N\times m}={\left[\begin{array}{ccccc} a_{1,1} & \cdots & a_{1,d} & \cdots & a_{1,m}\\ \vdots & \ddots & \vdots & ⋰ & \vdots \\ a_{i,1} & \cdots & a_{i,d} & \cdots & a_{i,m}\\ \vdots & ⋰ & \vdots & \ddots & \vdots \\ a_{N,1} & \cdots & a_{N,d} & \cdots & a_{N,m} \end{array}\right]}_{N\times m}$$

(2) $$a_{i,d}=lb_{d}+r\cdot \left(ub_{d}-lb_{d}\right)$$

Here, X is the TVETBO population matrix, $A_{i}$ is the ith applicant (candidate solution), $a_{i,d}$ is its dth dimension in the search space (decision variable), N is the number of population members, m is the number of decision variables, r is a random number in an interval [0,1], and $lb_{d}$, and $ub_{d}$ are the lower bound and upper bound of the dth decision variable, respectively.

The objective function of the problem corresponding to the proposed values of each TVETBO member for the decision variables can be evaluated. The set of evaluated values for the objective function can be represented using a vector corresponding to Equation (3).

(3) $$F={\left[\begin{array}{c} F_{1}\\ \vdots \\ F_{i}\\ \vdots \\ F_{N} \end{array}\right]}_{N\times 1}={\left[\begin{array}{c} F\left(A_{1}\right)\\ \vdots \\ F\left(A_{i}\right)\\ \vdots \\ F\left(A_{N}\right) \end{array}\right]}_{N\times 1}$$

Here, F is the vector of evaluated objective function, and $F_{i}$ is the evaluated objective function based on the ith applicant.

The evaluated values for the objective function of the problem are the suitable criteria to compare the quality of the population members in providing candidate solutions. In such a way, the best evaluated value for the objective function corresponds to the best member and the worst evaluated value for the objective function corresponds to the worst member. Considering that, in each iteration of TVETBO, the positions of applicants in the search space are updated, the values of the objective function are also updated. As a result, based on the comparison of the new values obtained for the objective function, the best member should be updated in each iteration.

### 3.3. Mathematical Modeling of TVETBO

The proposed TVETBO approach is an iteration-based algorithm. In its design, the positions of population members are updated in three phases based on a simulation of the process of technical and vocational education and training. In the following text, the process of updating the applicants’ positions in the search space is presented.

#### 3.3.1. Phase 1: Theory Education (Exploration)

In technical and vocational education and training schools, instructors try to introduce skills to applicants in theory. In the first phase of TVETBO, the positions of the population members are updated based on the process of the instructor teaching the skills theory to the applicants. As training of the applicants by the instructor can increasingly improve the skills and knowledge of the applicants, this process has been used to improve the positions of TVETBO members in the problem-solving space. This strategy leads to extensive changes in the positions of population members and, as a result, increases the exploration power of the algorithm for global searches in the problem-solving space. Therefore, in the design of TVETBO, based on the simulation of matching the knowledge levels of the applicants to the knowledge level of the instructor, the positions of the members of the algorithm population change in the problem-solving space. In the design of TVETBO, the best member is considered to be the trainer. Based on the training interactions between the trainer and the applicants, for each of the applicants, a new position is calculated using Equation (4). Then, if the value of the objective function is improved, this new position replaces the previous position of the corresponding member according to Equation (5).

(4) $$a_{i,d}^{P1}=a_{i,d}+r\cdot \left(I_{d}-S\cdot a_{i,d}\right),\;i=1,2,\;\ldots ,\;N,\;\mathrm{and}\;d=1,2,\;\ldots ,m$$

(5) $$A_{i}=\{\begin{array}{cc} A_{i}^{P1}, & F_{i}^{P1}<F_{i}\\ A_{i}, & else \end{array}$$

Here, $A_{i}^{P1}$ is the new suggested position of the *i*th applicant (i.e., a TVETBO member as a candidate solution for the given problem) based on the simulation of matching the knowledge level of the applicant to the instructor, $a_{i,d}^{P1}$ is its dth dimension which refers to the new value for the *d*th decision variable proposed by the *i*th TVETBO member, $F_{i}^{P1}$ is its objective function value which is calculated based on placing the values of the decision variables proposed by $A_{i}^{P1}$ in the objective function of the problem, r is a random number with a normal distribution in the range of [0,1] that is used to create a random nature in TVETBO’s performance, I is the instructor, $I_{d}$ is its dth dimension, S is a random number from the set {1,2}, which expresses the speed of action of the applicants when learning from the instructor, N is the number of applicants, and m is the number of decision variables.

#### 3.3.2. Phase 2: Practical Education (Exploration)

After the theory training, the trainer tries to teach technical and vocational skills to the applicants in a scientific way in training workshops. In the second phase of TVETBO, the simulation of this process is used to update the population. Just as a beginner applicant under the training of an instructor can turn into a professional over time, this process is employed to improve the positions of TVETBO members in the problem-solving space to discover better solutions. Modeling this process leads to extensive changes in the positions of population members and, as a result, increases the discovery power of the algorithm in order to manage the global search in the problem-solving space. In the design of TVETBO, based on the simulation of the applicants’ efforts in sampling the applicants’ skills and learning all of the skills during the training course, the positions of the population members become closer and closer to the assumed position of the instructor during the iterations of the algorithm. Based on the modeling of the imitation of the instructor by applicants when learning technical and vocational skills, a new position for each applicant is calculated using Equations (6) and (7). Then, if the value of the objective function is improved, this new position replaces the previous position of the corresponding member according to Equation (8).

(6) $$K\left(t\right)=r\;\cdot \frac{t}{T}$$

(7) $$a_{i,d}^{P2}=I_{d}+K\left(t\right)\left(a_{i,d}-I_{d}\right),\;\;\;\;i=1,2,\;\ldots ,\;N,\;\;\;\;d=1,2,\;\ldots ,m,\;\;\;\mathrm{and}\;t=1,2,\;\ldots ,\;T$$

(8) $$A_{i}=\{\begin{array}{cc} A_{i}^{P2}, & F_{i}^{P2}<F_{i}\\ A_{i}, & else \end{array}$$

Here, $A_{i}^{P2}$ is the new suggested position of the ith applicant (i.e., a TVETBO member as a candidate solution for the given problem) based on simulating the applicant’s imitation when learning practical skills from the instructor, $a_{i,d}^{P2}$ is its dth dimension, which refers to a new value for the *d*th decision variable proposed by the *i*th TVETBO member, $F_{i}^{P2}$ is its objective function value, which is calculated based on placing the values of the decision variables proposed by $A_{i}^{P2}$ in the objective function of the problem, K(t) represents the practical education imitation coefficient that is acquired within the period of education, where a bigger K(t) (its maximum value is 1) indicates an increase in the practical skills of the applicant, t is the iteration counter of the algorithm, and T is the maximum number of algorithm iterations. 

#### 3.3.3. Phase 3: Improving Individual Skills (Exploitation)

After undergoing theoretical and scientific studies in technical and vocational education and training schools, applicants try to improve their skills in order to perform their jobs better. In the third phase of TVETBO, based on the simulation of individual skill improvement, the algorithm population is updated. As the individual efforts and exercises of applicants can have small positive effects by improving their skills, this process is used to create small positive changes for TVETBO members in the problem-solving environment. Modeling this process leads to small changes in the positions of the population members and, as a result, increases the exploitation power of the algorithm for local searches in the problem-solving space. Therefore, in the design of TVETBO, based on the simulation of individual skill improvements of applicants over time, the positions of the population members in the algorithm change and improve. At the same time as the skills are improved and the applicants become more professional, the range of positive changes becomes smaller over time. Based on this fact, in the design of TVETBO, the range of positive changes during the iterations of the algorithm becomes smaller so that the algorithm can manage the local search with more precise steps. Therefore, based on the modeling of the applicants’ effort to improve their performance, for each applicant, a new position is calculated using Equation (9). Then, if the value of the objective function is improved, this new position replaces the previous position of the corresponding member according to Equation (10).

(9) $$a_{i,d}^{P3}=a_{i,d}+\left(1-2r\right)\cdot \frac{\left(ub_{d}-lb_{d}\right)}{t},\;i=1,2,\;\ldots ,\;N,\;\;\;\;d=1,2,\;\ldots ,m,\;\mathrm{and}\;t=1,2,\;\ldots ,\;T$$

(10) $$A_{i}=\{\begin{array}{cc} A_{i}^{P3}, & F_{i}^{P3}<F_{i}\\ A_{i}, & else \end{array}$$

Here, $A_{i}^{P3}$ is the new suggested position of the ith applicant (i.e., TVETBO member as the candidate solution for a given problem) based on simulating the individual skill improvements of applicants over time, $a_{i,d}^{P3}$ is its dth dimension, which refers to a new value for the *d*th decision variable proposed by the *i*th TVETBO member, $F_{i}^{P3}$ is its objective function value, which is calculated based on placing the values of the decision variables proposed by $A_{i}^{P3}$ in the objective function of the problem, t is the iteration counter of the algorithm, and T is the maximum number of algorithm iterations. $lb_{d}$ and $ub_{d}$ are the lower bound and upper bound of the dth decision variable, respectively. The values of the upper and lower bounds are among the constraints of the optimization problem, and this information is available in the description of the mathematical model of the given problem.

### 3.4. Repetition Process, Pseudocode, and Flowchart of TVETBO

After updating the positions of all TVETBO members in the search space based on Phase 1 to Phase 3, the first TVETBO iteration is completed. Then, the algorithm enters the next iteration with new updated values, and the process of updating the TVETBO members’ positions in the search space continues using Equations (4)–(10) until the last iteration of the algorithm. In each iteration, the best candidate solution obtained until that iteration is updated and stored. After the completion of TVETBO’s implementation, the best candidate solution obtained during the iterations of the algorithm is presented as a solution to the problem. The pseudocode of TVETBO’s implementation steps is presented in Algorithm 1.
**Algorithm 1:** Pseudocode of TVETBO.**Start TVETBO.**1.Input problem information: variables, objective function, and constraints.2.Set the TVETBO population size (*N*) and iterations (*T*).3.Generate the initial population matrix at random using Equation (2). $a_{i,d}\leftarrow lb_{d}+r\cdot \left(ub_{d}-lb_{d}\right)$4.Evaluate the objective function.5.For t=1 to *T*6.For i=1 to N
7.Phase 1: theory education (exploration)8.Update the best population member as the instructor.9.Calculate the new position of the *i*th TVETBO member using Equation (4). $a_{i,d}^{P1}=a_{i,d}+r\cdot \left(I_{d}-S\cdot a_{i,d}\right)$
10.Update the *i*th TVETBO member using Equation (5). $A_{i}\leftarrow \{\begin{array}{cc} A_{i}^{P1}, & F_{i}^{P1}<F_{i}\\ A_{i}, & else \end{array}$
11.Phase 2: practical education (exploration)12.Calculate the imitation coefficient of the instructor’s skill using Equation (6). $K\left(t\right)\leftarrow r\;\cdot \frac{t}{T}$
13.Calculate the new position of the *i*th TVETBO member using Equation (7). $a_{i,d}^{P2}\leftarrow I_{d}+K\left(t\right)\left(a_{i,d}-I_{d}\right)$
14.Update the *i*th TVETBO member using Equation (8). $A_{i}=\{\begin{array}{cc} A_{i}^{P2}, & F_{i}^{P2}<F_{i}\\ A_{i}, & else \end{array}$
15.Phase 3: individual skills development (exploitation)16.Calculate the new position of the *i*th TVETBO member using Equation (9). $a_{i,d}^{P3}=a_{i,d}+\left(1-2r\right)\cdot \frac{\left(ub_{d}-lb_{d}\right)}{t}$
17.Update the *i*th TVETBO member using Equation (10). $A_{i}=\{\begin{array}{cc} A_{i}^{P3}, & F_{i}^{P3}<F_{i}\\ A_{i}, & else \end{array}$
18.End19.Save the best candidate solution so far.20.End 21.Output the best quasi-optimal solution obtained with the TVETBO.End TVETBO.

### 3.5. Computational Complexity of TVETBO

In this subsection, the computational complexity of the proposed TVETBO approach is studied. The preparation and initialization of TVETBO has a complexity equal to *O(Nm)*, where *N* is the number of population members and *m* is the number of problem variables. In the TVETBO design, the positions of members are updated in each iteration in three phases. Therefore, the computational complexity of the process of updating the positions of TVETBO members in the search space is equal to *O(3NmT)*, where *T* is the maximum number of iterations of the algorithm. According to this, the total computational complexity of the proposed TVETBO approach is equal to O(Nm(1+3T)).

## 4. Simulation Studies and Results

In this section, the performance evaluation of the proposed TVETBO approach in terms of solving optimization problems is discussed. For this purpose, TVETBO was used to handle the CEC 2017 test suite for different dimensions of 10, 30, 50, and 100. 

### 4.1. Performance Comparison and Experimental Settings

The performance of TVETBO was compared with the performance of twelve well-known metaheuristic algorithms consisting of GA [30], PSO [11], GSA [41], TLBO [50], MVO [38], GWO [16], WOA [26], MPA [20], TSA [28], RSA [29], AVOA [22], and WSO [17]. The values of control parameters of metaheuristic algorithms are specified in Table 1. In handling the CEC 2017 test suite, the TVETBO approach and each of the competitor algorithms were implemented in 51 independent runs, where each independent run included 10,000·m (m is the number of variables) FEs and a population size of 30. The optimization results are reported using six statistical indicators: the mean, best, worst, standard deviation (std), median, and rank. In order to rank the metaheuristic algorithms in the optimization of each of the benchmark functions, a comparison of the mean index values is used.

### 4.2. Evaluation of the CEC 2017 Test Suite

**Figure 1:**
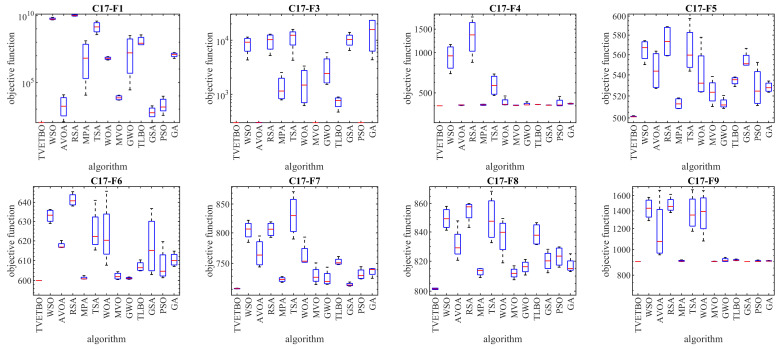

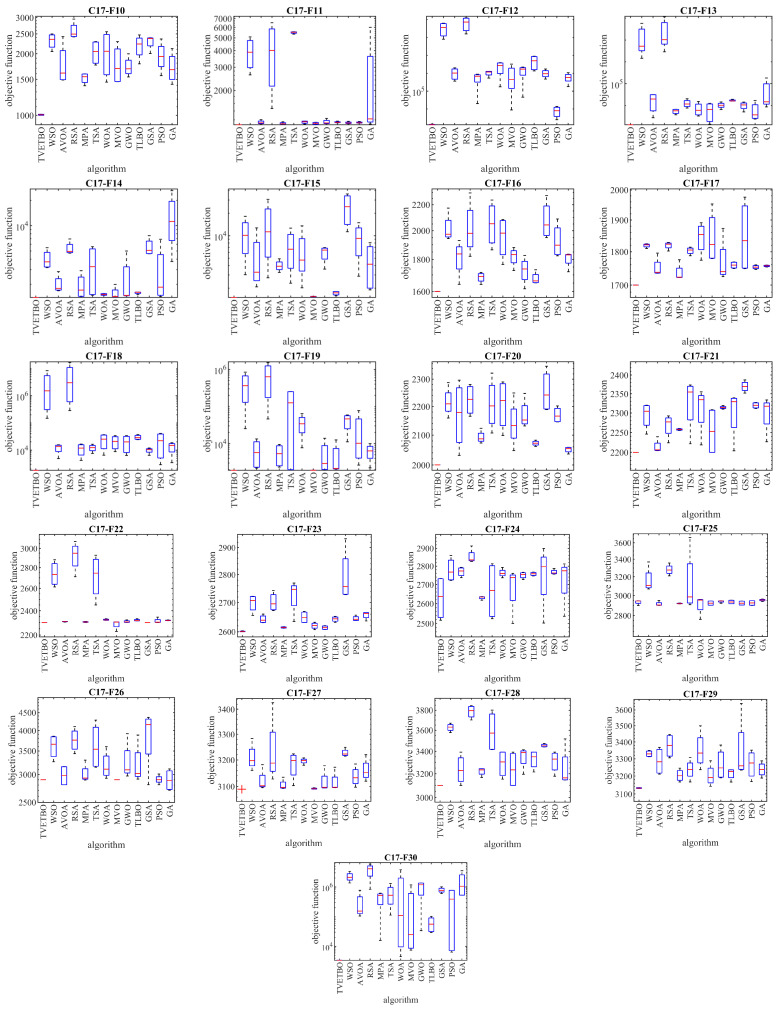
Boxplot diagrams of TVETBO and competitor algorithms’ performances on the CEC 2017 test suite (dimensions = 10).

**Figure 2:**
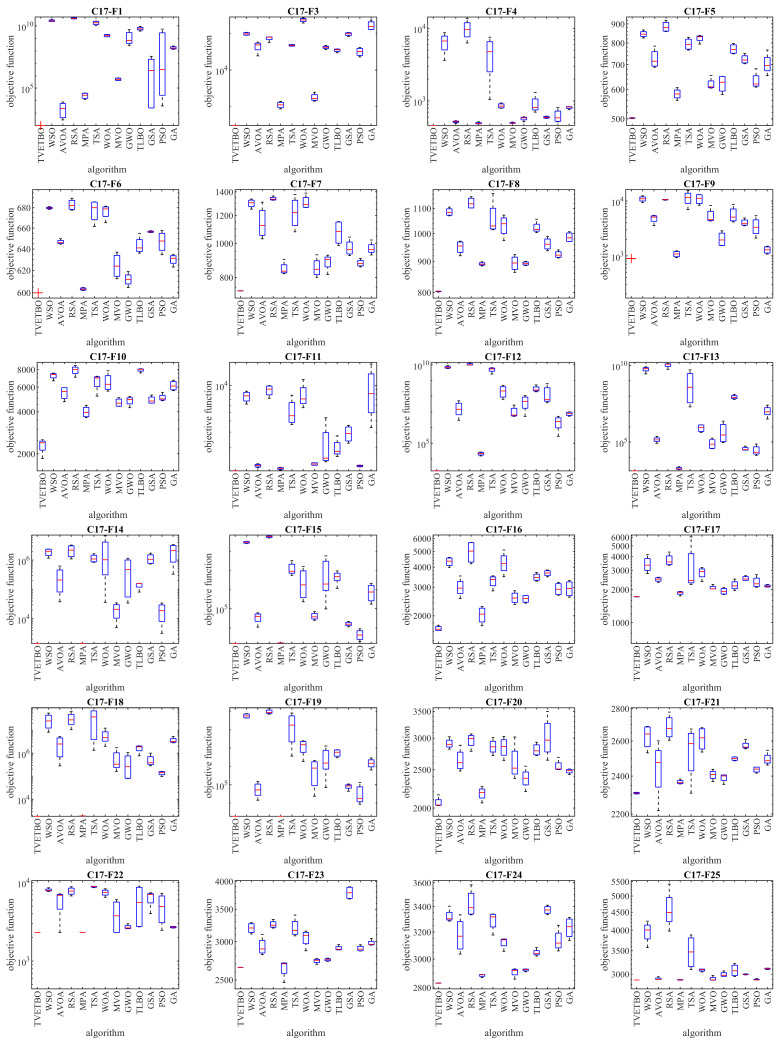

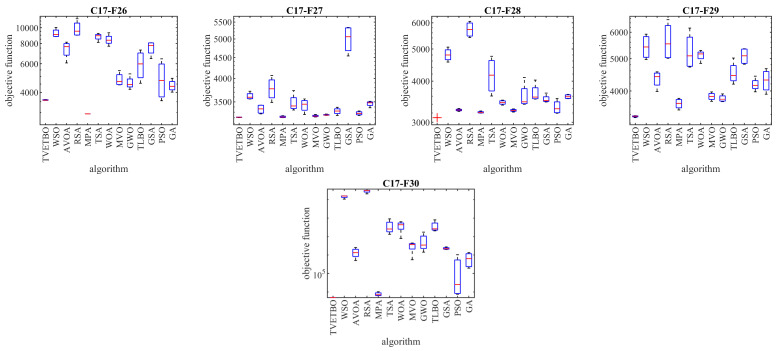
Boxplot diagrams of TVETBO and competitor algorithms’ performances on the CEC 2017 test suite (dimensions = 30).

**Figure 3:**
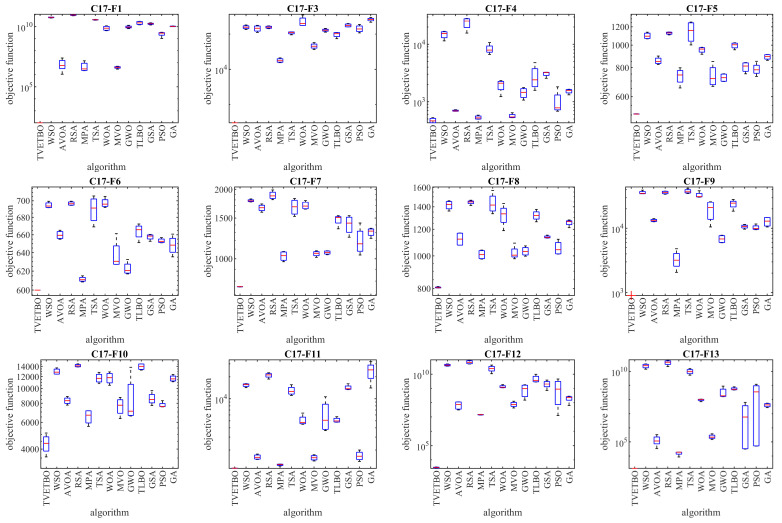

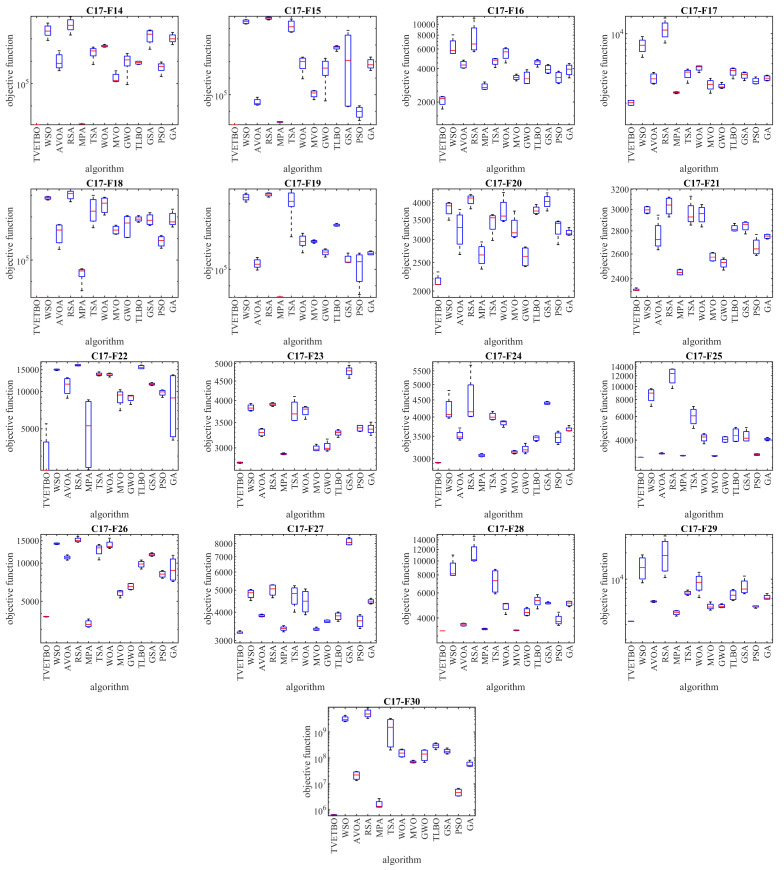
Boxplot diagrams of TVETBO and competitor algorithms’ performances on the CEC 2017 test suite (dimensions = 50).

**Figure 4:**
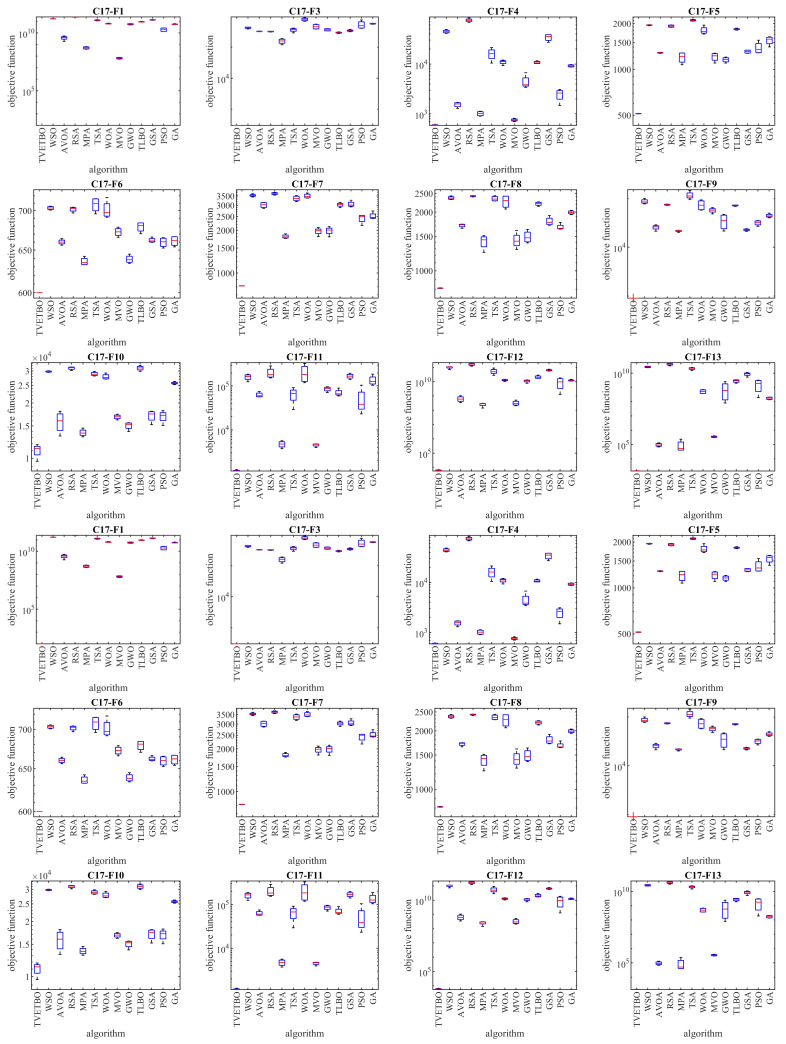

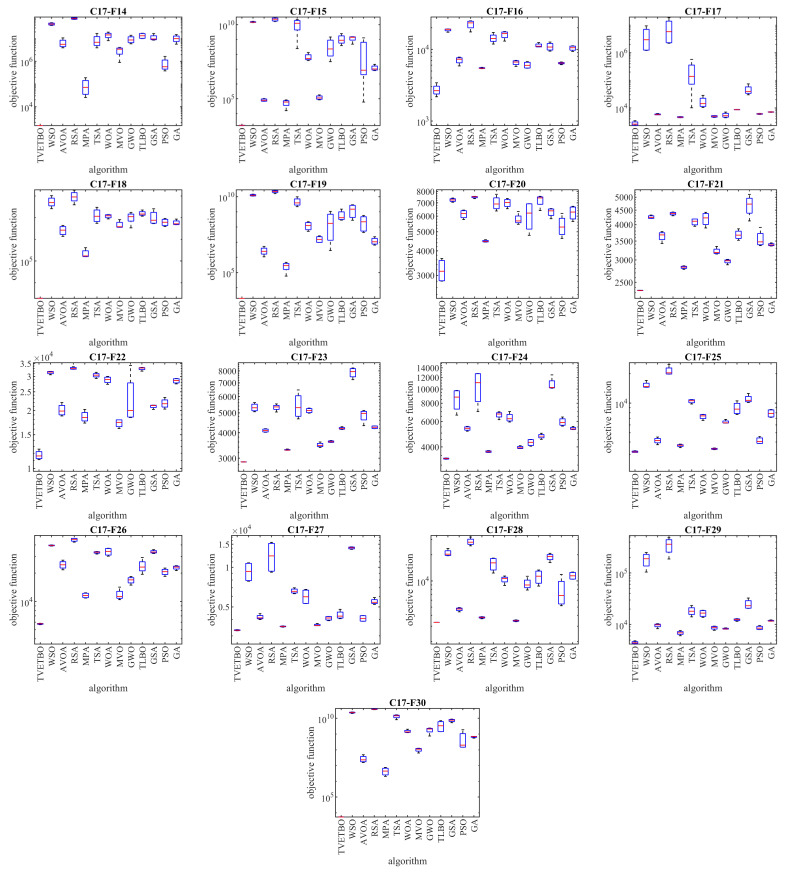
Boxplot diagrams of TVETBO and competitor algorithms’ performances on the CEC 2017 test suite (dimensions = 100).

In this subsection, the ability of the proposed approach to optimize the CEC 2017 test suite is measured. The CEC 2017 test suite has thirty benchmark functions consisting of three unimodal functions of C17-F1 to C17-F3, seven multimodal functions of C17-F4 to C17-F10, ten hybrid functions of C17-F11 to C17-F20, and ten composition functions of C17-F21 to C17-F30. The C17-F2 function was not included in the simulation studies due to its unstable behavior. The full description of the CEC 2017 test suite is provided in [74]. In these tests, with the aim of a scalability analysis, TVETBO and the competitor algorithms were employed to solve this test suite for dimensions of the problem (number of decision variables) equal to 10, 30, 50, and 100. The implementation results of the proposed TVETBO approach and the competitor algorithms on the CEC 2017 test suite are reported in Table 2, Table 3, Table 4 and Table 5. The boxplot diagrams resulting from the performance of the metaheuristic algorithms on this test suite are drawn in Figure 1, Figure 2, Figure 3 and Figure 4. The simulation results show that, for dimensions equal to 10, TVETBO is the best optimizer for handling C17-F1, C17-F3 to C17-F24, and C17-F27 to C17-F30. For dimensions equal to 30, TVETBO is the best optimizer to solve C17-F1, C17-F3 to C17-F5, C17-F7, C17-F12 to C17-F14, C17-F16 to C17-F18, C17-F21 to C17-F27, and C17-F29. For dimensions equal to 50, TVETBO is the best optimizer for handling C17-F1, C17-F3 to C17-F25, and C17-F27 to C17-F30. For dimensions equal to 100, TVETBO is the best optimizer to solve C17-F1, and C17-F3 to C17-F30.

The optimization results indicate that the proposed TVETBO approach with high abilities to explore, exploit, and create a balance between exploration and exploitation during the search process, is able to provide suitable results for the CEC 2017 test suite. Comparing the simulation results, it is shown that TVETBO provides better results in most of the benchmark functions for different dimensions of 10, 30, 50, and 100, compared to competitor algorithms when handling the CEC 2017 test suite.

### 4.3. Statistical Analysis

In this subsection, a statistical analysis on the performances of TVETBO and competing algorithms is presented to determine whether the superiority of the proposed approach is significant from a statistical point of view. For this purpose, the Wilcoxon rank sum test [75] is employed. This is a non-parametric test and is used to determine the significant difference between two data samples. In this test, using an index called the *p*-value, it is determined whether there is a significant difference between two data samples or not. The implementation results of the Wilcoxon rank sum test on TVETBO compared to each of the competitor algorithms are presented in Table 6. Based on the statistical analysis, in cases where the *p*-value is less than 0.05, TVETBO has a significant statistical superiority in the competition with the corresponding compared algorithm.

### 4.4. Discussion and Results Analysis

Metaheuristic algorithms are stochastic approaches that are able to achieve suitable solutions for optimization problems in an iteration-based process based on a random search of the problem-solving space. The mechanism of the search process in metaheuristic algorithms should be based on three principles: exploration, exploitation, and establishing a balance between exploration and exploitation during the search process.

Unimodal functions do not have any local optima, and for that reason, they are suitable options for evaluating the exploitation power of metaheuristic algorithms. The C17-F1 and C17-F3 functions are unimodal functions. Based on the optimization results reported in Table 2, Table 3, Table 4 and Table 5, for problem dimensions equal to 10, 30, 50, and 100, TVETBO is the best optimizer for the C17-F1 and C17-F3 functions and it achieves appropriate results. In addition, TVETBO provides a superior performance by achieving better results for unimodal functions compared to the competitor algorithm. What is evident from the analysis of the simulation results is that TVETBO has a high exploitation ability, so it can effectively manage the local search in the problem-solving space.

In addition to the global optimum, multimodal functions have several local optima, and for this reason, they are suitable options for evaluating the exploration power of metaheuristic algorithms. Functions C17-F4 to C17-F10 are multimodal functions. Based on the results reported in Table 2, Table 3, Table 4 and Table 5, TVETBO provides effective results for the handling of multimodal functions C17-F4 to C17-F10 for problem dimensions equal to 10, 30, 50, and 100. The analysis of the simulation results shows that TVETBO provides a superior performance in handling multimodal functions compared to the competing algorithms. The optimization results confirm that TVETBO has a high exploration ability, so it can manage the global search effectively in the problem-solving space.

Functions C17-F11 to C17-F30 from the CEC 2017 test suite are complex optimization problems that challenge the ability of metaheuristic algorithms to establish a balance between exploration and exploitation. Based on the simulation results reported in Table 2, Table 3, Table 4 and Table 5, TVETBO with its high ability to balance exploration and exploitation is able to identify the region containing the main optimum and converge towards solutions close to the global optimum. A comparison of simulation results shows that TVETBO is the best optimizer for most of these functions compared to competing algorithms. What is evident from the simulation results is that TVETBO provides a superior performance in terms of solving these functions and has a better ability to balance exploration and exploitation compared to competing algorithms.

The findings obtained from the analysis of the simulation results are that TVETBO has a high exploitation ability based on the optimization results of the C17-F1 and C17-F3 functions, a high exploration ability based on the optimization results of the C17-F4 to C17-F10 functions, and a high ability to balance exploitation and exploration based on the optimization results of functions C17-F11 to C17-F30.

A comparison of metaheuristic algorithms using statistical indicators provides valuable information about the performance of the metaheuristic algorithms. However, the use of statistical analysis shows whether the superiority of an algorithm over other algorithms is significant from a statistical point of view. The results obtained from the Wilcoxon rank sum test reported in Table 6 confirm that TVETBO has a significant statistical superiority compared to the competing algorithms in terms of handling the CEC 2017 test suite for problem dimensions equal to 10, 30, 50, and 100.

## 5. TVETBO for Real-World Applications

**Figure 5:**
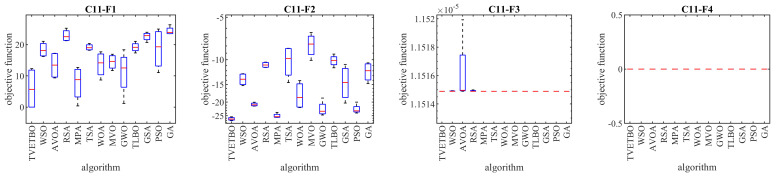

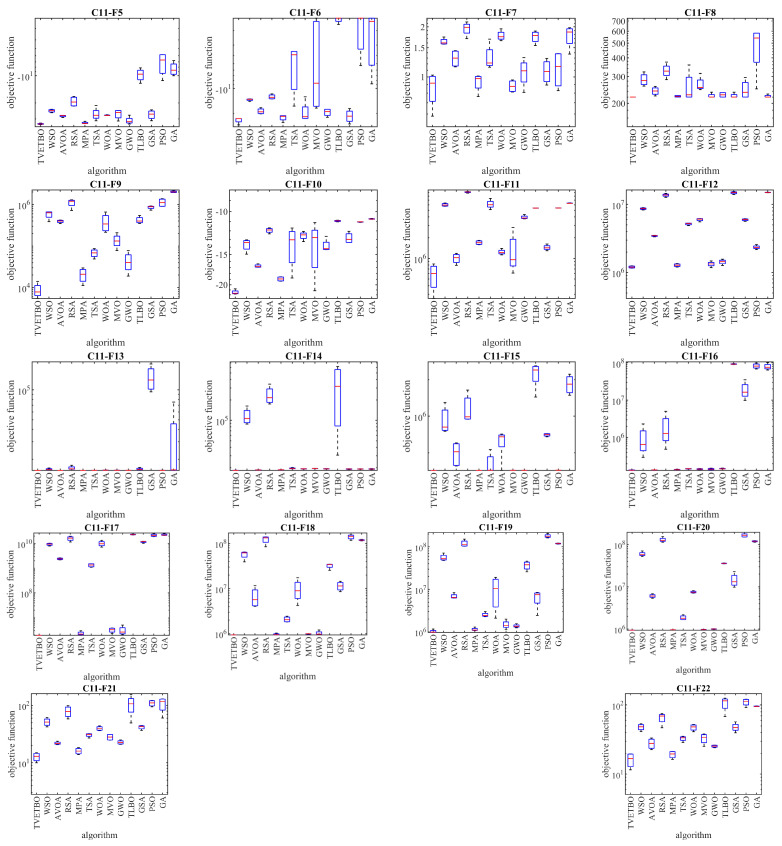
Boxplot diagrams of TVETBO and competitor algorithms’ performances on the CEC 2011 test suite.

In this section, the performance evaluation of TVETBO in terms of handling real-world applications is discussed. For this purpose, the CEC 2011 test suite is employed. This consists of twenty-two constrained optimization problems from real-world applications. A full description and details of the CEC 2011 test suite are provided in [76]. The implementation results of the TVETBO and competitor algorithms on the CEC 2011 test suite are reported in Table 7. The boxplot diagrams resulting from the performance of metaheuristic algorithms on this test suite are drawn in Figure 5. The optimization results show that TVETBO has an effective ability to solve real-world optimization problems. Based on the comparison of the simulation results, TVETBO is the best optimizer for optimization problems C11-F1 to C11-F22. The simulation results show that TVETBO provides better results for most of the optimization problems, and by winning first place, it provides a superior performance compared to the competitor algorithms. In addition, the results obtained from the statistical analysis of the Wilcoxon rank sum test, which are reported in the last row of Table 7, indicate that the superiority of TVETBO when optimizing the CEC 2011 test suite is significant compared to competitor algorithms from a statistical point of view.

## 6. Conclusions and Future Work

In this paper, a new human-based metaheuristic algorithm called Technical and Vocational Education and Training-Based Optimizer (TVETBO) was introduced by imitating the skill-training process used in technical and vocational education and training schools. The fundamental inspiration of TVETBO comes from the impact of technical and vocational education and training in terms of preparing young people for work based on training and improving work-related skills. The TVETBO theory was stated, and its implementation steps were mathematically modeled in three phases: (i) theory education, (ii) practical education, and (iii) individual skills development. The efficiency of TVETBO for handling optimization tasks was evaluated on the CEC 2017 test suite for problem dimensions equal to 10, 30, 50, and 100. The optimization results show that TVETBO has high abilities to explore, exploit, and balance exploration and exploitation during the search process. The efficiency of TVETBO when solving optimization problems was compared with the performances of twelve metaheuristic algorithms. Based on the simulation results, it was found that TVETBO, when handling the CEC 2017 test suite, provided better results for 27 out of 29 benchmark functions (i.e., 93.1%) for the problem with dimensions equal to 10, 19 out of 29 benchmark functions (i.e., 65.52%) for the problem with dimensions equal to 30, 28 out of 29 benchmark functions (i.e., 96.55%) for the problem with dimensions equal to 50, and 29 of the 29 benchmark functions (i.e., 100%) for the problem with dimensions equal to 100. Thus, it provided a superior performance in comparison with the alternative algorithms. The simulation results show that TVETBO has provided a superior performance by providing better results for most of the benchmark functions compared to the competitor algorithms. In addition, in order to evaluate the performance of TVETBO in dealing with real-world applications, a set of twenty-two constrained optimization problems was selected from the CEC 2011 test suite under the headings parameter estimation for frequency-modulated (FM) sound waves, the Lennard–Jones potential problem, the bifunctional catalyst blend optimal control problem, optimal control of a non-linear stirred tank reactor, Tersoff potential for model Si (B), Tersoff potential for model Si (C), spread spectrum radar polyphase code design, the transmission network expansion planning (TNEP) problem, the large-scale transmission pricing problem, the circular antenna array design problem, the ELD problems (consisting of: DED instance 1, DED instance 2, ELD Instance 1, ELD Instance 2, ELD Instance 3, ELD Instance 4, ELD Instance 5, hydrothermal scheduling instance 1, hydrothermal scheduling instance 2, hydrothermal scheduling instance 3), the messenger: spacecraft trajectory optimization problem, and the cassini 2: spacecraft trajectory optimization problem. The results obtained from the optimization of the CEC 2011 test suite showed the appropriate efficiency of the proposed approach when handling optimization problems in real-world applications.

After introducing the proposed TVETBO approach, several research paths were activated for further studies. One of the most special is the design of binary and multi-objective versions of TVETBO. Applying TVETBO to solve optimization problems in different sciences and other real-world applications are other suggestions for future studies.

## Figures and Tables

**Table 1:** Control parameters’ values.

Algorithm	Parameter	Value
GA		
	Type	Real coded
	Selection	Roulette wheel (proportionate)
	Crossover	Whole arithmetic (probability = 0.8, α∈[−0.5, 1.5])
	Mutation	Gaussian (probability = 0.05)
PSO		
	Topology	Fully connected
	Cognitive and social constant	(*C*_1_, *C*_2_)=(2, 2)
	Inertia weight	Linear reduction from 0.9 to 0.1
	Velocity limit	10% of the dimension range
GSA		
	Alpha, *G*_0_, *Rnorm*, *Rpower*	20, 100, 2, 1
TLBO		
	*T_F_*: teaching factor	*T_F_* = round [(1+rand)]
	Random number	*rand* is a random number in [0−1].
GWO		
	Convergence parameter (*a*)	*a*: Linear reduction from 2 to 0.
MVO		
	Wormhole existence probability (WEP)	Min(WEP) = 0.2 and Max(WEP)=1.
	Exploitation accuracy over the iterations (*p*)	p=6.
WOA		
	Convergence parameter (*a*)	*a*: Linear reduction from 2 to 0.
	*r* is a random vector in [0−1].	
	*l* is a random number in [−1,1].	
TSA		
	Pmin and Pmax	1, 4
	*c1, c2, c3*	Random numbers lie in the range of [0−1].
MPA		
	Constant number	*P* = 0.5
	Random vector	*R* is a vector of uniform random numbers in [0, 1].
	Fish Aggregating Devices (*FADs*)	*FADs*=0.2
	Binary vector	*U*= 0 or 1
RSA		
	Sensitive parameter	β=0.01
	Sensitive parameter	α=0.1
	Evolutionary Sense (ES)	ES: Randomly decreasing values between 2 and −2
AVOA		
	L_1_, L_2_	0.8, 0.2
	w	2.5
	P_1_, P_2_, P_3_	0.6, 0.4, 0.6
WSO		
	F_min_ and F_max_	0.07, 0.75
	*τ, a_o_, a_1_, a_2_*	4.125, 6.25, 100, 0.0005

**Table 2:** Optimization results of the CEC 2017 test suite (dimensions = 10).

		TVETBO	WSO	AVOA	RSA	MPA	TSA	WOA	MVO	GWO	TLBO	GSA	PSO	GA
C17-F1	mean	1.00E+02	5.63E+09	3.85E+03	1.02E+10	3.53E+07	1.74E+09	6.46E+06	7.53E+03	8.83E+07	1.47E+08	7.47E+02	3.15E+03	1.19E+07
	best	1.00E+02	4.67E+09	1.16E+02	8.83E+09	1.12E+04	3.73E+08	4.70E+06	4.79E+03	2.78E+04	6.56E+07	1.00E+02	3.46E+02	6.14E+06
	worst	1.00E+02	7.23E+09	1.19E+04	1.22E+10	1.28E+08	3.79E+09	8.50E+06	1.11E+04	3.21E+08	3.55E+08	1.79E+03	9.32E+03	1.70E+07
	std	0.00E+00	1.21E+09	6.00E+03	1.64E+09	6.77E+07	1.66E+09	1.75E+06	3.21E+03	1.69E+08	1.52E+08	7.96E+02	4.52E+03	4.95E+06
	median	1.00E+02	5.32E+09	1.67E+03	9.94E+09	6.47E+06	1.40E+09	6.31E+06	7.11E+03	1.62E+07	8.41E+07	5.48E+02	1.46E+03	1.21E+07
	rank	1	12	4	13	8	11	6	5	9	10	2	3	7
C17-F3	mean	3.00E+02	8.54E+03	3.02E+02	9.65E+03	1.41E+03	1.12E+04	1.73E+03	3.00E+02	3.07E+03	7.27E+02	1.03E+04	3.00E+02	1.48E+04
	best	3.00E+02	4.32E+03	3.00E+02	5.20E+03	7.92E+02	4.27E+03	6.19E+02	3.00E+02	1.53E+03	4.71E+02	6.46E+03	3.00E+02	4.35E+03
	worst	3.00E+02	1.14E+04	3.04E+02	1.29E+04	2.54E+03	1.58E+04	3.33E+03	3.00E+02	5.89E+03	8.93E+02	1.40E+04	3.00E+02	2.34E+04
	std	0.00E+00	3.38E+03	2.39E+00	3.84E+03	8.76E+02	5.35E+03	1.39E+03	5.34E-02	2.19E+03	2.01E+02	3.36E+03	5.05E-14	1.08E+04
	median	3.00E+02	9.20E+03	3.02E+02	1.02E+04	1.15E+03	1.24E+04	1.49E+03	3.00E+02	2.43E+03	7.71E+02	1.03E+04	3.00E+02	1.57E+04
	rank	1	9	4	10	6	12	7	3	8	5	11	2	13
C17-F4	mean	4.00E+02	9.34E+02	4.05E+02	1.35E+03	4.07E+02	5.77E+02	4.25E+02	4.03E+02	4.12E+02	4.09E+02	4.05E+02	4.20E+02	4.15E+02
	best	4.00E+02	6.96E+02	4.01E+02	8.46E+02	4.02E+02	4.78E+02	4.06E+02	4.02E+02	4.06E+02	4.08E+02	4.04E+02	4.00E+02	4.12E+02
	worst	4.00E+02	1.15E+03	4.07E+02	1.85E+03	4.11E+02	6.92E+02	4.74E+02	4.05E+02	4.28E+02	4.10E+02	4.06E+02	4.70E+02	4.18E+02
	std	0.00E+00	2.25E+02	2.71E+00	4.66E+02	4.80E+00	1.14E+02	3.53E+01	1.87E+00	1.21E+01	5.98E-01	1.26E+00	3.67E+01	3.22E+00
	median	4.00E+02	9.46E+02	4.06E+02	1.36E+03	4.07E+02	5.68E+02	4.10E+02	4.03E+02	4.06E+02	4.09E+02	4.04E+02	4.05E+02	4.14E+02
	rank	1	12	4	13	5	11	10	2	7	6	3	9	8
C17-F5	mean	5.01E+02	5.65E+02	5.45E+02	5.74E+02	5.13E+02	5.65E+02	5.41E+02	5.24E+02	5.13E+02	5.34E+02	5.54E+02	5.28E+02	5.28E+02
	best	5.01E+02	5.50E+02	5.27E+02	5.59E+02	5.08E+02	5.44E+02	5.24E+02	5.10E+02	5.09E+02	5.29E+02	5.50E+02	5.11E+02	5.24E+02
	worst	5.02E+02	5.74E+02	5.64E+02	5.89E+02	5.18E+02	5.97E+02	5.78E+02	5.38E+02	5.21E+02	5.38E+02	5.66E+02	5.52E+02	5.34E+02
	std	5.41E-01	1.20E+01	2.08E+01	1.81E+01	5.59E+00	2.59E+01	2.75E+01	1.27E+01	5.60E+00	4.36E+00	8.74E+00	2.06E+01	5.20E+00
	median	5.01E+02	5.67E+02	5.44E+02	5.73E+02	5.13E+02	5.60E+02	5.32E+02	5.24E+02	5.12E+02	5.35E+02	5.51E+02	5.25E+02	5.28E+02
	rank	1	11	9	13	2	12	8	4	3	7	10	5	6
C17-F6	mean	6.00E+02	6.33E+02	6.18E+02	6.41E+02	6.01E+02	6.25E+02	6.24E+02	6.02E+02	6.01E+02	6.07E+02	6.17E+02	6.08E+02	6.10E+02
	best	6.00E+02	6.29E+02	6.17E+02	6.38E+02	6.01E+02	6.15E+02	6.08E+02	6.00E+02	6.01E+02	6.05E+02	6.03E+02	6.01E+02	6.07E+02
	worst	6.00E+02	6.36E+02	6.20E+02	6.46E+02	6.02E+02	6.41E+02	6.46E+02	6.04E+02	6.02E+02	6.10E+02	6.37E+02	6.20E+02	6.15E+02
	std	0.00E+00	3.75E+00	1.88E+00	3.71E+00	8.89E-01	1.21E+01	1.75E+01	1.91E+00	5.13E-01	2.71E+00	1.70E+01	8.97E+00	3.72E+00
	median	6.00E+02	6.33E+02	6.17E+02	6.41E+02	6.01E+02	6.22E+02	6.20E+02	6.02E+02	6.01E+02	6.06E+02	6.15E+02	6.05E+02	6.10E+02
	rank	1	12	9	13	3	11	10	4	2	5	8	6	7
C17-F7	mean	7.11E+02	8.05E+02	7.66E+02	8.06E+02	7.25E+02	8.30E+02	7.63E+02	7.31E+02	7.26E+02	7.53E+02	7.17E+02	7.33E+02	7.37E+02
	best	7.11E+02	7.84E+02	7.44E+02	7.92E+02	7.21E+02	7.90E+02	7.52E+02	7.17E+02	7.18E+02	7.48E+02	7.15E+02	7.26E+02	7.27E+02
	worst	7.12E+02	8.21E+02	7.95E+02	8.19E+02	7.29E+02	8.72E+02	7.93E+02	7.51E+02	7.44E+02	7.61E+02	7.21E+02	7.45E+02	7.42E+02
	std	5.57E-01	1.72E+01	2.51E+01	1.34E+01	4.02E+00	3.91E+01	2.17E+01	1.53E+01	1.32E+01	6.26E+00	2.89E+00	9.44E+00	7.75E+00
	median	7.11E+02	8.06E+02	7.63E+02	8.06E+02	7.25E+02	8.30E+02	7.54E+02	7.28E+02	7.22E+02	7.51E+02	7.16E+02	7.31E+02	7.40E+02
	rank	1	11	10	12	3	13	9	5	4	8	2	6	7
C17-F8	mean	8.01E+02	8.49E+02	8.32E+02	8.55E+02	8.13E+02	8.49E+02	8.37E+02	8.12E+02	8.16E+02	8.38E+02	8.20E+02	8.23E+02	8.17E+02
	best	8.01E+02	8.41E+02	8.21E+02	8.43E+02	8.09E+02	8.33E+02	8.19E+02	8.08E+02	8.11E+02	8.31E+02	8.12E+02	8.16E+02	8.13E+02
	worst	8.02E+02	8.58E+02	8.48E+02	8.60E+02	8.15E+02	8.69E+02	8.49E+02	8.17E+02	8.21E+02	8.46E+02	8.28E+02	8.30E+02	8.25E+02
	std	6.25E-01	8.28E+00	1.24E+01	8.40E+00	3.04E+00	1.75E+01	1.42E+01	4.18E+00	4.77E+00	8.42E+00	7.35E+00	7.40E+00	5.86E+00
	median	8.01E+02	8.49E+02	8.29E+02	8.58E+02	8.14E+02	8.47E+02	8.40E+02	8.12E+02	8.16E+02	8.38E+02	8.20E+02	8.23E+02	8.15E+02
	rank	1	12	8	13	3	11	9	2	4	10	6	7	5
C17-F9	mean	9.00E+02	1.43E+03	1.19E+03	1.48E+03	9.05E+02	1.39E+03	1.38E+03	9.01E+02	9.12E+02	9.12E+02	9.00E+02	9.04E+02	9.05E+02
	best	9.00E+02	1.29E+03	9.55E+02	1.38E+03	9.00E+02	1.17E+03	1.08E+03	9.00E+02	9.01E+02	9.07E+02	9.00E+02	9.01E+02	9.03E+02
	worst	9.00E+02	1.57E+03	1.67E+03	1.62E+03	9.14E+02	1.68E+03	1.67E+03	9.03E+02	9.34E+02	9.20E+02	9.00E+02	9.13E+02	9.09E+02
	std	0.00E+00	1.41E+02	3.62E+02	1.10E+02	6.47E+00	2.40E+02	2.71E+02	1.70E+00	1.69E+01	6.20E+00	0.00E+00	6.03E+00	3.14E+00
	median	9.00E+02	1.43E+03	1.07E+03	1.46E+03	9.04E+02	1.35E+03	1.39E+03	9.00E+02	9.07E+02	9.10E+02	9.00E+02	9.02E+02	9.04E+02
	rank	1	11	8	12	5	10	9	2	7	6	1	3	4
C17-F10	mean	1.01E+03	2.31E+03	1.78E+03	2.59E+03	1.52E+03	2.04E+03	2.03E+03	1.79E+03	1.73E+03	2.18E+03	2.29E+03	1.95E+03	1.72E+03
	best	1.00E+03	2.05E+03	1.49E+03	2.42E+03	1.39E+03	1.76E+03	1.45E+03	1.46E+03	1.54E+03	1.79E+03	2.00E+03	1.56E+03	1.42E+03
	worst	1.01E+03	2.50E+03	2.42E+03	2.95E+03	1.60E+03	2.29E+03	2.56E+03	2.29E+03	2.00E+03	2.47E+03	2.39E+03	2.36E+03	2.12E+03
	std	7.24E+00	2.21E+02	4.78E+02	2.71E+02	1.03E+02	3.05E+02	5.82E+02	4.38E+02	2.11E+02	3.16E+02	2.05E+02	3.56E+02	3.27E+02
	median	1.01E+03	2.35E+03	1.61E+03	2.49E+03	1.54E+03	2.05E+03	2.06E+03	1.70E+03	1.69E+03	2.23E+03	2.37E+03	1.94E+03	1.67E+03
	rank	1	12	5	13	2	9	8	6	4	10	11	7	3
C17-F11	mean	1.10E+03	3.87E+03	1.15E+03	4.00E+03	1.13E+03	5.48E+03	1.15E+03	1.13E+03	1.16E+03	1.15E+03	1.14E+03	1.14E+03	2.39E+03
	best	1.10E+03	2.62E+03	1.12E+03	1.46E+03	1.11E+03	5.33E+03	1.11E+03	1.11E+03	1.12E+03	1.14E+03	1.12E+03	1.13E+03	1.12E+03
	worst	1.10E+03	5.08E+03	1.20E+03	6.51E+03	1.16E+03	5.56E+03	1.17E+03	1.15E+03	1.23E+03	1.17E+03	1.17E+03	1.17E+03	6.00E+03
	std	0.00E+00	1.20E+03	4.08E+01	2.47E+03	2.35E+01	1.12E+02	3.04E+01	2.37E+01	5.44E+01	1.63E+01	2.29E+01	1.61E+01	2.62E+03
	median	1.10E+03	3.90E+03	1.14E+03	4.01E+03	1.12E+03	5.52E+03	1.16E+03	1.13E+03	1.14E+03	1.15E+03	1.13E+03	1.14E+03	1.22E+03
	rank	1	11	6	12	2	13	8	3	9	7	4	5	10
C17-F12	mean	1.35E+03	3.56E+08	1.11E+06	7.11E+08	5.72E+05	1.05E+06	2.37E+06	1.04E+06	1.43E+06	5.09E+06	1.03E+06	8.14E+03	6.10E+05
	best	1.32E+03	7.98E+07	3.59E+05	1.58E+08	2.00E+04	5.43E+05	1.73E+05	8.89E+03	4.58E+04	1.36E+06	4.78E+05	2.53E+03	1.77E+05
	worst	1.44E+03	6.22E+08	2.01E+06	1.24E+09	8.95E+05	1.29E+06	3.94E+06	3.26E+06	2.23E+06	9.01E+06	1.74E+06	1.40E+04	1.08E+06
	std	6.23E+01	2.98E+08	8.41E+05	5.97E+08	4.19E+05	3.81E+05	1.90E+06	1.63E+06	1.05E+06	4.41E+06	5.81E+05	5.69E+03	4.02E+05
	median	1.33E+03	3.61E+08	1.03E+06	7.21E+08	6.86E+05	1.18E+06	2.69E+06	4.41E+05	1.71E+06	5.00E+06	9.48E+05	8.01E+03	5.93E+05
	rank	1	12	8	13	3	7	10	6	9	11	5	2	4
C17-F13	mean	1.31E+03	1.73E+07	1.85E+04	3.46E+07	5.47E+03	1.28E+04	7.63E+03	6.77E+03	1.04E+04	1.68E+04	1.01E+04	6.66E+03	5.49E+04
	best	1.30E+03	1.45E+06	2.73E+03	2.88E+06	3.74E+03	7.64E+03	3.30E+03	1.39E+03	6.55E+03	1.59E+04	5.08E+03	2.39E+03	8.60E+03
	worst	1.31E+03	5.75E+07	3.16E+04	1.15E+08	6.69E+03	2.03E+04	1.53E+04	1.25E+04	1.45E+04	1.91E+04	1.43E+04	1.68E+04	1.81E+05
	std	2.47E+00	2.92E+07	1.63E+04	5.84E+07	1.53E+03	5.96E+03	5.93E+03	6.24E+03	3.54E+03	1.68E+03	4.23E+03	7.46E+03	9.18E+04
	median	1.30E+03	5.18E+06	1.97E+04	1.03E+07	5.72E+03	1.17E+04	5.98E+03	6.61E+03	1.02E+04	1.62E+04	1.06E+04	3.71E+03	1.47E+04
	rank	1	12	10	13	2	8	5	4	7	9	6	3	11
C17-F14	mean	1.40E+03	4.02E+03	2.03E+03	5.38E+03	1.94E+03	3.40E+03	1.52E+03	1.57E+03	2.35E+03	1.59E+03	5.60E+03	3.01E+03	1.31E+04
	best	1.40E+03	3.17E+03	1.68E+03	4.71E+03	1.44E+03	1.49E+03	1.48E+03	1.42E+03	1.46E+03	1.52E+03	4.63E+03	1.43E+03	3.75E+03
	worst	1.40E+03	5.47E+03	2.84E+03	6.95E+03	2.92E+03	5.62E+03	1.56E+03	2.00E+03	4.99E+03	1.62E+03	7.61E+03	6.89E+03	2.60E+04
	std	5.41E-01	1.16E+03	5.94E+02	1.14E+03	7.56E+02	2.39E+03	4.32E+01	3.09E+02	1.91E+03	5.49E+01	1.52E+03	2.84E+03	1.03E+04
	median	1.40E+03	3.71E+03	1.79E+03	4.94E+03	1.71E+03	3.25E+03	1.52E+03	1.44E+03	1.48E+03	1.61E+03	5.09E+03	1.86E+03	1.12E+04
	rank	1	10	6	11	5	9	2	3	7	4	12	8	13
C17-F15	mean	1.50E+03	1.04E+04	5.33E+03	1.40E+04	4.00E+03	7.05E+03	6.26E+03	1.54E+03	5.85E+03	1.71E+03	2.41E+04	9.06E+03	4.58E+03
	best	1.50E+03	3.02E+03	2.08E+03	2.74E+03	3.24E+03	2.33E+03	2.02E+03	1.53E+03	3.59E+03	1.58E+03	1.13E+04	2.88E+03	1.89E+03
	worst	1.50E+03	1.82E+04	1.27E+04	3.06E+04	4.92E+03	1.26E+04	1.36E+04	1.55E+03	6.95E+03	1.80E+03	3.61E+04	1.49E+04	8.07E+03
	std	2.56E-01	6.88E+03	5.40E+03	1.32E+04	7.60E+02	4.82E+03	5.47E+03	1.34E+01	1.68E+03	1.16E+02	1.29E+04	5.47E+03	3.34E+03
	median	1.50E+03	1.01E+04	3.26E+03	1.13E+04	3.91E+03	6.62E+03	4.73E+03	1.54E+03	6.44E+03	1.73E+03	2.44E+04	9.23E+03	4.17E+03
	rank	1	11	6	12	4	9	8	2	7	3	13	10	5
C17-F16	mean	1.60E+03	2.02E+03	1.81E+03	2.02E+03	1.68E+03	2.05E+03	1.95E+03	1.82E+03	1.73E+03	1.68E+03	2.08E+03	1.93E+03	1.80E+03
	best	1.60E+03	1.94E+03	1.64E+03	1.82E+03	1.64E+03	1.86E+03	1.77E+03	1.73E+03	1.62E+03	1.65E+03	1.95E+03	1.82E+03	1.72E+03
	worst	1.60E+03	2.17E+03	1.93E+03	2.30E+03	1.72E+03	2.24E+03	2.08E+03	1.88E+03	1.83E+03	1.73E+03	2.27E+03	2.09E+03	1.84E+03
	std	3.44E-01	1.15E+02	1.31E+02	2.18E+02	3.45E+01	1.84E+02	1.64E+02	7.03E+01	9.49E+01	4.10E+01	1.60E+02	1.33E+02	6.12E+01
	median	1.60E+03	1.97E+03	1.84E+03	1.98E+03	1.69E+03	2.05E+03	1.98E+03	1.83E+03	1.74E+03	1.66E+03	2.04E+03	1.90E+03	1.83E+03
	rank	1	10	6	11	3	12	9	7	4	2	13	8	5
C17-F17	mean	1.70E+03	1.82E+03	1.75E+03	1.82E+03	1.74E+03	1.80E+03	1.84E+03	1.84E+03	1.77E+03	1.76E+03	1.85E+03	1.75E+03	1.76E+03
	best	1.70E+03	1.81E+03	1.73E+03	1.80E+03	1.72E+03	1.79E+03	1.77E+03	1.78E+03	1.72E+03	1.75E+03	1.75E+03	1.75E+03	1.75E+03
	worst	1.70E+03	1.82E+03	1.80E+03	1.83E+03	1.78E+03	1.81E+03	1.89E+03	1.95E+03	1.87E+03	1.77E+03	1.98E+03	1.76E+03	1.76E+03
	std	1.69E-01	7.03E+00	3.23E+01	1.28E+01	2.87E+01	1.23E+01	5.52E+01	8.94E+01	7.58E+01	1.09E+01	1.26E+02	6.26E+00	2.76E+00
	median	1.70E+03	1.82E+03	1.74E+03	1.82E+03	1.72E+03	1.81E+03	1.85E+03	1.82E+03	1.74E+03	1.76E+03	1.83E+03	1.75E+03	1.76E+03
	rank	1	9	3	10	2	8	11	12	7	6	13	4	5
C17-F18	mean	1.81E+03	2.87E+06	1.19E+04	5.73E+06	1.11E+04	1.21E+04	2.34E+04	2.11E+04	2.00E+04	2.97E+04	9.76E+03	2.20E+04	1.29E+04
	best	1.80E+03	1.47E+05	4.86E+03	2.84E+05	4.17E+03	7.50E+03	6.48E+03	8.74E+03	6.35E+03	2.41E+04	6.42E+03	2.89E+03	3.45E+03
	worst	1.82E+03	8.33E+06	1.57E+04	1.66E+07	1.66E+04	1.64E+04	3.68E+04	3.39E+04	3.38E+04	3.71E+04	1.19E+04	4.10E+04	1.86E+04
	std	1.09E+01	4.12E+06	5.28E+03	8.25E+06	6.15E+03	4.02E+03	1.59E+04	1.29E+04	1.51E+04	6.50E+03	2.55E+03	2.14E+04	7.19E+03
	median	1.80E+03	1.51E+06	1.36E+04	3.00E+06	1.18E+04	1.23E+04	2.52E+04	2.08E+04	2.00E+04	2.87E+04	1.04E+04	2.21E+04	1.47E+04
	rank	1	12	4	13	3	5	10	8	7	11	2	9	6
C17-F19	mean	1.90E+03	3.99E+05	6.74E+03	7.08E+05	5.62E+03	1.26E+05	3.50E+04	1.91E+03	5.41E+03	4.71E+03	4.07E+04	2.51E+04	6.21E+03
	best	1.90E+03	2.52E+04	2.18E+03	4.61E+04	2.32E+03	1.95E+03	7.69E+03	1.91E+03	1.94E+03	2.04E+03	1.12E+04	2.63E+03	2.22E+03
	worst	1.90E+03	8.43E+05	1.33E+04	1.52E+06	9.46E+03	2.52E+05	6.41E+04	1.92E+03	1.39E+04	1.26E+04	5.90E+04	7.73E+04	9.93E+03
	std	8.10E-01	3.84E+05	5.89E+03	7.24E+05	3.96E+03	1.56E+05	2.52E+04	7.70E+00	6.21E+03	5.69E+03	2.33E+04	3.83E+04	3.46E+03
	median	1.90E+03	3.64E+05	5.73E+03	6.33E+05	5.35E+03	1.25E+05	3.41E+04	1.91E+03	2.90E+03	2.13E+03	4.62E+04	1.02E+04	6.34E+03
	rank	1	12	7	13	5	11	9	2	4	3	10	8	6
C17-F20	mean	2.00E+03	2.22E+03	2.17E+03	2.22E+03	2.09E+03	2.21E+03	2.21E+03	2.14E+03	2.17E+03	2.07E+03	2.26E+03	2.17E+03	2.05E+03
	best	2.00E+03	2.16E+03	2.03E+03	2.17E+03	2.07E+03	2.11E+03	2.10E+03	2.05E+03	2.13E+03	2.06E+03	2.19E+03	2.15E+03	2.04E+03
	worst	2.00E+03	2.29E+03	2.30E+03	2.28E+03	2.12E+03	2.32E+03	2.29E+03	2.25E+03	2.25E+03	2.08E+03	2.35E+03	2.20E+03	2.06E+03
	std	0.00E+00	5.74E+01	1.30E+02	6.14E+01	2.35E+01	9.93E+01	9.92E+01	9.01E+01	5.68E+01	9.84E+00	8.47E+01	3.04E+01	1.12E+01
	median	2.00E+03	2.21E+03	2.18E+03	2.23E+03	2.09E+03	2.20E+03	2.22E+03	2.13E+03	2.15E+03	2.07E+03	2.24E+03	2.17E+03	2.05E+03
	rank	1	11	8	12	4	10	9	5	7	3	13	6	2
C17-F21	mean	2.20E+03	2.29E+03	2.21E+03	2.27E+03	2.26E+03	2.33E+03	2.31E+03	2.25E+03	2.31E+03	2.30E+03	2.37E+03	2.32E+03	2.30E+03
	best	2.20E+03	2.25E+03	2.20E+03	2.22E+03	2.26E+03	2.22E+03	2.22E+03	2.20E+03	2.31E+03	2.20E+03	2.35E+03	2.31E+03	2.23E+03
	worst	2.20E+03	2.32E+03	2.24E+03	2.29E+03	2.26E+03	2.37E+03	2.36E+03	2.31E+03	2.32E+03	2.34E+03	2.39E+03	2.33E+03	2.33E+03
	std	0.00E+00	3.75E+01	1.85E+01	3.28E+01	2.33E+00	7.72E+01	6.76E+01	6.72E+01	4.13E+00	7.06E+01	1.59E+01	8.41E+00	5.30E+01
	median	2.20E+03	2.30E+03	2.21E+03	2.28E+03	2.26E+03	2.35E+03	2.33E+03	2.25E+03	2.31E+03	2.33E+03	2.37E+03	2.32E+03	2.32E+03
	rank	1	6	2	5	4	12	9	3	10	8	13	11	7
C17-F22	mean	2.30E+03	2.74E+03	2.31E+03	2.92E+03	2.31E+03	2.72E+03	2.32E+03	2.29E+03	2.31E+03	2.32E+03	2.30E+03	2.31E+03	2.32E+03
	best	2.30E+03	2.61E+03	2.30E+03	2.71E+03	2.30E+03	2.45E+03	2.32E+03	2.23E+03	2.30E+03	2.31E+03	2.30E+03	2.30E+03	2.32E+03
	worst	2.30E+03	2.88E+03	2.31E+03	3.07E+03	2.31E+03	2.93E+03	2.33E+03	2.31E+03	2.32E+03	2.33E+03	2.30E+03	2.35E+03	2.32E+03
	std	1.58E-01	1.34E+02	3.42E+00	1.67E+02	3.89E+00	2.31E+02	6.03E+00	4.12E+01	1.07E+01	9.03E+00	9.74E-03	2.36E+01	3.45E+00
	median	2.30E+03	2.73E+03	2.31E+03	2.95E+03	2.30E+03	2.75E+03	2.32E+03	2.30E+03	2.31E+03	2.32E+03	2.30E+03	2.30E+03	2.32E+03
	rank	3	12	6	13	4	11	10	1	5	9	2	7	8
C17-F23	mean	2.60E+03	2.70E+03	2.64E+03	2.70E+03	2.61E+03	2.72E+03	2.65E+03	2.62E+03	2.61E+03	2.64E+03	2.79E+03	2.64E+03	2.66E+03
	best	2.60E+03	2.66E+03	2.63E+03	2.67E+03	2.61E+03	2.63E+03	2.63E+03	2.61E+03	2.61E+03	2.63E+03	2.73E+03	2.64E+03	2.64E+03
	worst	2.60E+03	2.72E+03	2.66E+03	2.74E+03	2.62E+03	2.77E+03	2.67E+03	2.63E+03	2.62E+03	2.65E+03	2.93E+03	2.66E+03	2.67E+03
	std	1.44E+00	3.39E+01	1.52E+01	3.57E+01	2.68E+00	6.62E+01	2.25E+01	1.18E+01	7.17E+00	9.82E+00	1.05E+02	9.51E+00	1.48E+01
	median	2.60E+03	2.71E+03	2.64E+03	2.70E+03	2.61E+03	2.75E+03	2.65E+03	2.62E+03	2.61E+03	2.64E+03	2.76E+03	2.64E+03	2.66E+03
	rank	1	10	5	11	3	12	8	4	2	6	13	7	9
C17-F24	mean	2.63E+03	2.78E+03	2.77E+03	2.85E+03	2.63E+03	2.67E+03	2.76E+03	2.68E+03	2.75E+03	2.76E+03	2.75E+03	2.77E+03	2.72E+03
	best	2.52E+03	2.72E+03	2.74E+03	2.83E+03	2.62E+03	2.52E+03	2.74E+03	2.50E+03	2.73E+03	2.75E+03	2.50E+03	2.76E+03	2.54E+03
	worst	2.73E+03	2.86E+03	2.79E+03	2.91E+03	2.64E+03	2.81E+03	2.79E+03	2.76E+03	2.77E+03	2.77E+03	2.90E+03	2.79E+03	2.81E+03
	std	1.27E+02	7.18E+01	2.79E+01	4.48E+01	9.60E+00	1.68E+02	2.51E+01	1.33E+02	1.92E+01	1.07E+01	1.86E+02	1.51E+01	1.38E+02
	median	2.64E+03	2.77E+03	2.77E+03	2.83E+03	2.63E+03	2.67E+03	2.76E+03	2.74E+03	2.75E+03	2.76E+03	2.80E+03	2.76E+03	2.77E+03
	rank	1	12	11	13	2	3	9	4	7	8	6	10	5
C17-F25	mean	2.93E+03	3.16E+03	2.91E+03	3.28E+03	2.92E+03	3.14E+03	2.91E+03	2.92E+03	2.94E+03	2.93E+03	2.92E+03	2.92E+03	2.95E+03
	best	2.90E+03	3.07E+03	2.90E+03	3.21E+03	2.91E+03	2.91E+03	2.76E+03	2.90E+03	2.92E+03	2.92E+03	2.90E+03	2.90E+03	2.94E+03
	worst	2.95E+03	3.37E+03	2.95E+03	3.36E+03	2.92E+03	3.66E+03	2.96E+03	2.94E+03	2.95E+03	2.95E+03	2.94E+03	2.95E+03	2.96E+03
	std	2.51E+01	1.52E+02	2.60E+01	6.58E+01	4.45E+00	3.88E+02	1.05E+02	2.68E+01	1.28E+01	2.20E+01	2.49E+01	2.86E+01	1.13E+01
	median	2.94E+03	3.10E+03	2.90E+03	3.28E+03	2.92E+03	2.99E+03	2.95E+03	2.92E+03	2.94E+03	2.93E+03	2.92E+03	2.92E+03	2.95E+03
	rank	7	12	2	13	3	11	1	4	9	8	5	6	10
C17-F26	mean	2.90E+03	3.61E+03	2.98E+03	3.76E+03	3.01E+03	3.63E+03	3.19E+03	2.90E+03	3.27E+03	3.21E+03	3.87E+03	2.90E+03	2.90E+03
	best	2.90E+03	3.26E+03	2.81E+03	3.44E+03	2.89E+03	3.15E+03	2.93E+03	2.90E+03	2.97E+03	2.91E+03	2.81E+03	2.81E+03	2.71E+03
	worst	2.90E+03	3.85E+03	3.16E+03	4.10E+03	3.30E+03	4.28E+03	3.60E+03	2.90E+03	3.92E+03	3.88E+03	4.36E+03	3.01E+03	3.11E+03
	std	4.04E-13	3.11E+02	2.19E+02	3.13E+02	2.07E+02	6.04E+02	3.20E+02	3.93E-02	4.74E+02	4.93E+02	7.84E+02	9.08E+01	2.24E+02
	median	2.90E+03	3.66E+03	2.98E+03	3.76E+03	2.93E+03	3.54E+03	3.11E+03	2.90E+03	3.09E+03	3.02E+03	4.16E+03	2.90E+03	2.89E+03
	rank	2	10	5	12	6	11	7	3	9	8	13	4	1
C17-F27	mean	3.09E+03	3.21E+03	3.12E+03	3.23E+03	3.10E+03	3.18E+03	3.20E+03	3.09E+03	3.12E+03	3.12E+03	3.23E+03	3.14E+03	3.16E+03
	best	3.09E+03	3.16E+03	3.10E+03	3.13E+03	3.09E+03	3.10E+03	3.18E+03	3.09E+03	3.09E+03	3.10E+03	3.21E+03	3.10E+03	3.12E+03
	worst	3.09E+03	3.28E+03	3.18E+03	3.43E+03	3.13E+03	3.22E+03	3.21E+03	3.10E+03	3.18E+03	3.17E+03	3.25E+03	3.18E+03	3.22E+03
	std	2.86E-13	5.70E+01	4.47E+01	1.44E+02	2.15E+01	5.94E+01	1.27E+01	2.71E+00	4.44E+01	4.11E+01	1.65E+01	3.98E+01	4.62E+01
	median	3.09E+03	3.20E+03	3.10E+03	3.19E+03	3.10E+03	3.20E+03	3.20E+03	3.09E+03	3.10E+03	3.10E+03	3.22E+03	3.13E+03	3.15E+03
	rank	1	11	6	13	3	9	10	2	5	4	12	7	8
C17-F28	mean	3.10E+03	3.63E+03	3.24E+03	3.78E+03	3.22E+03	3.59E+03	3.29E+03	3.24E+03	3.35E+03	3.33E+03	3.45E+03	3.31E+03	3.25E+03
	best	3.10E+03	3.58E+03	3.10E+03	3.70E+03	3.17E+03	3.41E+03	3.15E+03	3.10E+03	3.20E+03	3.21E+03	3.44E+03	3.18E+03	3.15E+03
	worst	3.10E+03	3.67E+03	3.39E+03	3.84E+03	3.24E+03	3.80E+03	3.39E+03	3.39E+03	3.41E+03	3.39E+03	3.47E+03	3.39E+03	3.52E+03
	std	0.00E+00	4.21E+01	1.41E+02	7.21E+01	3.88E+01	2.18E+02	1.34E+02	1.76E+02	1.11E+02	9.24E+01	1.61E+01	1.06E+02	1.96E+02
	median	3.10E+03	3.63E+03	3.23E+03	3.80E+03	3.23E+03	3.57E+03	3.30E+03	3.23E+03	3.39E+03	3.35E+03	3.45E+03	3.33E+03	3.16E+03
	rank	1	12	3	13	2	11	6	4	9	8	10	7	5
C17-F29	mean	3.13E+03	3.33E+03	3.29E+03	3.38E+03	3.20E+03	3.24E+03	3.35E+03	3.20E+03	3.27E+03	3.21E+03	3.35E+03	3.27E+03	3.24E+03
	best	3.13E+03	3.31E+03	3.21E+03	3.31E+03	3.17E+03	3.17E+03	3.24E+03	3.14E+03	3.19E+03	3.17E+03	3.23E+03	3.17E+03	3.19E+03
	worst	3.13E+03	3.35E+03	3.37E+03	3.45E+03	3.25E+03	3.31E+03	3.50E+03	3.29E+03	3.38E+03	3.24E+03	3.64E+03	3.35E+03	3.29E+03
	std	2.70E+00	2.02E+01	8.76E+01	7.84E+01	3.80E+01	6.30E+01	1.20E+02	6.69E+01	9.90E+01	3.59E+01	2.12E+02	9.02E+01	4.52E+01
	median	3.13E+03	3.33E+03	3.28E+03	3.38E+03	3.20E+03	3.24E+03	3.33E+03	3.19E+03	3.25E+03	3.23E+03	3.26E+03	3.28E+03	3.24E+03
	rank	1	10	9	13	3	5	12	2	7	4	11	8	6
C17-F30	mean	3.42E+03	2.23E+06	2.96E+05	3.69E+06	4.17E+05	6.17E+05	9.97E+05	3.04E+05	9.40E+05	6.09E+04	7.86E+05	3.89E+05	1.53E+06
	best	3.39E+03	1.36E+06	1.05E+05	8.31E+05	1.60E+04	1.13E+05	4.47E+03	7.46E+03	3.37E+04	2.94E+04	6.05E+05	6.41E+03	5.28E+05
	worst	3.44E+03	3.35E+06	7.71E+05	5.83E+06	6.15E+05	1.31E+06	3.76E+06	1.16E+06	1.36E+06	1.02E+05	1.00E+06	7.71E+05	3.50E+06
	std	3.02E+01	9.00E+05	3.46E+05	2.28E+06	2.96E+05	5.51E+05	2.01E+06	6.21E+05	6.78E+05	3.87E+04	1.81E+05	4.80E+05	1.52E+06
	median	3.42E+03	2.11E+06	1.54E+05	4.05E+06	5.18E+05	5.26E+05	1.10E+05	2.47E+04	1.18E+06	5.60E+04	7.68E+05	3.89E+05	1.06E+06
	rank	1	12	3	13	6	7	10	4	9	2	8	5	11
Sum rank		38	319	177	351	106	284	239	116	188	191	238	183	197
Mean rank		1.31E+00	1.10E+01	6.10E+00	1.21E+01	3.66E+00	9.79E+00	8.24E+00	4.00E+00	6.48E+00	6.59E+00	8.21E+00	6.31E+00	6.79E+00
Total rank		1	12	4	13	2	11	10	3	6	7	9	5	8

**Table 3:** Optimization results of the CEC 2017 test suite (dimensions = 30).

		TVETBO	WSO	AVOA	RSA	MPA	TSA	WOA	MVO	GWO	TLBO	GSA	PSO	GA
C17-F1	mean	1.00E+02	2.63E+10	3.11E+03	4.11E+10	2.68E+04	1.79E+10	1.70E+09	5.38E+05	1.67E+09	6.17E+09	1.05E+07	1.40E+09	1.78E+08
	best	1.00E+02	2.26E+10	2.80E+02	3.67E+10	1.23E+04	1.13E+10	1.34E+09	4.18E+05	2.75E+08	3.90E+09	2.53E+03	3.75E+03	1.33E+08
	worst	1.00E+02	3.29E+10	7.65E+03	5.06E+10	4.07E+04	2.44E+10	2.11E+09	6.84E+05	5.02E+09	9.20E+09	3.67E+07	5.61E+09	2.46E+08
	std	8.93E-15	5.20E+09	3.76E+03	6.97E+09	1.49E+04	6.69E+09	4.29E+08	1.43E+05	2.45E+09	2.41E+09	1.92E+07	3.05E+09	5.31E+07
	median	1.00E+02	2.48E+10	2.26E+03	3.86E+10	2.70E+04	1.80E+10	1.67E+09	5.24E+05	6.88E+08	5.79E+09	2.67E+06	3.20E+06	1.67E+08
	rank	1	12	2	13	3	11	9	4	8	10	5	7	6
C17-F3	mean	3.00E+02	9.73E+04	4.47E+04	7.36E+04	1.10E+03	4.72E+04	2.32E+05	1.78E+03	4.17E+04	3.47E+04	9.58E+04	3.19E+04	1.67E+05
	best	3.00E+02	8.89E+04	2.43E+04	5.70E+04	8.51E+02	4.47E+04	1.92E+05	1.39E+03	3.64E+04	2.95E+04	8.25E+04	2.27E+04	1.27E+05
	worst	3.00E+02	1.07E+05	5.78E+04	7.99E+04	1.36E+03	4.97E+04	2.66E+05	2.44E+03	4.65E+04	3.76E+04	1.06E+05	4.10E+04	2.32E+05
	std	0.00E+00	9.65E+03	1.56E+04	1.21E+04	2.47E+02	2.73E+03	3.37E+04	5.04E+02	4.52E+03	3.94E+03	1.13E+04	9.02E+03	5.46E+04
	median	3.00E+02	9.68E+04	4.84E+04	7.87E+04	1.10E+03	4.72E+04	2.35E+05	1.63E+03	4.19E+04	3.58E+04	9.76E+04	3.19E+04	1.55E+05
	rank	1	11	7	9	2	8	13	3	6	5	10	4	12
C17-F4	mean	4.59E+02	6.45E+03	5.15E+02	9.82E+03	4.93E+02	4.55E+03	8.57E+02	4.97E+02	5.72E+02	9.08E+02	5.95E+02	6.24E+02	8.12E+02
	best	4.59E+02	3.62E+03	4.92E+02	6.30E+03	4.83E+02	1.05E+03	7.92E+02	4.89E+02	5.16E+02	7.01E+02	5.74E+02	5.16E+02	7.60E+02
	worst	4.59E+02	8.73E+03	5.33E+02	1.37E+04	5.15E+02	7.56E+03	9.38E+02	5.10E+02	6.03E+02	1.31E+03	6.18E+02	8.13E+02	8.36E+02
	std	0.00E+00	2.30E+03	1.85E+01	3.36E+03	1.61E+01	2.99E+03	7.26E+01	1.03E+01	4.16E+01	2.96E+02	2.08E+01	1.48E+02	3.87E+01
	median	4.59E+02	6.72E+03	5.17E+02	9.63E+03	4.88E+02	4.79E+03	8.49E+02	4.94E+02	5.84E+02	8.11E+02	5.93E+02	5.84E+02	8.26E+02
	rank	1	12	4	13	2	11	9	3	5	10	6	7	8
C17-F5	mean	5.02E+02	8.46E+02	7.26E+02	8.85E+02	5.83E+02	7.95E+02	8.24E+02	6.20E+02	6.22E+02	7.71E+02	7.23E+02	6.33E+02	7.03E+02
	best	5.01E+02	8.25E+02	6.89E+02	8.59E+02	5.61E+02	7.66E+02	7.95E+02	6.05E+02	5.82E+02	7.48E+02	7.04E+02	6.08E+02	6.54E+02
	worst	5.04E+02	8.67E+02	7.85E+02	9.19E+02	6.06E+02	8.28E+02	8.38E+02	6.55E+02	6.51E+02	7.97E+02	7.50E+02	6.82E+02	7.66E+02
	std	1.40E+00	1.88E+01	4.72E+01	3.14E+01	2.08E+01	3.20E+01	2.13E+01	2.57E+01	3.73E+01	2.58E+01	2.22E+01	3.66E+01	5.06E+01
	median	5.02E+02	8.45E+02	7.15E+02	8.81E+02	5.83E+02	7.92E+02	8.31E+02	6.10E+02	6.28E+02	7.69E+02	7.20E+02	6.20E+02	6.96E+02
	rank	1	12	8	13	2	10	11	3	4	9	7	5	6
C17-F6	mean	6.00E+02	6.80E+02	6.47E+02	6.83E+02	6.03E+02	6.77E+02	6.76E+02	6.24E+02	6.12E+02	6.43E+02	6.56E+02	6.47E+02	6.30E+02
	best	6.00E+02	6.78E+02	6.45E+02	6.78E+02	6.02E+02	6.62E+02	6.65E+02	6.12E+02	6.05E+02	6.36E+02	6.56E+02	6.35E+02	6.23E+02
	worst	6.00E+02	6.81E+02	6.50E+02	6.90E+02	6.05E+02	6.86E+02	6.82E+02	6.37E+02	6.19E+02	6.55E+02	6.57E+02	6.58E+02	6.35E+02
	std	7.14E-14	1.18E+00	2.39E+00	6.01E+00	1.26E+00	1.24E+01	8.09E+00	1.26E+01	6.39E+00	8.94E+00	8.31E-01	1.10E+01	5.51E+00
	median	6.00E+02	6.80E+02	6.46E+02	6.82E+02	6.03E+02	6.80E+02	6.79E+02	6.24E+02	6.12E+02	6.41E+02	6.56E+02	6.48E+02	6.31E+02
	rank	1	12	7	13	2	11	10	4	3	6	9	8	5
C17-F7	mean	7.33E+02	1.30E+03	1.15E+03	1.34E+03	8.47E+02	1.22E+03	1.31E+03	8.54E+02	8.86E+02	1.08E+03	9.71E+02	8.79E+02	9.68E+02
	best	7.33E+02	1.25E+03	1.03E+03	1.33E+03	8.20E+02	1.08E+03	1.26E+03	8.01E+02	8.16E+02	9.87E+02	9.24E+02	8.58E+02	9.28E+02
	worst	7.35E+02	1.33E+03	1.31E+03	1.36E+03	9.01E+02	1.37E+03	1.39E+03	9.29E+02	9.26E+02	1.15E+03	1.04E+03	9.06E+02	1.02E+03
	std	8.20E-01	3.98E+01	1.34E+02	1.79E+01	3.98E+01	1.40E+02	6.29E+01	5.94E+01	5.27E+01	9.38E+01	5.63E+01	2.29E+01	4.30E+01
	median	7.33E+02	1.30E+03	1.12E+03	1.33E+03	8.34E+02	1.22E+03	1.29E+03	8.44E+02	9.01E+02	1.08E+03	9.60E+02	8.76E+02	9.60E+02
	rank	1	11	9	13	2	10	12	3	5	8	7	4	6
C17-F8	mean	8.03E+02	1.09E+03	9.50E+02	1.12E+03	8.91E+02	1.06E+03	1.03E+03	8.94E+02	8.93E+02	1.02E+03	9.62E+02	9.24E+02	9.87E+02
	best	8.01E+02	1.07E+03	9.20E+02	1.10E+03	8.84E+02	1.01E+03	9.74E+02	8.63E+02	8.86E+02	1.00E+03	9.37E+02	9.12E+02	9.70E+02
	worst	8.04E+02	1.11E+03	9.72E+02	1.15E+03	8.99E+02	1.16E+03	1.07E+03	9.24E+02	9.01E+02	1.06E+03	9.89E+02	9.40E+02	1.01E+03
	std	1.55E+00	1.78E+01	2.57E+01	2.64E+01	6.70E+00	7.63E+01	4.58E+01	2.89E+01	7.20E+00	2.48E+01	2.46E+01	1.35E+01	2.02E+01
	median	8.04E+02	1.08E+03	9.54E+02	1.12E+03	8.90E+02	1.03E+03	1.04E+03	8.94E+02	8.92E+02	1.02E+03	9.61E+02	9.22E+02	9.84E+02
	rank	1	12	6	13	2	11	10	4	3	9	7	5	8
C17-F9	mean	9.00E+02	1.10E+04	4.82E+03	1.06E+04	1.08E+03	1.15E+04	1.10E+04	5.45E+03	2.08E+03	5.77E+03	4.08E+03	3.55E+03	1.29E+03
	best	9.00E+02	9.37E+03	3.56E+03	1.04E+04	9.30E+02	7.00E+03	8.43E+03	4.34E+03	1.54E+03	4.17E+03	3.54E+03	2.12E+03	1.08E+03
	worst	9.00E+02	1.25E+04	5.50E+03	1.08E+04	1.24E+03	1.55E+04	1.32E+04	8.34E+03	2.87E+03	8.72E+03	4.91E+03	5.41E+03	1.50E+03
	std	7.14E-14	1.40E+03	9.41E+02	1.93E+02	1.55E+02	3.83E+03	2.58E+03	2.10E+03	7.00E+02	2.24E+03	6.55E+02	1.52E+03	2.16E+02
	median	9.00E+02	1.10E+04	5.12E+03	1.07E+04	1.09E+03	1.17E+04	1.13E+04	4.57E+03	1.95E+03	5.10E+03	3.93E+03	3.33E+03	1.29E+03
	rank	1	11	7	10	2	13	12	8	4	9	6	5	3
C17-F10	mean	2.29E+03	7.23E+03	5.46E+03	7.92E+03	4.00E+03	6.57E+03	6.51E+03	4.66E+03	4.80E+03	7.94E+03	4.86E+03	5.05E+03	6.15E+03
	best	1.85E+03	6.65E+03	4.72E+03	7.06E+03	3.64E+03	5.18E+03	5.62E+03	4.37E+03	4.28E+03	7.57E+03	4.61E+03	4.80E+03	5.67E+03
	worst	2.53E+03	7.55E+03	5.94E+03	8.55E+03	4.45E+03	7.17E+03	7.85E+03	5.04E+03	5.13E+03	8.11E+03	5.27E+03	5.51E+03	6.72E+03
	std	3.27E+02	4.34E+02	6.47E+02	6.80E+02	4.07E+02	1.02E+03	1.08E+03	3.55E+02	3.98E+02	2.70E+02	3.40E+02	3.42E+02	5.42E+02
	median	2.40E+03	7.36E+03	5.59E+03	8.03E+03	3.95E+03	6.97E+03	6.28E+03	4.61E+03	4.89E+03	8.03E+03	4.77E+03	4.94E+03	6.11E+03
	rank	1	11	7	12	2	10	9	3	4	13	5	6	8
C17-F11	mean	1.10E+03	7.52E+03	1.26E+03	8.82E+03	1.17E+03	5.14E+03	7.83E+03	1.31E+03	2.20E+03	1.99E+03	2.90E+03	1.25E+03	9.19E+03
	best	1.10E+03	6.19E+03	1.19E+03	7.18E+03	1.12E+03	3.65E+03	5.63E+03	1.27E+03	1.39E+03	1.59E+03	2.25E+03	1.22E+03	3.37E+03
	worst	1.11E+03	8.61E+03	1.32E+03	9.93E+03	1.20E+03	7.76E+03	1.16E+04	1.36E+03	4.34E+03	2.73E+03	3.58E+03	1.28E+03	1.73E+04
	std	2.34E+00	1.16E+03	5.97E+01	1.37E+03	3.83E+01	2.01E+03	2.83E+03	5.24E+01	1.56E+03	5.48E+02	6.82E+02	3.05E+01	6.48E+03
	median	1.10E+03	7.64E+03	1.26E+03	9.09E+03	1.18E+03	4.58E+03	7.05E+03	1.32E+03	1.53E+03	1.82E+03	2.89E+03	1.25E+03	8.06E+03
	rank	1	10	4	12	2	9	11	5	7	6	8	3	13
C17-F12	mean	1.74E+03	7.05E+09	2.09E+07	1.09E+10	2.17E+04	5.09E+09	2.48E+08	1.13E+07	5.27E+07	3.03E+08	2.00E+08	2.57E+06	7.71E+06
	best	1.72E+03	5.82E+09	2.94E+06	9.75E+09	1.55E+04	2.62E+09	6.35E+07	5.23E+06	5.12E+06	1.94E+08	3.86E+07	2.78E+05	5.34E+06
	worst	1.76E+03	8.95E+09	5.11E+07	1.38E+10	2.77E+04	6.65E+09	4.96E+08	2.73E+07	1.11E+08	5.26E+08	6.38E+08	5.11E+06	1.01E+07
	std	2.19E+01	1.45E+09	2.31E+07	2.08E+09	5.65E+03	1.90E+09	2.17E+08	1.16E+07	5.00E+07	1.64E+08	3.18E+08	2.27E+06	2.35E+06
	median	1.75E+03	6.71E+09	1.48E+07	1.01E+10	2.18E+04	5.54E+09	2.17E+08	6.29E+06	4.76E+07	2.46E+08	6.12E+07	2.45E+06	7.70E+06
	rank	1	12	6	13	2	11	9	5	7	10	8	3	4
C17-F13	mean	1.32E+03	5.73E+09	1.50E+05	1.06E+10	1.89E+03	1.47E+09	9.07E+05	9.12E+04	7.57E+05	8.84E+07	3.66E+04	3.25E+04	1.19E+07
	best	1.31E+03	2.79E+09	8.31E+04	5.55E+09	1.62E+03	1.98E+07	4.28E+05	3.65E+04	9.14E+04	6.14E+07	2.97E+04	1.34E+04	3.24E+06
	worst	1.32E+03	8.02E+09	2.37E+05	1.30E+10	2.43E+03	5.09E+09	1.34E+06	1.83E+05	2.35E+06	1.30E+08	5.35E+04	7.34E+04	2.57E+07
	std	2.11E+00	2.36E+09	6.97E+04	3.70E+09	4.01E+02	2.65E+09	5.18E+05	7.50E+04	1.17E+06	3.25E+07	1.24E+04	3.00E+04	1.05E+07
	median	1.31E+03	6.05E+09	1.40E+05	1.19E+10	1.76E+03	3.78E+08	9.30E+05	7.26E+04	2.94E+05	8.10E+07	3.16E+04	2.15E+04	9.43E+06
	rank	1	12	6	13	2	11	8	5	7	10	4	3	9
C17-F14	mean	1.42E+03	1.90E+06	2.72E+05	2.20E+06	1.44E+03	1.18E+06	2.23E+06	2.04E+04	5.34E+05	1.40E+05	1.15E+06	1.88E+04	2.01E+06
	best	1.42E+03	1.17E+06	3.80E+04	1.11E+06	1.44E+03	8.42E+05	3.60E+04	5.00E+03	3.44E+04	8.14E+04	7.43E+05	3.18E+03	3.33E+05
	worst	1.42E+03	2.40E+06	6.29E+05	3.28E+06	1.45E+03	1.66E+06	6.80E+06	3.47E+04	1.14E+06	1.61E+05	1.73E+06	3.43E+04	3.39E+06
	std	8.79E-01	6.28E+05	2.84E+05	1.14E+06	4.07E+00	4.09E+05	3.38E+06	1.39E+04	6.13E+05	4.26E+04	5.05E+05	1.48E+04	1.53E+06
	median	1.42E+03	2.01E+06	2.10E+05	2.21E+06	1.44E+03	1.10E+06	1.03E+06	2.09E+04	4.78E+05	1.59E+05	1.05E+06	1.88E+04	2.16E+06
	rank	1	10	6	12	2	9	13	4	7	5	8	3	11
C17-F15	mean	1.50E+03	3.04E+08	3.75E+04	5.98E+08	1.62E+03	1.44E+07	5.05E+06	4.28E+04	1.58E+07	5.14E+06	1.61E+04	4.78E+03	9.57E+05
	best	1.50E+03	2.63E+08	1.09E+04	5.16E+08	1.58E+03	5.67E+06	2.33E+05	2.48E+04	9.83E+04	1.17E+06	1.14E+04	1.91E+03	1.76E+05
	worst	1.50E+03	3.37E+08	6.09E+04	6.60E+08	1.64E+03	3.35E+07	1.64E+07	7.09E+04	5.93E+07	9.67E+06	2.18E+04	8.89E+03	2.14E+06
	std	9.31E-01	3.98E+07	2.29E+04	7.69E+07	2.75E+01	1.40E+07	8.34E+06	2.17E+04	3.15E+07	3.79E+06	4.73E+03	3.36E+03	9.80E+05
	median	1.50E+03	3.09E+08	3.91E+04	6.08E+08	1.63E+03	9.21E+06	1.79E+06	3.78E+04	1.98E+06	4.86E+06	1.55E+04	4.16E+03	7.54E+05
	rank	1	12	5	13	2	10	8	6	11	9	4	3	7
C17-F16	mean	1.66E+03	4.32E+03	3.00E+03	4.98E+03	2.03E+03	3.27E+03	4.25E+03	2.59E+03	2.55E+03	3.47E+03	3.67E+03	2.93E+03	2.95E+03
	best	1.61E+03	3.98E+03	2.56E+03	4.19E+03	1.73E+03	2.85E+03	3.49E+03	2.35E+03	2.40E+03	3.27E+03	3.48E+03	2.68E+03	2.61E+03
	worst	1.74E+03	4.60E+03	3.53E+03	5.68E+03	2.28E+03	3.53E+03	5.10E+03	2.86E+03	2.67E+03	3.70E+03	3.84E+03	3.21E+03	3.30E+03
	std	6.74E+01	3.05E+02	4.33E+02	8.65E+02	2.70E+02	3.26E+02	7.24E+02	2.39E+02	1.52E+02	2.07E+02	1.73E+02	2.90E+02	3.69E+02
	median	1.65E+03	4.35E+03	2.96E+03	5.02E+03	2.05E+03	3.36E+03	4.20E+03	2.58E+03	2.56E+03	3.44E+03	3.68E+03	2.92E+03	2.95E+03
	rank	1	12	7	13	2	8	11	4	3	9	10	5	6
C17-F17	mean	1.73E+03	3.41E+03	2.48E+03	3.72E+03	1.87E+03	3.28E+03	2.85E+03	2.08E+03	1.94E+03	2.20E+03	2.53E+03	2.34E+03	2.16E+03
	best	1.72E+03	2.81E+03	2.33E+03	3.34E+03	1.75E+03	2.22E+03	2.37E+03	2.03E+03	1.81E+03	1.97E+03	2.43E+03	2.10E+03	2.11E+03
	worst	1.73E+03	4.16E+03	2.60E+03	4.40E+03	1.93E+03	6.04E+03	3.18E+03	2.24E+03	2.09E+03	2.50E+03	2.68E+03	2.74E+03	2.23E+03
	std	7.30E+00	6.26E+02	1.25E+02	5.22E+02	8.30E+01	2.01E+03	3.76E+02	1.10E+02	1.45E+02	2.43E+02	1.34E+02	3.09E+02	5.88E+01
	median	1.73E+03	3.34E+03	2.49E+03	3.57E+03	1.89E+03	2.43E+03	2.93E+03	2.04E+03	1.93E+03	2.16E+03	2.50E+03	2.26E+03	2.15E+03
	rank	1	12	8	13	2	11	10	4	3	6	9	7	5
C17-F18	mean	1.83E+03	2.84E+07	2.65E+06	3.27E+07	1.90E+03	3.64E+07	5.91E+06	6.41E+05	4.20E+05	1.67E+06	5.15E+05	1.37E+05	3.65E+06
	best	1.82E+03	8.19E+06	2.82E+05	1.06E+07	1.87E+03	1.33E+06	1.99E+06	1.61E+05	7.85E+04	7.74E+05	2.89E+05	9.77E+04	2.85E+06
	worst	1.83E+03	5.52E+07	5.29E+06	6.43E+07	1.91E+03	6.89E+07	1.22E+07	1.73E+06	1.08E+06	2.10E+06	1.00E+06	1.63E+05	5.35E+06
	std	2.94E+00	2.26E+07	2.55E+06	2.48E+07	1.76E+01	4.08E+07	4.77E+06	7.98E+05	5.12E+05	6.61E+05	3.59E+05	3.10E+04	1.25E+06
	median	1.83E+03	2.52E+07	2.52E+06	2.80E+07	1.90E+03	3.76E+07	4.72E+06	3.33E+05	2.61E+05	1.90E+06	3.85E+05	1.44E+05	3.20E+06
	rank	1	11	8	12	2	13	10	6	4	7	5	3	9
C17-F19	mean	1.91E+03	5.81E+08	6.78E+04	9.80E+08	1.92E+03	2.95E+08	1.43E+07	9.40E+05	4.04E+06	5.76E+06	8.18E+04	4.45E+04	1.62E+06
	best	1.91E+03	4.35E+08	1.44E+04	7.08E+08	1.92E+03	3.66E+06	1.87E+06	2.37E+04	7.09E+04	2.99E+06	4.44E+04	8.77E+03	6.41E+05
	worst	1.91E+03	7.57E+08	1.51E+05	1.49E+09	1.93E+03	8.16E+08	2.48E+07	2.11E+06	1.30E+07	8.18E+06	1.10E+05	1.33E+05	2.88E+06
	std	2.10E+00	1.76E+08	6.47E+04	3.75E+08	3.69E+00	4.08E+08	1.14E+07	1.11E+06	6.56E+06	2.78E+06	2.98E+04	6.47E+04	1.03E+06
	median	1.91E+03	5.67E+08	5.28E+04	8.64E+08	1.92E+03	1.80E+08	1.54E+07	8.12E+05	1.53E+06	5.93E+06	8.64E+04	1.80E+04	1.48E+06
	rank	1	12	4	13	2	11	10	6	8	9	5	3	7
C17-F20	mean	2.07E+03	2.91E+03	2.64E+03	2.96E+03	2.18E+03	2.86E+03	2.84E+03	2.61E+03	2.38E+03	2.80E+03	3.02E+03	2.55E+03	2.48E+03
	best	2.03E+03	2.81E+03	2.48E+03	2.78E+03	2.06E+03	2.71E+03	2.64E+03	2.38E+03	2.20E+03	2.71E+03	2.64E+03	2.50E+03	2.43E+03
	worst	2.16E+03	3.02E+03	2.88E+03	3.07E+03	2.27E+03	3.01E+03	3.03E+03	3.02E+03	2.55E+03	2.93E+03	3.50E+03	2.69E+03	2.51E+03
	std	6.92E+01	9.42E+01	1.89E+02	1.38E+02	9.33E+01	1.35E+02	1.82E+02	3.06E+02	1.54E+02	1.09E+02	3.92E+02	9.73E+01	3.80E+01
	median	2.04E+03	2.89E+03	2.60E+03	2.99E+03	2.19E+03	2.85E+03	2.86E+03	2.52E+03	2.38E+03	2.79E+03	2.96E+03	2.51E+03	2.49E+03
	rank	1	11	7	12	2	10	9	6	3	8	13	5	4
C17-F21	mean	2.31E+03	2.63E+03	2.44E+03	2.68E+03	2.37E+03	2.54E+03	2.61E+03	2.41E+03	2.39E+03	2.50E+03	2.57E+03	2.44E+03	2.49E+03
	best	2.30E+03	2.53E+03	2.22E+03	2.60E+03	2.36E+03	2.31E+03	2.54E+03	2.37E+03	2.36E+03	2.49E+03	2.55E+03	2.42E+03	2.46E+03
	worst	2.31E+03	2.69E+03	2.60E+03	2.78E+03	2.38E+03	2.67E+03	2.68E+03	2.44E+03	2.41E+03	2.51E+03	2.61E+03	2.45E+03	2.55E+03
	std	4.85E+00	8.09E+01	1.76E+02	8.19E+01	1.24E+01	1.75E+02	7.75E+01	3.01E+01	2.63E+01	1.21E+01	2.67E+01	1.79E+01	3.92E+01
	median	2.31E+03	2.64E+03	2.48E+03	2.68E+03	2.36E+03	2.58E+03	2.62E+03	2.41E+03	2.40E+03	2.50E+03	2.56E+03	2.44E+03	2.49E+03
	rank	1	12	6	13	2	9	11	4	3	8	10	5	7
C17-F22	mean	2.30E+03	8.04E+03	5.80E+03	7.79E+03	2.30E+03	8.83E+03	7.45E+03	3.97E+03	2.71E+03	5.72E+03	6.36E+03	4.91E+03	2.71E+03
	best	2.30E+03	7.69E+03	2.30E+03	6.75E+03	2.30E+03	8.60E+03	6.49E+03	2.31E+03	2.58E+03	2.73E+03	4.02E+03	2.46E+03	2.63E+03
	worst	2.30E+03	8.57E+03	7.16E+03	8.84E+03	2.30E+03	8.94E+03	8.31E+03	6.03E+03	2.98E+03	9.03E+03	7.40E+03	7.28E+03	2.76E+03
	std	0.00E+00	4.08E+02	2.54E+03	9.75E+02	1.35E+00	1.76E+02	8.26E+02	2.12E+03	1.98E+02	3.73E+03	1.71E+03	2.41E+03	7.23E+01
	median	2.30E+03	7.94E+03	6.88E+03	7.79E+03	2.30E+03	8.89E+03	7.50E+03	3.77E+03	2.64E+03	5.55E+03	7.02E+03	4.95E+03	2.71E+03
	rank	1	12	8	11	2	13	10	5	4	7	9	6	3
C17-F23	mean	2.66E+03	3.20E+03	2.93E+03	3.26E+03	2.65E+03	3.20E+03	3.05E+03	2.74E+03	2.75E+03	2.91E+03	3.78E+03	2.90E+03	2.98E+03
	best	2.65E+03	3.11E+03	2.82E+03	3.20E+03	2.47E+03	3.08E+03	2.87E+03	2.69E+03	2.73E+03	2.89E+03	3.67E+03	2.87E+03	2.95E+03
	worst	2.66E+03	3.28E+03	3.11E+03	3.34E+03	2.71E+03	3.41E+03	3.15E+03	2.77E+03	2.77E+03	2.96E+03	3.89E+03	2.96E+03	3.04E+03
	std	1.80E+00	8.70E+01	1.36E+02	6.39E+01	1.29E+02	1.54E+02	1.36E+02	3.47E+01	1.92E+01	3.77E+01	1.26E+02	4.31E+01	4.72E+01
	median	2.65E+03	3.20E+03	2.90E+03	3.24E+03	2.70E+03	3.16E+03	3.09E+03	2.75E+03	2.75E+03	2.89E+03	3.78E+03	2.90E+03	2.96E+03
	rank	2	10	7	12	1	11	9	3	4	6	13	5	8
C17-F24	mean	2.83E+03	3.32E+03	3.18E+03	3.42E+03	2.88E+03	3.29E+03	3.12E+03	2.91E+03	2.92E+03	3.05E+03	3.37E+03	3.14E+03	3.23E+03
	best	2.83E+03	3.28E+03	3.04E+03	3.33E+03	2.87E+03	3.18E+03	3.06E+03	2.86E+03	2.91E+03	3.02E+03	3.34E+03	3.06E+03	3.14E+03
	worst	2.83E+03	3.40E+03	3.33E+03	3.58E+03	2.89E+03	3.34E+03	3.15E+03	2.93E+03	2.93E+03	3.08E+03	3.41E+03	3.25E+03	3.31E+03
	std	1.25E+00	5.87E+01	1.43E+02	1.25E+02	1.17E+01	8.30E+01	4.76E+01	3.60E+01	9.76E+00	2.83E+01	3.66E+01	9.01E+01	8.92E+01
	median	2.83E+03	3.30E+03	3.17E+03	3.39E+03	2.89E+03	3.32E+03	3.14E+03	2.92E+03	2.93E+03	3.04E+03	3.37E+03	3.12E+03	3.24E+03
	rank	1	11	8	13	2	10	6	3	4	5	12	7	9
C17-F25	mean	2.89E+03	3.96E+03	2.91E+03	4.59E+03	2.89E+03	3.48E+03	3.08E+03	2.91E+03	2.99E+03	3.08E+03	3.00E+03	2.90E+03	3.11E+03
	best	2.89E+03	3.57E+03	2.89E+03	3.98E+03	2.88E+03	3.09E+03	3.05E+03	2.88E+03	2.96E+03	2.95E+03	2.99E+03	2.89E+03	3.09E+03
	worst	2.89E+03	4.24E+03	2.95E+03	5.41E+03	2.90E+03	3.88E+03	3.10E+03	2.98E+03	3.07E+03	3.22E+03	3.01E+03	2.91E+03	3.12E+03
	std	8.27E-03	3.04E+02	2.88E+01	6.49E+02	6.46E+00	4.16E+02	2.89E+01	4.74E+01	5.58E+01	1.36E+02	1.10E+01	1.28E+01	1.41E+01
	median	2.89E+03	4.00E+03	2.90E+03	4.49E+03	2.89E+03	3.47E+03	3.09E+03	2.89E+03	2.98E+03	3.07E+03	3.00E+03	2.89E+03	3.11E+03
	rank	1	12	4	13	2	11	9	5	6	8	7	3	10
C17-F26	mean	3.58E+03	9.25E+03	7.38E+03	9.85E+03	2.94E+03	8.80E+03	8.44E+03	4.80E+03	4.58E+03	5.96E+03	7.53E+03	4.87E+03	4.40E+03
	best	3.56E+03	8.82E+03	6.07E+03	9.00E+03	2.94E+03	8.13E+03	7.70E+03	4.45E+03	4.18E+03	4.55E+03	6.46E+03	3.54E+03	4.01E+03
	worst	3.61E+03	1.00E+04	8.15E+03	1.14E+04	2.94E+03	9.23E+03	9.31E+03	5.44E+03	5.20E+03	7.29E+03	8.08E+03	6.43E+03	4.88E+03
	std	2.48E+01	6.15E+02	9.92E+02	1.20E+03	1.69E+00	5.11E+02	7.20E+02	5.02E+02	4.74E+02	1.36E+03	8.23E+02	1.47E+03	3.97E+02
	median	3.57E+03	9.08E+03	7.65E+03	9.52E+03	2.94E+03	8.93E+03	8.38E+03	4.66E+03	4.47E+03	5.99E+03	7.78E+03	4.74E+03	4.36E+03
	rank	2	12	8	13	1	11	10	5	4	7	9	6	3
C17-F27	mean	3.21E+03	3.62E+03	3.36E+03	3.77E+03	3.21E+03	3.48E+03	3.43E+03	3.23E+03	3.25E+03	3.32E+03	5.00E+03	3.28E+03	3.46E+03
	best	3.20E+03	3.56E+03	3.27E+03	3.49E+03	3.20E+03	3.34E+03	3.26E+03	3.21E+03	3.24E+03	3.24E+03	4.54E+03	3.24E+03	3.39E+03
	worst	3.21E+03	3.72E+03	3.43E+03	4.07E+03	3.24E+03	3.73E+03	3.56E+03	3.26E+03	3.27E+03	3.39E+03	5.33E+03	3.32E+03	3.51E+03
	std	5.06E+00	7.90E+01	9.45E+01	2.69E+02	1.74E+01	1.89E+02	1.40E+02	2.10E+01	1.14E+01	6.84E+01	4.22E+02	3.88E+01	5.92E+01
	median	3.21E+03	3.60E+03	3.36E+03	3.77E+03	3.21E+03	3.42E+03	3.45E+03	3.23E+03	3.25E+03	3.32E+03	5.06E+03	3.28E+03	3.48E+03
	rank	1	11	7	12	2	10	8	3	4	6	13	5	9
C17-F28	mean	3.10E+03	4.81E+03	3.27E+03	5.73E+03	3.22E+03	4.18E+03	3.44E+03	3.26E+03	3.61E+03	3.68E+03	3.53E+03	3.33E+03	3.59E+03
	best	3.10E+03	4.57E+03	3.24E+03	5.42E+03	3.20E+03	3.61E+03	3.38E+03	3.22E+03	3.40E+03	3.53E+03	3.46E+03	3.20E+03	3.54E+03
	worst	3.10E+03	5.07E+03	3.30E+03	6.06E+03	3.25E+03	4.75E+03	3.50E+03	3.29E+03	4.10E+03	4.03E+03	3.68E+03	3.54E+03	3.65E+03
	std	2.86E-13	2.33E+02	2.85E+01	3.35E+02	2.32E+01	5.78E+02	5.60E+01	3.15E+01	3.62E+02	2.57E+02	1.12E+02	1.75E+02	5.72E+01
	median	3.10E+03	4.80E+03	3.27E+03	5.72E+03	3.21E+03	4.17E+03	3.45E+03	3.26E+03	3.46E+03	3.58E+03	3.49E+03	3.30E+03	3.59E+03
	rank	1	12	4	13	2	11	6	3	9	10	7	5	8
C17-F29	mean	3.35E+03	5.43E+03	4.35E+03	5.66E+03	3.66E+03	5.27E+03	5.12E+03	3.85E+03	3.80E+03	4.53E+03	5.09E+03	4.18E+03	4.30E+03
	best	3.33E+03	4.97E+03	3.99E+03	5.01E+03	3.51E+03	4.72E+03	4.84E+03	3.72E+03	3.71E+03	4.20E+03	4.81E+03	3.98E+03	3.91E+03
	worst	3.37E+03	5.92E+03	4.57E+03	6.54E+03	3.80E+03	6.17E+03	5.29E+03	3.97E+03	3.92E+03	5.02E+03	5.35E+03	4.43E+03	4.67E+03
	std	2.14E+01	4.96E+02	2.78E+02	8.16E+02	1.43E+02	7.41E+02	2.10E+02	1.16E+02	1.01E+02	3.84E+02	3.15E+02	2.03E+02	3.66E+02
	median	3.36E+03	5.42E+03	4.42E+03	5.54E+03	3.67E+03	5.10E+03	5.17E+03	3.85E+03	3.78E+03	4.45E+03	5.10E+03	4.16E+03	4.32E+03
	rank	1	12	7	13	2	11	10	4	3	8	9	5	6
C17-F30	mean	5.01E+03	1.44E+09	1.44E+06	2.84E+09	7.70E+03	3.87E+07	3.95E+07	3.11E+06	6.42E+06	3.81E+07	2.28E+06	2.74E+05	7.07E+05
	best	4.96E+03	1.06E+09	5.06E+05	2.04E+09	6.38E+03	1.32E+07	7.87E+06	5.59E+05	1.43E+06	2.04E+07	1.99E+06	7.61E+03	1.95E+05
	worst	5.09E+03	1.58E+09	2.54E+06	3.14E+09	1.03E+04	9.04E+07	6.32E+07	4.46E+06	1.73E+07	7.99E+07	2.74E+06	1.04E+06	1.35E+06
	std	6.42E+01	2.76E+08	9.26E+05	5.82E+08	1.98E+03	3.81E+07	2.51E+07	1.89E+06	7.99E+06	3.05E+07	3.52E+05	5.54E+05	6.12E+05
	median	4.99E+03	1.56E+09	1.35E+06	3.09E+09	7.08E+03	2.56E+07	4.34E+07	3.72E+06	3.46E+06	2.60E+07	2.19E+06	2.55E+04	6.40E+05
	rank	1	12	5	13	2	10	11	7	8	9	6	3	4
Sum rank		31	334	182	361	57	305	284	128	151	232	231	139	204
Mean rank		1.07E+00	1.15E+01	6.28E+00	1.24E+01	1.97E+00	1.05E+01	9.79E+00	4.41E+00	5.21E+00	8.00E+00	7.97E+00	4.79E+00	7.03E+00
Total rank		1	12	6	13	2	11	10	3	5	9	8	4	7

**Table 4:** Optimization results of the CEC 2017 test suite (dimensions = 50).

		TVETBO	WSO	AVOA	RSA	MPA	TSA	WOA	MVO	GWO	TLBO	GSA	PSO	GA
C17-F1	mean	1.00E+02	5.97E+10	9.22E+06	9.35E+10	5.62E+06	3.80E+10	7.68E+09	4.06E+06	9.33E+09	2.07E+10	1.71E+10	2.53E+09	1.04E+10
	best	1.00E+02	5.33E+10	1.10E+06	8.17E+10	2.17E+06	3.50E+10	4.53E+09	2.90E+06	6.73E+09	1.41E+10	1.36E+10	1.04E+09	9.88E+09
	worst	1.00E+02	6.39E+10	2.44E+07	1.02E+11	1.42E+07	4.09E+10	1.15E+10	5.05E+06	1.28E+10	2.79E+10	2.04E+10	3.37E+09	1.12E+10
	std	0.00E+00	5.09E+09	1.13E+07	9.68E+09	6.30E+06	2.66E+09	3.58E+09	9.61E+05	2.74E+09	7.31E+09	3.03E+09	1.12E+09	6.62E+08
	median	1.00E+02	6.08E+10	5.69E+06	9.50E+10	3.03E+06	3.81E+10	7.35E+09	4.14E+06	8.91E+09	2.04E+10	1.72E+10	2.85E+09	1.02E+10
	rank	1	12	4	13	3	11	6	2	7	10	9	5	8
C17-F3	mean	3.00E+02	1.58E+05	1.46E+05	1.57E+05	1.79E+04	1.09E+05	2.33E+05	4.61E+04	1.30E+05	9.80E+04	1.77E+05	1.44E+05	2.63E+05
	best	3.00E+02	1.36E+05	1.12E+05	1.43E+05	1.54E+04	9.56E+04	1.76E+05	3.66E+04	1.14E+05	7.41E+04	1.60E+05	1.08E+05	2.19E+05
	worst	3.00E+02	1.82E+05	1.78E+05	1.72E+05	2.11E+04	1.16E+05	3.56E+05	5.74E+04	1.45E+05	1.12E+05	2.00E+05	1.88E+05	3.02E+05
	std	0.00E+00	2.12E+04	3.22E+04	1.39E+04	2.76E+03	1.03E+04	9.22E+04	9.43E+03	1.41E+04	1.87E+04	2.12E+04	3.76E+04	3.69E+04
	median	3.00E+02	1.57E+05	1.47E+05	1.58E+05	1.75E+04	1.12E+05	2.01E+05	4.53E+04	1.30E+05	1.03E+05	1.75E+05	1.40E+05	2.65E+05
	rank	1	10	8	9	2	5	12	3	6	4	11	7	13
C17-F4	mean	4.70E+02	1.47E+04	6.97E+02	2.37E+04	5.31E+02	8.29E+03	1.94E+03	5.63E+02	1.43E+03	2.79E+03	3.06E+03	1.01E+03	1.52E+03
	best	4.29E+02	1.14E+04	6.81E+02	1.56E+04	4.96E+02	6.64E+03	1.22E+03	5.26E+02	1.07E+03	1.58E+03	2.55E+03	6.83E+02	1.32E+03
	worst	5.26E+02	1.68E+04	7.25E+02	2.83E+04	5.83E+02	1.07E+04	2.32E+03	6.41E+02	1.76E+03	4.77E+03	3.25E+03	1.81E+03	1.65E+03
	std	5.39E+01	2.59E+03	2.19E+01	6.29E+03	4.44E+01	1.87E+03	5.33E+02	5.83E+01	3.37E+02	1.53E+03	3.67E+02	5.81E+02	1.59E+02
	median	4.64E+02	1.53E+04	6.91E+02	2.54E+04	5.22E+02	7.90E+03	2.10E+03	5.42E+02	1.45E+03	2.40E+03	3.21E+03	7.81E+02	1.56E+03
	rank	1	12	4	13	2	11	8	3	6	9	10	5	7
C17-F5	mean	5.05E+02	1.10E+03	8.56E+02	1.13E+03	7.35E+02	1.14E+03	9.54E+02	7.38E+02	7.24E+02	9.97E+02	8.05E+02	7.88E+02	8.90E+02
	best	5.04E+02	1.06E+03	8.26E+02	1.11E+03	6.54E+02	1.00E+03	9.13E+02	6.64E+02	6.97E+02	9.55E+02	7.52E+02	7.33E+02	8.60E+02
	worst	5.06E+02	1.14E+03	8.97E+02	1.14E+03	7.99E+02	1.26E+03	9.79E+02	8.50E+02	7.52E+02	1.02E+03	8.41E+02	8.52E+02	9.11E+02
	std	1.04E+00	3.81E+01	3.36E+01	1.60E+01	6.61E+01	1.35E+02	3.17E+01	9.04E+01	3.23E+01	3.36E+01	4.55E+01	5.30E+01	2.63E+01
	median	5.04E+02	1.09E+03	8.51E+02	1.13E+03	7.44E+02	1.16E+03	9.62E+02	7.18E+02	7.24E+02	1.00E+03	8.13E+02	7.83E+02	8.94E+02
	rank	1	11	7	12	3	13	9	4	2	10	6	5	8
C17-F6	mean	6.00E+02	6.94E+02	6.60E+02	6.96E+02	6.11E+02	6.89E+02	6.97E+02	6.37E+02	6.23E+02	6.64E+02	6.57E+02	6.53E+02	6.48E+02
	best	6.00E+02	6.91E+02	6.55E+02	6.94E+02	6.08E+02	6.69E+02	6.92E+02	6.27E+02	6.17E+02	6.51E+02	6.53E+02	6.51E+02	6.35E+02
	worst	6.00E+02	6.99E+02	6.65E+02	6.99E+02	6.15E+02	7.06E+02	7.05E+02	6.61E+02	6.32E+02	6.72E+02	6.60E+02	6.57E+02	6.61E+02
	std	0.00E+00	3.97E+00	5.12E+00	2.63E+00	2.99E+00	1.78E+01	6.37E+00	1.76E+01	7.53E+00	9.88E+00	3.72E+00	2.85E+00	1.15E+01
	median	6.00E+02	6.93E+02	6.59E+02	6.96E+02	6.11E+02	6.91E+02	6.96E+02	6.31E+02	6.21E+02	6.66E+02	6.58E+02	6.53E+02	6.48E+02
	rank	1	11	8	12	2	10	13	4	3	9	7	6	5
C17-F7	mean	7.57E+02	1.79E+03	1.66E+03	1.89E+03	1.03E+03	1.68E+03	1.70E+03	1.05E+03	1.06E+03	1.47E+03	1.41E+03	1.20E+03	1.30E+03
	best	7.55E+02	1.76E+03	1.59E+03	1.80E+03	9.71E+02	1.53E+03	1.64E+03	1.01E+03	1.04E+03	1.35E+03	1.24E+03	1.04E+03	1.23E+03
	worst	7.58E+02	1.82E+03	1.73E+03	1.99E+03	1.07E+03	1.83E+03	1.79E+03	1.08E+03	1.08E+03	1.54E+03	1.54E+03	1.43E+03	1.35E+03
	std	1.69E+00	2.57E+01	6.32E+01	8.56E+01	5.50E+01	1.52E+02	7.55E+01	3.13E+01	2.13E+01	9.30E+01	1.45E+02	1.83E+02	6.19E+01
	median	7.57E+02	1.78E+03	1.66E+03	1.87E+03	1.03E+03	1.68E+03	1.69E+03	1.06E+03	1.07E+03	1.51E+03	1.43E+03	1.16E+03	1.32E+03
	rank	1	12	9	13	2	10	11	3	4	8	7	5	6
C17-F8	mean	8.06E+02	1.42E+03	1.12E+03	1.44E+03	1.01E+03	1.43E+03	1.32E+03	1.02E+03	1.03E+03	1.32E+03	1.14E+03	1.06E+03	1.26E+03
	best	8.03E+02	1.36E+03	1.08E+03	1.41E+03	9.79E+02	1.33E+03	1.19E+03	9.81E+02	9.98E+02	1.26E+03	1.13E+03	1.01E+03	1.21E+03
	worst	8.11E+02	1.46E+03	1.17E+03	1.47E+03	1.04E+03	1.57E+03	1.43E+03	1.09E+03	1.07E+03	1.37E+03	1.15E+03	1.12E+03	1.28E+03
	std	3.89E+00	4.92E+01	5.75E+01	2.45E+01	3.51E+01	1.09E+02	1.09E+02	5.28E+01	3.53E+01	5.03E+01	1.11E+01	5.58E+01	3.13E+01
	median	8.04E+02	1.42E+03	1.12E+03	1.45E+03	1.01E+03	1.42E+03	1.33E+03	1.01E+03	1.03E+03	1.32E+03	1.14E+03	1.04E+03	1.26E+03
	rank	1	11	6	13	2	12	10	3	4	9	7	5	8
C17-F9	mean	9.00E+02	3.59E+04	1.32E+04	3.61E+04	3.31E+03	3.76E+04	3.28E+04	1.95E+04	6.83E+03	2.39E+04	1.06E+04	1.02E+04	1.27E+04
	best	9.00E+02	3.45E+04	1.26E+04	3.39E+04	2.06E+03	3.47E+04	3.05E+04	1.04E+04	5.93E+03	1.84E+04	9.65E+03	9.46E+03	1.05E+04
	worst	9.00E+02	3.92E+04	1.40E+04	3.79E+04	4.79E+03	4.20E+04	3.83E+04	2.58E+04	7.77E+03	2.81E+04	1.14E+04	1.16E+04	1.46E+04
	std	1.01E-13	2.44E+03	6.96E+02	2.04E+03	1.23E+03	3.42E+03	4.05E+03	7.86E+03	1.04E+03	4.38E+03	8.09E+02	1.05E+03	2.41E+03
	median	9.00E+02	3.49E+04	1.31E+04	3.63E+04	3.18E+03	3.69E+04	3.11E+04	2.09E+04	6.80E+03	2.45E+04	1.06E+04	9.89E+03	1.29E+04
	rank	1	11	7	12	2	13	10	8	3	9	5	4	6
C17-F10	mean	4.35E+03	1.30E+04	8.32E+03	1.42E+04	6.54E+03	1.17E+04	1.18E+04	7.66E+03	8.66E+03	1.40E+04	8.59E+03	7.79E+03	1.17E+04
	best	3.56E+03	1.25E+04	7.78E+03	1.39E+04	5.64E+03	1.08E+04	1.05E+04	6.35E+03	6.60E+03	1.33E+04	7.74E+03	7.58E+03	1.11E+04
	worst	5.10E+03	1.37E+04	8.86E+03	1.46E+04	7.15E+03	1.28E+04	1.29E+04	8.74E+03	1.38E+04	1.45E+04	9.68E+03	8.32E+03	1.24E+04
	std	7.01E+02	6.53E+02	4.91E+02	3.33E+02	7.98E+02	9.48E+02	1.14E+03	1.12E+03	3.77E+03	7.27E+02	8.79E+02	3.86E+02	6.12E+02
	median	4.37E+03	1.28E+04	8.32E+03	1.41E+04	6.68E+03	1.17E+04	1.18E+04	7.77E+03	7.10E+03	1.40E+04	8.47E+03	7.62E+03	1.16E+04
	rank	1	11	5	13	2	9	10	3	7	12	6	4	8
C17-F11	mean	1.13E+03	1.53E+04	1.60E+03	2.09E+04	1.25E+03	1.29E+04	5.08E+03	1.57E+03	6.11E+03	5.10E+03	1.41E+04	1.67E+03	2.39E+04
	best	1.12E+03	1.41E+04	1.48E+03	1.86E+04	1.21E+03	1.11E+04	4.48E+03	1.42E+03	3.67E+03	4.78E+03	1.33E+04	1.40E+03	1.40E+04
	worst	1.13E+03	1.61E+04	1.76E+03	2.27E+04	1.29E+03	1.55E+04	6.36E+03	1.72E+03	1.06E+04	5.67E+03	1.60E+04	2.00E+03	3.21E+04
	std	5.92E+00	9.49E+02	1.37E+02	1.85E+03	3.83E+01	2.06E+03	9.41E+02	1.44E+02	3.49E+03	4.48E+02	1.38E+03	2.78E+02	8.14E+03
	median	1.13E+03	1.56E+04	1.59E+03	2.12E+04	1.26E+03	1.25E+04	4.74E+03	1.57E+03	5.08E+03	4.97E+03	1.36E+04	1.64E+03	2.48E+04
	rank	1	11	4	12	2	9	6	3	8	7	10	5	13
C17-F12	mean	2.91E+03	4.35E+10	7.32E+07	7.10E+10	1.44E+07	2.58E+10	1.32E+09	7.91E+07	9.56E+08	5.04E+09	2.17E+09	1.60E+09	2.04E+08
	best	2.53E+03	3.66E+10	3.10E+07	5.18E+10	1.35E+07	1.09E+10	1.09E+09	4.26E+07	1.50E+08	2.84E+09	7.13E+08	1.27E+07	6.44E+07
	worst	3.17E+03	5.22E+10	1.13E+08	9.74E+10	1.50E+07	4.34E+10	1.80E+09	1.26E+08	1.78E+09	9.92E+09	3.89E+09	4.63E+09	2.82E+08
	std	2.98E+02	7.68E+09	4.79E+07	2.28E+10	7.66E+05	1.46E+10	3.53E+08	3.81E+07	8.82E+08	3.61E+09	1.43E+09	2.34E+09	1.04E+08
	median	2.96E+03	4.27E+10	7.44E+07	6.75E+10	1.45E+07	2.45E+10	1.19E+09	7.39E+07	9.49E+08	3.70E+09	2.03E+09	8.82E+08	2.35E+08
	rank	1	12	3	13	2	11	7	4	6	10	9	8	5
C17-F13	mean	1.34E+03	2.45E+10	1.49E+05	4.30E+10	1.63E+04	1.01E+10	9.47E+07	2.41E+05	3.57E+08	5.84E+08	1.85E+07	4.76E+08	4.14E+07
	best	1.33E+03	1.42E+10	3.42E+04	2.17E+10	8.63E+03	5.35E+09	7.12E+07	1.50E+05	1.62E+08	4.76E+08	3.12E+04	5.09E+04	2.70E+07
	worst	1.34E+03	3.35E+10	3.28E+05	6.19E+10	1.92E+04	1.57E+10	1.08E+08	3.76E+05	8.97E+08	7.98E+08	6.23E+07	1.20E+09	5.54E+07
	std	4.66E+00	9.23E+09	1.37E+05	1.83E+10	5.57E+03	4.75E+09	1.75E+07	1.04E+05	3.92E+08	1.58E+08	3.23E+07	6.38E+08	1.38E+07
	median	1.34E+03	2.53E+10	1.17E+05	4.42E+10	1.87E+04	9.62E+09	1.00E+08	2.19E+05	1.84E+08	5.31E+08	5.80E+06	3.51E+08	4.17E+07
	rank	1	12	3	13	2	11	7	4	8	10	5	9	6
C17-F14	mean	1.43E+03	2.59E+07	1.22E+06	4.83E+07	1.56E+03	2.68E+06	4.76E+06	1.91E+05	1.15E+06	8.65E+05	1.51E+07	5.73E+05	1.12E+07
	best	1.43E+03	8.47E+06	3.78E+05	1.48E+07	1.55E+03	7.09E+05	4.22E+06	1.21E+05	8.96E+04	7.13E+05	3.43E+06	2.06E+05	5.51E+06
	worst	1.43E+03	5.08E+07	2.91E+06	9.79E+07	1.59E+03	4.26E+06	5.66E+06	3.70E+05	2.22E+06	9.97E+05	2.48E+07	9.18E+05	1.93E+07
	std	2.85E+00	1.94E+07	1.25E+06	3.84E+07	1.88E+01	1.60E+06	6.78E+05	1.31E+05	9.46E+05	1.62E+05	1.06E+07	3.17E+05	6.32E+06
	median	1.43E+03	2.22E+07	7.99E+05	4.03E+07	1.56E+03	2.88E+06	4.59E+06	1.36E+05	1.15E+06	8.74E+05	1.61E+07	5.85E+05	1.00E+07
	rank	1	12	7	13	2	8	9	3	6	5	11	4	10
C17-F15	mean	1.53E+03	2.60E+09	3.80E+04	4.18E+09	2.26E+03	1.70E+09	9.91E+06	1.21E+05	5.94E+06	7.05E+07	1.97E+08	1.08E+04	8.57E+06
	best	1.53E+03	1.84E+09	2.34E+04	3.27E+09	2.13E+03	5.85E+08	9.14E+05	5.02E+04	4.23E+04	4.13E+07	1.91E+04	2.77E+03	2.91E+06
	worst	1.53E+03	3.41E+09	6.99E+04	4.95E+09	2.41E+03	3.71E+09	1.85E+07	1.81E+05	1.57E+07	9.18E+07	7.65E+08	2.13E+04	1.86E+07
	std	3.19E+00	8.02E+08	2.34E+04	8.14E+08	1.61E+02	1.58E+09	8.41E+06	6.32E+04	7.41E+06	2.29E+07	4.12E+08	8.96E+03	7.55E+06
	median	1.53E+03	2.58E+09	2.93E+04	4.25E+09	2.25E+03	1.26E+09	1.01E+07	1.27E+05	4.04E+06	7.44E+07	1.19E+07	9.65E+03	6.38E+06
	rank	1	12	4	13	2	11	8	5	6	9	10	3	7
C17-F16	mean	2.06E+03	6.28E+03	4.35E+03	7.58E+03	2.75E+03	4.64E+03	5.49E+03	3.33E+03	3.32E+03	4.54E+03	3.95E+03	3.34E+03	3.91E+03
	best	1.73E+03	5.46E+03	4.03E+03	5.67E+03	2.59E+03	4.08E+03	4.49E+03	3.09E+03	2.92E+03	4.13E+03	3.61E+03	2.92E+03	3.30E+03
	worst	2.24E+03	8.03E+03	4.77E+03	1.13E+04	3.02E+03	4.94E+03	6.15E+03	3.57E+03	3.91E+03	4.85E+03	4.36E+03	3.79E+03	4.44E+03
	std	2.53E+02	1.32E+03	3.83E+02	2.81E+03	2.18E+02	4.24E+02	7.97E+02	2.17E+02	5.21E+02	3.30E+02	3.90E+02	4.67E+02	5.38E+02
	median	2.14E+03	5.81E+03	4.31E+03	6.66E+03	2.70E+03	4.77E+03	5.66E+03	3.33E+03	3.23E+03	4.60E+03	3.93E+03	3.32E+03	3.95E+03
	rank	1	12	8	13	2	10	11	4	3	9	7	5	6
C17-F17	mean	2.02E+03	7.62E+03	3.56E+03	1.10E+04	2.56E+03	3.96E+03	4.53E+03	3.07E+03	2.97E+03	4.15E+03	3.82E+03	3.35E+03	3.58E+03
	best	1.90E+03	5.80E+03	3.11E+03	8.06E+03	2.49E+03	3.17E+03	4.06E+03	2.52E+03	2.81E+03	3.51E+03	3.37E+03	3.12E+03	3.35E+03
	worst	2.14E+03	9.34E+03	4.08E+03	1.44E+04	2.61E+03	4.41E+03	4.75E+03	3.57E+03	3.24E+03	4.53E+03	4.12E+03	3.68E+03	3.83E+03
	std	1.46E+02	1.59E+03	5.05E+02	2.82E+03	5.63E+01	5.89E+02	3.52E+02	4.70E+02	2.05E+02	4.89E+02	3.60E+02	2.87E+02	2.46E+02
	median	2.02E+03	7.66E+03	3.52E+03	1.09E+04	2.57E+03	4.12E+03	4.66E+03	3.10E+03	2.91E+03	4.28E+03	3.89E+03	3.30E+03	3.57E+03
	rank	1	12	6	13	2	9	11	4	3	10	8	5	7
C17-F18	mean	1.83E+03	7.57E+07	2.41E+06	1.12E+08	2.62E+04	3.50E+07	4.52E+07	2.64E+06	5.72E+06	8.20E+06	8.41E+06	8.24E+05	9.47E+06
	best	1.82E+03	6.06E+07	3.12E+05	5.05E+07	3.74E+03	3.15E+06	1.22E+07	1.56E+06	1.09E+06	5.64E+06	3.97E+06	3.51E+05	3.39E+06
	worst	1.84E+03	8.92E+07	4.41E+06	1.56E+08	3.92E+04	1.00E+08	8.18E+07	4.11E+06	1.14E+07	1.14E+07	1.57E+07	1.35E+06	2.28E+07
	std	8.86E+00	1.35E+07	2.26E+06	5.63E+07	1.69E+04	4.85E+07	3.74E+07	1.33E+06	5.86E+06	2.65E+06	5.82E+06	4.99E+05	9.73E+06
	median	1.83E+03	7.65E+07	2.46E+06	1.21E+08	3.10E+04	1.85E+07	4.33E+07	2.45E+06	5.19E+06	7.88E+06	6.97E+06	7.97E+05	5.86E+06
	rank	1	12	4	13	2	10	11	5	6	7	8	3	9
C17-F19	mean	1.93E+03	2.72E+09	2.60E+05	3.84E+09	2.08E+03	2.67E+09	6.84E+06	5.12E+06	1.16E+06	5.07E+07	4.52E+05	3.94E+05	9.91E+05
	best	1.92E+03	1.30E+09	9.11E+04	2.59E+09	2.02E+03	9.78E+06	1.03E+06	3.90E+06	5.69E+05	4.30E+07	2.60E+05	2.90E+03	7.76E+05
	worst	1.93E+03	4.54E+09	5.35E+05	4.75E+09	2.11E+03	7.81E+09	1.61E+07	6.35E+06	1.79E+06	6.44E+07	9.90E+05	9.83E+05	1.34E+06
	std	8.61E-01	1.49E+09	2.10E+05	1.04E+09	4.55E+01	3.80E+09	7.06E+06	1.09E+06	5.54E+05	1.03E+07	3.91E+05	5.08E+05	2.91E+05
	median	1.93E+03	2.52E+09	2.06E+05	4.01E+09	2.10E+03	1.44E+09	5.10E+06	5.12E+06	1.15E+06	4.77E+07	2.79E+05	2.94E+05	9.24E+05
	rank	1	12	3	13	2	11	9	8	7	10	5	4	6
C17-F20	mean	2.16E+03	3.83E+03	3.27E+03	4.10E+03	2.66E+03	3.43E+03	3.75E+03	3.28E+03	2.62E+03	3.78E+03	4.04E+03	3.29E+03	3.17E+03
	best	2.10E+03	3.49E+03	2.67E+03	3.82E+03	2.38E+03	2.98E+03	3.45E+03	3.04E+03	2.42E+03	3.65E+03	3.75E+03	2.88E+03	3.10E+03
	worst	2.32E+03	4.01E+03	3.80E+03	4.26E+03	2.94E+03	3.65E+03	4.34E+03	3.75E+03	2.83E+03	3.95E+03	4.32E+03	3.47E+03	3.30E+03
	std	1.19E+02	2.55E+02	5.29E+02	2.13E+02	2.60E+02	3.34E+02	4.41E+02	3.52E+02	2.33E+02	1.40E+02	2.55E+02	2.98E+02	9.63E+01
	median	2.11E+03	3.91E+03	3.30E+03	4.15E+03	2.66E+03	3.56E+03	3.61E+03	3.16E+03	2.62E+03	3.75E+03	4.05E+03	3.40E+03	3.14E+03
	rank	1	11	5	13	3	8	9	6	2	10	12	7	4
C17-F21	mean	2.31E+03	2.99E+03	2.76E+03	3.03E+03	2.45E+03	2.96E+03	2.95E+03	2.57E+03	2.52E+03	2.82E+03	2.84E+03	2.66E+03	2.75E+03
	best	2.31E+03	2.96E+03	2.63E+03	2.93E+03	2.43E+03	2.85E+03	2.84E+03	2.54E+03	2.47E+03	2.80E+03	2.77E+03	2.59E+03	2.73E+03
	worst	2.33E+03	3.03E+03	2.95E+03	3.12E+03	2.47E+03	3.13E+03	3.05E+03	2.61E+03	2.56E+03	2.87E+03	2.88E+03	2.77E+03	2.77E+03
	std	1.08E+01	3.88E+01	1.46E+02	9.96E+01	2.51E+01	1.30E+02	9.84E+01	4.16E+01	4.53E+01	3.51E+01	5.45E+01	8.62E+01	2.27E+01
	median	2.31E+03	3.00E+03	2.72E+03	3.04E+03	2.45E+03	2.93E+03	2.96E+03	2.57E+03	2.53E+03	2.81E+03	2.86E+03	2.64E+03	2.75E+03
	rank	1	12	7	13	2	11	10	4	3	8	9	5	6
C17-F22	mean	3.10E+03	1.50E+04	1.12E+04	1.63E+04	5.36E+03	1.38E+04	1.37E+04	9.01E+03	8.89E+03	1.57E+04	1.15E+04	9.77E+03	8.85E+03
	best	2.30E+03	1.47E+04	8.83E+03	1.61E+04	2.32E+03	1.33E+04	1.31E+04	6.98E+03	7.84E+03	1.53E+04	1.12E+04	8.96E+03	4.03E+03
	worst	5.48E+03	1.52E+04	1.29E+04	1.65E+04	8.56E+03	1.44E+04	1.41E+04	1.03E+04	9.31E+03	1.63E+04	1.18E+04	1.03E+04	1.36E+04
	std	1.73E+03	2.17E+02	2.07E+03	2.49E+02	3.70E+03	4.87E+02	4.60E+02	1.56E+03	7.69E+02	6.06E+02	2.93E+02	6.42E+02	5.73E+03
	median	2.30E+03	1.51E+04	1.15E+04	1.63E+04	5.28E+03	1.37E+04	1.38E+04	9.37E+03	9.20E+03	1.57E+04	1.14E+04	9.92E+03	8.87E+03
	rank	1	11	7	13	2	10	9	5	4	12	8	6	3
C17-F23	mean	2.74E+03	3.83E+03	3.30E+03	3.91E+03	2.89E+03	3.75E+03	3.76E+03	2.99E+03	3.02E+03	3.29E+03	4.78E+03	3.38E+03	3.37E+03
	best	2.73E+03	3.75E+03	3.21E+03	3.86E+03	2.88E+03	3.54E+03	3.57E+03	2.95E+03	2.94E+03	3.20E+03	4.58E+03	3.32E+03	3.24E+03
	worst	2.75E+03	3.93E+03	3.38E+03	3.95E+03	2.91E+03	4.10E+03	3.86E+03	3.07E+03	3.16E+03	3.36E+03	4.95E+03	3.44E+03	3.51E+03
	std	1.09E+01	8.51E+01	8.65E+01	4.13E+01	1.57E+01	2.88E+02	1.40E+02	6.04E+01	1.06E+02	7.21E+01	1.66E+02	7.32E+01	1.21E+02
	median	2.75E+03	3.82E+03	3.30E+03	3.91E+03	2.89E+03	3.69E+03	3.80E+03	2.98E+03	2.99E+03	3.30E+03	4.79E+03	3.39E+03	3.36E+03
	rank	1	11	6	12	2	9	10	3	4	5	13	8	7
C17-F24	mean	2.92E+03	4.23E+03	3.52E+03	4.51E+03	3.07E+03	4.02E+03	3.84E+03	3.14E+03	3.20E+03	3.46E+03	4.40E+03	3.47E+03	3.67E+03
	best	2.91E+03	3.97E+03	3.41E+03	4.01E+03	3.04E+03	3.92E+03	3.73E+03	3.10E+03	3.10E+03	3.38E+03	4.37E+03	3.31E+03	3.63E+03
	worst	2.92E+03	4.81E+03	3.71E+03	5.72E+03	3.11E+03	4.16E+03	3.90E+03	3.17E+03	3.33E+03	3.52E+03	4.46E+03	3.63E+03	3.77E+03
	std	7.42E+00	4.24E+02	1.42E+02	8.88E+02	3.37E+01	1.19E+02	8.43E+01	3.54E+01	1.06E+02	7.03E+01	4.55E+01	1.56E+02	7.30E+01
	median	2.92E+03	4.07E+03	3.48E+03	4.15E+03	3.06E+03	4.00E+03	3.87E+03	3.14E+03	3.19E+03	3.46E+03	4.39E+03	3.47E+03	3.64E+03
	rank	1	11	7	13	2	10	9	3	4	5	12	6	8
C17-F25	mean	2.98E+03	8.66E+03	3.18E+03	1.20E+04	3.07E+03	6.04E+03	4.17E+03	3.06E+03	4.04E+03	4.39E+03	4.29E+03	3.12E+03	4.06E+03
	best	2.98E+03	7.13E+03	3.15E+03	9.65E+03	3.05E+03	4.90E+03	3.75E+03	3.02E+03	3.85E+03	3.90E+03	3.94E+03	3.08E+03	3.95E+03
	worst	2.99E+03	9.63E+03	3.23E+03	1.35E+04	3.09E+03	7.12E+03	4.48E+03	3.08E+03	4.25E+03	4.99E+03	4.96E+03	3.17E+03	4.18E+03
	std	6.30E+00	1.21E+03	3.54E+01	1.96E+03	1.78E+01	1.04E+03	3.36E+02	2.70E+01	2.30E+02	5.99E+02	5.22E+02	5.29E+01	1.05E+02
	median	2.98E+03	8.94E+03	3.17E+03	1.25E+04	3.07E+03	6.07E+03	4.22E+03	3.06E+03	4.04E+03	4.33E+03	4.13E+03	3.12E+03	4.05E+03
	rank	1	12	5	13	3	11	8	2	6	10	9	4	7
C17-F26	mean	3.78E+03	1.42E+04	1.11E+04	1.52E+04	3.32E+03	1.27E+04	1.39E+04	5.82E+03	6.55E+03	9.80E+03	1.16E+04	8.18E+03	9.05E+03
	best	3.75E+03	1.40E+04	1.05E+04	1.46E+04	3.12E+03	1.06E+04	1.30E+04	5.32E+03	6.15E+03	8.98E+03	1.13E+04	7.59E+03	7.16E+03
	worst	3.79E+03	1.44E+04	1.16E+04	1.62E+04	3.62E+03	1.40E+04	1.57E+04	6.09E+03	6.92E+03	1.06E+04	1.21E+04	8.75E+03	1.15E+04
	std	2.12E+01	2.18E+02	4.62E+02	7.52E+02	2.46E+02	1.62E+03	1.30E+03	3.78E+02	4.38E+02	7.23E+02	3.46E+02	5.63E+02	2.25E+03
	median	3.78E+03	1.42E+04	1.11E+04	1.50E+04	3.28E+03	1.32E+04	1.35E+04	5.93E+03	6.56E+03	9.83E+03	1.16E+04	8.19E+03	8.77E+03
	rank	2	12	8	13	1	10	11	3	4	7	9	5	6
C17-F27	mean	3.25E+03	4.82E+03	3.86E+03	5.01E+03	3.39E+03	4.73E+03	4.47E+03	3.36E+03	3.65E+03	3.84E+03	8.15E+03	3.65E+03	4.46E+03
	best	3.23E+03	4.50E+03	3.81E+03	4.63E+03	3.28E+03	4.00E+03	3.89E+03	3.32E+03	3.60E+03	3.64E+03	7.89E+03	3.38E+03	4.35E+03
	worst	3.31E+03	5.04E+03	3.92E+03	5.28E+03	3.49E+03	5.23E+03	5.06E+03	3.44E+03	3.70E+03	4.01E+03	8.51E+03	3.90E+03	4.61E+03
	std	4.54E+01	2.59E+02	5.61E+01	3.39E+02	9.44E+01	5.79E+02	5.98E+02	5.51E+01	5.63E+01	1.77E+02	3.28E+02	2.53E+02	1.20E+02
	median	3.23E+03	4.87E+03	3.85E+03	5.06E+03	3.39E+03	4.84E+03	4.47E+03	3.35E+03	3.64E+03	3.85E+03	8.09E+03	3.66E+03	4.44E+03
	rank	1	11	7	12	3	10	9	2	4	6	13	5	8
C17-F28	mean	3.26E+03	8.79E+03	3.60E+03	1.13E+04	3.35E+03	7.30E+03	4.84E+03	3.29E+03	4.42E+03	5.27E+03	5.08E+03	3.88E+03	5.06E+03
	best	3.26E+03	7.93E+03	3.51E+03	9.97E+03	3.32E+03	5.91E+03	4.22E+03	3.26E+03	4.14E+03	4.64E+03	5.02E+03	3.56E+03	4.81E+03
	worst	3.26E+03	1.10E+04	3.69E+03	1.47E+04	3.40E+03	8.74E+03	5.08E+03	3.31E+03	4.76E+03	5.83E+03	5.20E+03	4.40E+03	5.25E+03
	std	0.00E+00	1.60E+03	9.38E+01	2.50E+03	4.43E+01	1.56E+03	4.49E+02	2.21E+01	3.14E+02	5.29E+02	9.05E+01	3.98E+02	2.36E+02
	median	3.26E+03	8.13E+03	3.59E+03	1.02E+04	3.35E+03	7.28E+03	5.03E+03	3.29E+03	4.38E+03	5.31E+03	5.05E+03	3.78E+03	5.10E+03
	rank	1	12	4	13	3	11	7	2	6	10	9	5	8
C17-F29	mean	3.26E+03	1.38E+04	5.53E+03	1.97E+04	4.11E+03	6.95E+03	9.12E+03	4.86E+03	4.90E+03	6.58E+03	8.24E+03	4.86E+03	6.19E+03
	best	3.25E+03	9.06E+03	5.38E+03	1.04E+04	3.74E+03	6.49E+03	6.13E+03	4.40E+03	4.69E+03	5.66E+03	6.78E+03	4.63E+03	5.87E+03
	worst	3.28E+03	1.89E+04	5.68E+03	3.12E+04	4.35E+03	7.48E+03	1.20E+04	5.47E+03	5.20E+03	7.58E+03	1.08E+04	4.95E+03	6.80E+03
	std	1.90E+01	4.93E+03	1.30E+02	1.01E+04	3.00E+02	4.46E+02	2.61E+03	4.84E+02	2.55E+02	9.90E+02	1.98E+03	1.69E+02	4.74E+02
	median	3.26E+03	1.35E+04	5.53E+03	1.86E+04	4.16E+03	6.91E+03	9.19E+03	4.78E+03	4.85E+03	6.53E+03	7.68E+03	4.93E+03	6.04E+03
	rank	1	12	6	13	2	9	11	3	5	8	10	4	7
C17-F30	mean	6.24E+05	3.28E+09	2.19E+07	5.50E+09	1.66E+06	1.66E+09	1.59E+08	7.05E+07	1.40E+08	3.01E+08	1.85E+08	4.82E+06	5.85E+07
	best	5.82E+05	2.53E+09	1.34E+07	3.37E+09	1.25E+06	2.03E+08	1.07E+08	6.37E+07	6.75E+07	2.09E+08	1.41E+08	3.36E+06	4.72E+07
	worst	6.56E+05	4.45E+09	3.00E+07	8.62E+09	2.71E+06	3.37E+09	2.19E+08	8.11E+07	2.07E+08	3.81E+08	2.42E+08	6.70E+06	8.21E+07
	std	3.55E+04	9.10E+08	8.88E+06	2.46E+09	7.62E+05	1.77E+09	6.10E+07	8.21E+06	7.67E+07	7.80E+07	4.59E+07	1.79E+06	1.76E+07
	median	6.28E+05	3.06E+09	2.21E+07	4.99E+09	1.34E+06	1.53E+09	1.54E+08	6.86E+07	1.42E+08	3.06E+08	1.78E+08	4.60E+06	5.23E+07
	rank	1	12	4	13	2	11	8	6	7	10	9	3	5
Sum rank		30	335	166	367	63	294	269	112	144	248	254	150	207
Mean rank		1.03E+00	1.16E+01	5.72E+00	1.27E+01	2.17E+00	1.01E+01	9.28E+00	3.86E+00	4.97E+00	8.55E+00	8.76E+00	5.17E+00	7.14E+00
Total rank		1	12	6	13	2	11	10	3	4	8	9	5	7

**Table 5:** Optimization results of the CEC 2017 test suite (dimensions = 100).

		TVETBO	WSO	AVOA	RSA	MPA	TSA	WOA	MVO	GWO	TLBO	GSA	PSO	GA
C17-F1	mean	1.00E+02	1.67E+11	3.82E+09	2.33E+11	5.19E+08	1.26E+11	6.27E+10	6.58E+07	5.71E+10	9.11E+10	1.36E+11	2.00E+10	5.61E+10
	best	1.00E+02	1.63E+11	1.86E+09	2.29E+11	3.93E+08	1.11E+11	5.92E+10	5.48E+07	4.95E+10	8.67E+10	1.25E+11	1.35E+10	5.31E+10
	worst	1.00E+02	1.71E+11	5.50E+09	2.35E+11	6.56E+08	1.41E+11	7.02E+10	7.70E+07	6.46E+10	1.00E+11	1.46E+11	2.72E+10	6.34E+10
	std	1.26E-14	3.71E+09	1.63E+09	2.92E+09	1.38E+08	1.35E+10	5.49E+09	1.18E+07	7.81E+09	6.87E+09	9.44E+09	8.22E+09	5.33E+09
	median	1.00E+02	1.67E+11	3.97E+09	2.34E+11	5.14E+08	1.26E+11	6.07E+10	6.56E+07	5.71E+10	8.87E+10	1.37E+11	1.97E+10	5.39E+10
	rank	1	12	4	13	3	10	8	2	7	9	11	5	6
C17-F3	mean	3.00E+02	4.28E+05	3.26E+05	3.22E+05	1.58E+05	3.63E+05	7.88E+05	4.66E+05	3.68E+05	2.96E+05	3.43E+05	5.40E+05	5.77E+05
	best	3.00E+02	3.90E+05	3.18E+05	3.11E+05	1.21E+05	2.91E+05	6.90E+05	3.87E+05	3.37E+05	2.77E+05	3.17E+05	4.09E+05	5.53E+05
	worst	3.00E+02	4.47E+05	3.33E+05	3.29E+05	1.91E+05	4.15E+05	9.13E+05	5.57E+05	4.03E+05	3.13E+05	3.75E+05	7.58E+05	5.95E+05
	std	0.00E+00	2.93E+04	6.86E+03	9.22E+03	3.31E+04	5.70E+04	1.05E+05	9.45E+04	3.85E+04	1.58E+04	2.62E+04	1.76E+05	2.04E+04
	median	3.00E+02	4.37E+05	3.26E+05	3.25E+05	1.60E+05	3.73E+05	7.75E+05	4.59E+05	3.66E+05	2.96E+05	3.40E+05	4.97E+05	5.79E+05
	rank	1	9	5	4	2	7	13	10	8	3	6	11	12
C17-F4	mean	6.02E+02	4.45E+04	1.55E+03	7.50E+04	1.02E+03	1.60E+04	1.09E+04	7.60E+02	4.46E+03	1.07E+04	3.41E+04	2.47E+03	9.19E+03
	best	5.92E+02	4.09E+04	1.30E+03	6.80E+04	9.06E+02	1.05E+04	9.31E+03	7.05E+02	3.43E+03	1.02E+04	2.71E+04	1.49E+03	8.69E+03
	worst	6.12E+02	4.88E+04	1.71E+03	8.36E+04	1.13E+03	2.13E+04	1.20E+04	8.21E+02	6.70E+03	1.16E+04	3.85E+04	3.12E+03	9.77E+03
	std	1.27E+01	3.66E+03	2.01E+02	7.03E+03	1.20E+02	4.85E+03	1.24E+03	5.24E+01	1.64E+03	7.13E+02	6.02E+03	7.61E+02	5.46E+02
	median	6.02E+02	4.41E+04	1.60E+03	7.42E+04	1.02E+03	1.61E+04	1.12E+04	7.57E+02	3.86E+03	1.05E+04	3.53E+04	2.63E+03	9.16E+03
	rank	1	12	4	13	3	10	9	2	6	8	11	5	7
C17-F5	mean	5.13E+02	1.95E+03	1.29E+03	1.92E+03	1.20E+03	2.10E+03	1.80E+03	1.21E+03	1.16E+03	1.84E+03	1.31E+03	1.38E+03	1.55E+03
	best	5.11E+02	1.93E+03	1.27E+03	1.89E+03	1.07E+03	2.08E+03	1.71E+03	1.10E+03	1.10E+03	1.81E+03	1.27E+03	1.29E+03	1.41E+03
	worst	5.15E+02	1.96E+03	1.30E+03	1.96E+03	1.28E+03	2.13E+03	1.95E+03	1.28E+03	1.21E+03	1.86E+03	1.34E+03	1.55E+03	1.64E+03
	std	1.98E+00	1.43E+01	9.55E+00	3.80E+01	1.10E+02	2.69E+01	1.16E+02	8.64E+01	4.93E+01	2.48E+01	3.65E+01	1.37E+02	1.13E+02
	median	5.13E+02	1.96E+03	1.29E+03	1.92E+03	1.22E+03	2.10E+03	1.77E+03	1.23E+03	1.16E+03	1.83E+03	1.31E+03	1.35E+03	1.58E+03
	rank	1	12	5	11	3	13	9	4	2	10	6	7	8
C17-F6	mean	6.00E+02	7.03E+02	6.60E+02	7.02E+02	6.36E+02	7.08E+02	7.01E+02	6.72E+02	6.39E+02	6.79E+02	6.62E+02	6.60E+02	6.61E+02
	best	6.00E+02	7.01E+02	6.56E+02	6.97E+02	6.33E+02	6.96E+02	6.91E+02	6.66E+02	6.34E+02	6.70E+02	6.60E+02	6.53E+02	6.54E+02
	worst	6.00E+02	7.06E+02	6.64E+02	7.05E+02	6.43E+02	7.16E+02	7.18E+02	6.79E+02	6.45E+02	6.84E+02	6.66E+02	6.65E+02	6.67E+02
	std	0.00E+00	2.51E+00	3.64E+00	3.68E+00	5.18E+00	1.07E+01	1.29E+01	5.94E+00	5.21E+00	7.16E+00	3.19E+00	6.60E+00	6.90E+00
	median	6.00E+02	7.03E+02	6.60E+02	7.03E+02	6.35E+02	7.10E+02	6.97E+02	6.73E+02	6.38E+02	6.81E+02	6.61E+02	6.60E+02	6.62E+02
	rank	1	12	5	11	2	13	10	8	3	9	7	4	6
C17-F7	mean	8.11E+02	3.53E+03	3.01E+03	3.64E+03	1.81E+03	3.36E+03	3.50E+03	1.97E+03	1.98E+03	3.03E+03	3.05E+03	2.42E+03	2.52E+03
	best	8.10E+02	3.44E+03	2.86E+03	3.55E+03	1.75E+03	3.18E+03	3.38E+03	1.81E+03	1.80E+03	2.89E+03	2.93E+03	2.16E+03	2.42E+03
	worst	8.13E+02	3.63E+03	3.14E+03	3.72E+03	1.89E+03	3.52E+03	3.67E+03	2.09E+03	2.12E+03	3.15E+03	3.27E+03	2.54E+03	2.74E+03
	std	1.59E+00	8.29E+01	1.56E+02	7.71E+01	6.43E+01	1.67E+02	1.45E+02	1.26E+02	1.47E+02	1.16E+02	1.62E+02	1.95E+02	1.58E+02
	median	8.11E+02	3.52E+03	3.03E+03	3.65E+03	1.80E+03	3.36E+03	3.47E+03	1.99E+03	2.01E+03	3.04E+03	3.01E+03	2.50E+03	2.46E+03
	rank	1	12	7	13	2	10	11	3	4	8	9	5	6
C17-F8	mean	8.12E+02	2.37E+03	1.71E+03	2.42E+03	1.41E+03	2.35E+03	2.27E+03	1.43E+03	1.49E+03	2.21E+03	1.79E+03	1.68E+03	1.99E+03
	best	8.09E+02	2.32E+03	1.65E+03	2.40E+03	1.24E+03	2.28E+03	2.07E+03	1.28E+03	1.39E+03	2.14E+03	1.71E+03	1.64E+03	1.94E+03
	worst	8.17E+02	2.43E+03	1.73E+03	2.44E+03	1.51E+03	2.43E+03	2.42E+03	1.61E+03	1.63E+03	2.26E+03	1.92E+03	1.77E+03	2.04E+03
	std	3.70E+00	5.07E+01	4.11E+01	1.86E+01	1.29E+02	7.97E+01	1.93E+02	1.47E+02	1.18E+02	5.39E+01	1.01E+02	6.79E+01	4.68E+01
	median	8.12E+02	2.37E+03	1.72E+03	2.43E+03	1.44E+03	2.34E+03	2.29E+03	1.42E+03	1.48E+03	2.21E+03	1.77E+03	1.65E+03	1.99E+03
	rank	1	12	6	13	2	11	10	3	4	9	7	5	8
C17-F9	mean	9.00E+02	8.69E+04	2.56E+04	7.45E+04	2.16E+04	1.16E+05	7.41E+04	5.71E+04	3.47E+04	7.18E+04	2.27E+04	3.17E+04	4.44E+04
	best	9.00E+02	7.76E+04	2.13E+04	7.20E+04	2.01E+04	9.53E+04	5.76E+04	4.82E+04	2.15E+04	6.88E+04	2.12E+04	2.68E+04	4.02E+04
	worst	9.00E+02	1.00E+05	2.88E+04	7.66E+04	2.23E+04	1.45E+05	9.33E+04	6.50E+04	4.72E+04	7.34E+04	2.40E+04	3.53E+04	5.00E+04
	std	1.01E-13	1.07E+04	3.40E+03	2.14E+03	1.10E+03	2.27E+04	1.94E+04	7.53E+03	1.37E+04	2.31E+03	1.27E+03	4.12E+03	4.48E+03
	median	9.00E+02	8.49E+04	2.61E+04	7.48E+04	2.21E+04	1.12E+05	7.27E+04	5.77E+04	3.51E+04	7.25E+04	2.29E+04	3.23E+04	4.36E+04
	rank	1	12	4	11	2	13	10	8	6	9	3	5	7
C17-F10	mean	1.10E+04	2.98E+04	1.58E+04	3.11E+04	1.38E+04	2.89E+04	2.79E+04	1.68E+04	1.51E+04	3.11E+04	1.70E+04	1.69E+04	2.57E+04
	best	9.63E+03	2.95E+04	1.32E+04	3.02E+04	1.31E+04	2.82E+04	2.70E+04	1.62E+04	1.40E+04	2.99E+04	1.53E+04	1.51E+04	2.52E+04
	worst	1.19E+04	3.00E+04	1.80E+04	3.15E+04	1.46E+04	2.98E+04	2.92E+04	1.74E+04	1.56E+04	3.21E+04	1.80E+04	1.82E+04	2.62E+04
	std	1.05E+03	2.66E+02	2.29E+03	6.69E+02	6.87E+02	7.83E+02	1.09E+03	5.50E+02	8.13E+02	1.02E+03	1.38E+03	1.40E+03	4.51E+02
	median	1.13E+04	2.98E+04	1.60E+04	3.13E+04	1.37E+04	2.88E+04	2.76E+04	1.68E+04	1.53E+04	3.11E+04	1.74E+04	1.71E+04	2.57E+04
	rank	1	11	4	12	2	10	9	5	3	13	7	6	8
C17-F11	mean	1.16E+03	1.61E+05	6.28E+04	2.02E+05	4.72E+03	6.40E+04	2.04E+05	4.52E+03	8.54E+04	7.03E+04	1.69E+05	5.10E+04	1.36E+05
	best	1.14E+03	1.25E+05	5.64E+04	1.55E+05	3.72E+03	2.92E+04	1.19E+05	3.93E+03	7.09E+04	5.93E+04	1.41E+05	2.32E+04	1.04E+05
	worst	1.22E+03	1.88E+05	7.51E+04	2.88E+05	5.63E+03	9.16E+04	3.29E+05	4.79E+03	9.62E+04	8.96E+04	1.97E+05	1.04E+05	1.88E+05
	std	4.25E+01	2.92E+04	9.37E+03	6.54E+04	8.97E+02	2.81E+04	1.06E+05	4.28E+02	1.18E+04	1.45E+04	2.53E+04	3.94E+04	4.00E+04
	median	1.14E+03	1.66E+05	5.99E+04	1.83E+05	4.76E+03	6.77E+04	1.84E+05	4.68E+03	8.72E+04	6.61E+04	1.69E+05	3.83E+04	1.27E+05
	rank	1	10	5	12	3	6	13	2	8	7	11	4	9
C17-F12	mean	5.97E+03	1.03E+11	6.45E+08	1.68E+11	2.55E+08	5.57E+10	1.29E+10	3.26E+08	1.12E+10	2.15E+10	6.55E+10	9.89E+09	1.21E+10
	best	5.38E+03	7.34E+10	3.42E+08	1.26E+11	1.42E+08	2.86E+10	1.05E+10	2.07E+08	7.76E+09	1.69E+10	5.68E+10	1.28E+09	1.10E+10
	worst	6.57E+03	1.15E+11	1.03E+09	1.95E+11	3.06E+08	9.24E+10	1.48E+10	5.12E+08	1.33E+10	2.96E+10	7.70E+10	1.88E+10	1.43E+10
	std	5.38E+02	2.18E+10	3.23E+08	3.47E+10	8.25E+07	2.90E+10	1.96E+09	1.46E+08	2.62E+09	6.33E+09	9.16E+09	8.68E+09	1.62E+09
	median	5.97E+03	1.12E+11	6.04E+08	1.76E+11	2.86E+08	5.09E+10	1.32E+10	2.92E+08	1.19E+10	1.97E+10	6.40E+10	9.74E+09	1.15E+10
	rank	1	12	4	13	2	10	8	3	6	9	11	5	7
C17-F13	mean	1.41E+03	2.73E+10	9.63E+04	4.18E+10	9.50E+04	2.09E+10	5.12E+08	3.47E+05	9.28E+08	2.76E+09	8.56E+09	1.73E+09	1.71E+08
	best	1.37E+03	2.38E+10	6.80E+04	3.24E+10	4.07E+04	1.49E+10	3.65E+08	3.06E+05	8.01E+07	1.91E+09	5.26E+09	1.91E+08	1.34E+08
	worst	1.44E+03	3.03E+10	1.31E+05	4.74E+10	2.36E+05	2.51E+10	6.93E+08	4.05E+05	2.45E+09	3.34E+09	1.10E+10	3.13E+09	2.06E+08
	std	3.78E+01	3.69E+09	2.92E+04	7.57E+09	1.03E+05	4.70E+09	1.84E+08	4.71E+04	1.19E+09	7.10E+08	2.61E+09	1.57E+09	4.05E+07
	median	1.41E+03	2.76E+10	9.30E+04	4.38E+10	5.17E+04	2.19E+10	4.96E+08	3.39E+05	5.90E+08	2.90E+09	8.99E+09	1.80E+09	1.72E+08
	rank	1	12	3	13	2	11	6	4	7	9	10	8	5
C17-F14	mean	1.47E+03	4.46E+07	6.55E+06	7.82E+07	8.92E+04	8.74E+06	1.43E+07	2.98E+06	9.44E+06	1.37E+07	1.13E+07	8.00E+05	1.03E+07
	best	1.46E+03	3.85E+07	3.97E+06	7.13E+07	2.55E+04	3.97E+06	8.22E+06	8.99E+05	5.97E+06	1.02E+07	8.70E+06	3.80E+05	5.77E+06
	worst	1.47E+03	5.09E+07	1.09E+07	8.56E+07	1.90E+05	1.70E+07	1.95E+07	4.10E+06	1.42E+07	1.75E+07	1.69E+07	1.66E+06	1.52E+07
	std	6.58E+00	5.94E+06	3.31E+06	7.47E+06	8.00E+04	6.27E+06	5.06E+06	1.55E+06	3.90E+06	4.14E+06	4.14E+06	6.35E+05	4.27E+06
	median	1.47E+03	4.45E+07	5.68E+06	7.80E+07	7.09E+04	6.96E+06	1.47E+07	3.46E+06	8.82E+06	1.35E+07	9.76E+06	5.80E+05	1.01E+07
	rank	1	12	5	13	2	6	11	4	7	10	9	3	8
C17-F15	mean	1.61E+03	1.51E+10	8.28E+04	2.31E+10	5.50E+04	1.18E+10	6.88E+07	1.24E+05	4.92E+08	1.17E+09	1.22E+09	3.28E+08	1.25E+07
	best	1.55E+03	1.40E+10	6.77E+04	1.65E+10	1.59E+04	2.46E+08	3.83E+07	8.50E+04	3.23E+07	3.91E+08	4.88E+08	6.04E+04	8.03E+06
	worst	1.65E+03	1.70E+10	1.04E+05	2.88E+10	8.35E+04	2.22E+10	1.32E+08	1.83E+05	1.48E+09	2.50E+09	1.56E+09	1.29E+09	2.12E+07
	std	4.80E+01	1.43E+09	1.89E+04	6.64E+09	3.11E+04	1.04E+10	4.67E+07	4.69E+04	7.26E+08	1.01E+09	5.39E+08	7.00E+08	6.52E+06
	median	1.62E+03	1.48E+10	7.98E+04	2.36E+10	6.03E+04	1.25E+10	5.24E+07	1.15E+05	2.31E+08	8.95E+08	1.41E+09	8.48E+06	1.03E+07
	rank	1	12	3	13	2	11	6	4	8	9	10	7	5
C17-F16	mean	2.71E+03	1.87E+04	7.06E+03	2.23E+04	5.48E+03	1.44E+04	1.60E+04	6.53E+03	6.05E+03	1.13E+04	1.09E+04	6.42E+03	1.04E+04
	best	2.17E+03	1.73E+04	5.87E+03	1.75E+04	5.38E+03	1.19E+04	1.30E+04	5.79E+03	5.46E+03	1.08E+04	9.46E+03	6.12E+03	9.36E+03
	worst	3.40E+03	1.93E+04	7.79E+03	2.50E+04	5.62E+03	1.72E+04	1.77E+04	7.03E+03	6.74E+03	1.24E+04	1.26E+04	6.66E+03	1.12E+04
	std	5.55E+02	9.64E+02	9.12E+02	3.68E+03	1.11E+02	2.39E+03	2.28E+03	5.92E+02	7.09E+02	8.27E+02	1.55E+03	2.43E+02	9.17E+02
	median	2.64E+03	1.90E+04	7.29E+03	2.34E+04	5.46E+03	1.42E+04	1.66E+04	6.66E+03	5.99E+03	1.11E+04	1.08E+04	6.45E+03	1.05E+04
	rank	1	12	6	13	2	10	11	5	3	9	8	4	7
C17-F17	mean	2.72E+03	4.15E+06	5.79E+03	8.16E+06	4.61E+03	2.15E+05	1.68E+04	4.93E+03	5.45E+03	8.64E+03	4.57E+04	6.04E+03	7.08E+03
	best	2.28E+03	1.22E+06	5.59E+03	2.21E+06	4.40E+03	1.01E+04	1.03E+04	4.49E+03	4.39E+03	8.52E+03	2.99E+04	5.77E+03	6.87E+03
	worst	3.43E+03	9.44E+06	6.25E+03	1.88E+07	4.84E+03	5.71E+05	2.84E+04	5.26E+03	7.05E+03	8.78E+03	7.42E+04	6.30E+03	7.26E+03
	std	5.60E+02	4.22E+06	3.40E+02	8.48E+06	2.31E+02	2.67E+05	8.84E+03	4.09E+02	1.27E+03	1.51E+02	2.13E+04	2.48E+02	1.80E+02
	median	2.58E+03	2.97E+06	5.66E+03	5.83E+06	4.60E+03	1.39E+05	1.42E+04	4.98E+03	5.19E+03	8.63E+03	3.93E+04	6.04E+03	7.09E+03
	rank	1	12	5	13	2	11	9	3	4	8	10	6	7
C17-F18	mean	1.90E+03	5.74E+07	2.77E+06	1.01E+08	2.28E+05	1.47E+07	1.18E+07	4.83E+06	1.08E+07	1.59E+07	1.16E+07	6.33E+06	5.94E+06
	best	1.88E+03	2.60E+07	1.38E+06	3.93E+07	1.59E+05	5.49E+06	8.78E+06	3.57E+06	3.39E+06	1.17E+07	5.33E+06	3.91E+06	4.76E+06
	worst	1.92E+03	1.04E+08	4.38E+06	1.85E+08	4.11E+05	2.99E+07	1.40E+07	8.11E+06	1.74E+07	2.25E+07	2.57E+07	9.12E+06	8.59E+06
	std	2.11E+01	3.62E+07	1.48E+06	6.69E+07	1.33E+05	1.20E+07	2.58E+06	2.39E+06	6.28E+06	5.04E+06	1.04E+07	2.63E+06	1.96E+06
	median	1.91E+03	4.99E+07	2.66E+06	9.03E+07	1.71E+05	1.16E+07	1.22E+07	3.81E+06	1.11E+07	1.47E+07	7.60E+06	6.14E+06	5.20E+06
	rank	1	12	3	13	2	10	9	4	7	11	8	6	5
C17-F19	mean	1.97E+03	1.25E+10	2.83E+06	2.20E+10	2.75E+05	4.97E+09	1.32E+08	1.64E+07	3.55E+08	6.58E+08	1.55E+09	2.65E+08	1.26E+07
	best	1.97E+03	1.10E+10	1.08E+06	1.61E+10	5.80E+04	2.20E+09	5.23E+07	9.55E+06	2.82E+06	2.86E+08	2.80E+08	4.41E+07	6.43E+06
	worst	1.98E+03	1.47E+10	5.21E+06	2.74E+10	4.67E+05	9.87E+09	2.22E+08	2.60E+07	1.07E+09	1.51E+09	2.93E+09	5.74E+08	2.28E+07
	std	4.94E+00	1.81E+09	1.90E+06	5.08E+09	1.85E+05	3.69E+09	8.56E+07	8.85E+06	5.41E+08	6.28E+08	1.44E+09	2.79E+08	7.89E+06
	median	1.97E+03	1.22E+10	2.52E+06	2.23E+10	2.89E+05	3.90E+09	1.26E+08	1.50E+07	1.75E+08	4.17E+08	1.50E+09	2.21E+08	1.06E+07
	rank	1	12	3	13	2	11	6	5	8	9	10	7	4
C17-F20	mean	3.19E+03	7.24E+03	6.14E+03	7.49E+03	4.48E+03	6.98E+03	7.00E+03	5.78E+03	6.04E+03	7.20E+03	6.29E+03	5.35E+03	6.24E+03
	best	2.81E+03	7.05E+03	5.77E+03	7.36E+03	4.43E+03	6.37E+03	6.54E+03	5.46E+03	4.80E+03	6.40E+03	5.82E+03	4.63E+03	5.64E+03
	worst	3.66E+03	7.41E+03	6.44E+03	7.56E+03	4.56E+03	7.74E+03	7.37E+03	6.33E+03	6.93E+03	7.56E+03	6.56E+03	6.20E+03	6.73E+03
	std	4.78E+02	1.61E+02	3.37E+02	9.79E+01	5.86E+01	6.40E+02	3.94E+02	4.12E+02	1.16E+03	5.82E+02	3.59E+02	7.28E+02	5.65E+02
	median	3.15E+03	7.25E+03	6.18E+03	7.52E+03	4.47E+03	6.91E+03	7.04E+03	5.67E+03	6.22E+03	7.42E+03	6.38E+03	5.28E+03	6.29E+03
	rank	1	12	6	13	2	9	10	4	5	11	8	3	7
C17-F21	mean	2.34E+03	4.25E+03	3.64E+03	4.37E+03	2.82E+03	4.09E+03	4.19E+03	3.22E+03	2.97E+03	3.68E+03	4.67E+03	3.56E+03	3.40E+03
	best	2.34E+03	4.21E+03	3.43E+03	4.30E+03	2.78E+03	3.95E+03	3.89E+03	3.15E+03	2.88E+03	3.52E+03	4.12E+03	3.37E+03	3.36E+03
	worst	2.35E+03	4.32E+03	3.78E+03	4.43E+03	2.86E+03	4.19E+03	4.42E+03	3.35E+03	3.02E+03	3.87E+03	5.12E+03	3.91E+03	3.45E+03
	std	3.66E+00	6.05E+01	1.62E+02	6.28E+01	3.65E+01	1.28E+02	2.57E+02	9.68E+01	6.24E+01	1.58E+02	4.50E+02	2.65E+02	3.98E+01
	median	2.34E+03	4.24E+03	3.68E+03	4.38E+03	2.83E+03	4.11E+03	4.23E+03	3.19E+03	2.98E+03	3.67E+03	4.73E+03	3.48E+03	3.39E+03
	rank	1	11	7	12	2	9	10	4	3	8	13	6	5
C17-F22	mean	1.17E+04	3.14E+04	2.01E+04	3.31E+04	1.86E+04	3.04E+04	2.88E+04	1.72E+04	2.32E+04	3.30E+04	2.10E+04	2.18E+04	2.85E+04
	best	1.11E+04	3.06E+04	1.87E+04	3.27E+04	1.72E+04	2.93E+04	2.73E+04	1.62E+04	1.84E+04	3.20E+04	2.03E+04	2.03E+04	2.75E+04
	worst	1.26E+04	3.19E+04	2.20E+04	3.37E+04	2.03E+04	3.15E+04	3.00E+04	1.79E+04	3.42E+04	3.34E+04	2.13E+04	2.34E+04	2.93E+04
	std	7.10E+02	6.32E+02	1.58E+03	4.97E+02	1.39E+03	9.96E+02	1.29E+03	9.31E+02	8.16E+03	7.19E+02	5.41E+02	1.38E+03	9.37E+02
	median	1.16E+04	3.16E+04	1.99E+04	3.30E+04	1.84E+04	3.05E+04	2.90E+04	1.74E+04	2.00E+04	3.32E+04	2.12E+04	2.17E+04	2.86E+04
	rank	1	11	4	13	3	10	9	2	7	12	5	6	8
C17-F23	mean	2.88E+03	5.31E+03	4.10E+03	5.32E+03	3.29E+03	5.43E+03	5.13E+03	3.48E+03	3.61E+03	4.20E+03	7.85E+03	4.85E+03	4.25E+03
	best	2.87E+03	5.06E+03	4.02E+03	5.05E+03	3.28E+03	4.67E+03	4.98E+03	3.39E+03	3.58E+03	4.15E+03	7.25E+03	4.33E+03	4.19E+03
	worst	2.88E+03	5.61E+03	4.19E+03	5.53E+03	3.32E+03	6.46E+03	5.27E+03	3.60E+03	3.66E+03	4.28E+03	8.27E+03	5.13E+03	4.32E+03
	std	5.67E+00	2.66E+02	8.52E+01	2.15E+02	2.30E+01	8.71E+02	1.50E+02	9.69E+01	3.90E+01	6.10E+01	5.01E+02	3.91E+02	7.79E+01
	median	2.88E+03	5.29E+03	4.10E+03	5.34E+03	3.29E+03	5.30E+03	5.13E+03	3.47E+03	3.61E+03	4.19E+03	7.94E+03	4.97E+03	4.25E+03
	rank	1	10	5	11	2	12	9	3	4	6	13	8	7
C17-F24	mean	3.33E+03	8.51E+03	5.37E+03	1.05E+04	3.71E+03	6.66E+03	6.37E+03	3.97E+03	4.29E+03	4.75E+03	1.08E+04	5.95E+03	5.37E+03
	best	3.30E+03	6.63E+03	5.15E+03	7.01E+03	3.67E+03	6.17E+03	5.95E+03	3.90E+03	4.05E+03	4.51E+03	1.01E+04	5.58E+03	5.28E+03
	worst	3.36E+03	9.79E+03	5.55E+03	1.28E+04	3.78E+03	6.99E+03	7.01E+03	4.08E+03	4.51E+03	4.98E+03	1.25E+04	6.43E+03	5.54E+03
	std	3.22E+01	1.64E+03	1.95E+02	3.04E+03	5.92E+01	3.79E+02	5.05E+02	9.28E+01	2.56E+02	2.09E+02	1.25E+03	4.16E+02	1.30E+02
	median	3.33E+03	8.82E+03	5.39E+03	1.11E+04	3.71E+03	6.74E+03	6.26E+03	3.95E+03	4.30E+03	4.75E+03	1.03E+04	5.90E+03	5.33E+03
	rank	1	11	6	12	2	10	9	3	4	5	13	8	7
C17-F25	mean	3.19E+03	1.52E+04	4.14E+03	2.13E+04	3.68E+03	1.05E+04	7.29E+03	3.41E+03	6.42E+03	8.89E+03	1.10E+04	4.14E+03	7.88E+03
	best	3.14E+03	1.45E+04	3.76E+03	1.97E+04	3.51E+03	9.80E+03	6.67E+03	3.34E+03	6.26E+03	7.66E+03	1.02E+04	3.87E+03	7.16E+03
	worst	3.26E+03	1.70E+04	4.50E+03	2.47E+04	3.81E+03	1.09E+04	7.68E+03	3.48E+03	6.83E+03	1.05E+04	1.25E+04	4.57E+03	8.61E+03
	std	6.52E+01	1.29E+03	3.32E+02	2.57E+03	1.37E+02	5.32E+02	4.95E+02	6.15E+01	2.96E+02	1.43E+03	1.15E+03	3.64E+02	8.17E+02
	median	3.17E+03	1.47E+04	4.15E+03	2.03E+04	3.71E+03	1.06E+04	7.41E+03	3.41E+03	6.30E+03	8.69E+03	1.07E+04	4.06E+03	7.87E+03
	rank	1	12	4	13	3	10	7	2	6	9	11	5	8
C17-F26	mean	5.76E+03	3.94E+04	2.46E+04	4.53E+04	1.16E+04	3.32E+04	3.38E+04	1.18E+04	1.68E+04	2.38E+04	3.37E+04	2.07E+04	2.29E+04
	best	5.65E+03	3.88E+04	2.17E+04	4.28E+04	1.09E+04	3.20E+04	3.03E+04	1.05E+04	1.49E+04	1.95E+04	3.24E+04	1.85E+04	2.13E+04
	worst	5.84E+03	3.99E+04	2.75E+04	4.69E+04	1.24E+04	3.40E+04	3.68E+04	1.42E+04	1.84E+04	2.93E+04	3.56E+04	2.27E+04	2.40E+04
	std	9.13E+01	4.77E+02	2.69E+03	2.16E+03	7.94E+02	9.62E+02	3.48E+03	1.79E+03	1.60E+03	4.45E+03	1.47E+03	1.90E+03	1.28E+03
	median	5.77E+03	3.94E+04	2.45E+04	4.58E+04	1.16E+04	3.35E+04	3.41E+04	1.13E+04	1.70E+04	2.32E+04	3.35E+04	2.07E+04	2.32E+04
	rank	1	12	8	13	2	9	11	3	4	7	10	5	6
C17-F27	mean	3.31E+03	9.33E+03	4.16E+03	1.23E+04	3.53E+03	6.61E+03	6.01E+03	3.62E+03	4.08E+03	4.33E+03	1.41E+04	4.08E+03	5.49E+03
	best	3.28E+03	7.84E+03	3.99E+03	9.19E+03	3.49E+03	6.30E+03	5.30E+03	3.58E+03	3.91E+03	4.05E+03	1.37E+04	3.87E+03	5.22E+03
	worst	3.34E+03	1.08E+04	4.45E+03	1.55E+04	3.57E+03	6.98E+03	6.79E+03	3.72E+03	4.22E+03	4.79E+03	1.43E+04	4.28E+03	5.89E+03
	std	3.09E+01	1.76E+03	2.17E+02	3.70E+03	3.36E+01	3.21E+02	8.74E+02	7.22E+01	1.63E+02	3.59E+02	3.04E+02	2.46E+02	3.08E+02
	median	3.31E+03	9.32E+03	4.11E+03	1.22E+04	3.54E+03	6.57E+03	5.97E+03	3.60E+03	4.10E+03	4.24E+03	1.41E+04	4.07E+03	5.42E+03
	rank	1	11	6	12	2	10	9	3	5	7	13	4	8
C17-F28	mean	3.32E+03	2.09E+04	4.71E+03	2.82E+04	3.77E+03	1.57E+04	1.04E+04	3.46E+03	9.27E+03	1.12E+04	1.88E+04	7.65E+03	1.15E+04
	best	3.32E+03	1.95E+04	4.40E+03	2.53E+04	3.65E+03	1.23E+04	8.86E+03	3.37E+03	7.85E+03	8.72E+03	1.62E+04	5.18E+03	1.05E+04
	worst	3.33E+03	2.36E+04	4.93E+03	3.19E+04	3.86E+03	1.83E+04	1.14E+04	3.54E+03	1.13E+04	1.33E+04	2.08E+04	1.18E+04	1.26E+04
	std	4.77E+00	2.04E+03	2.43E+02	3.03E+03	9.77E+01	3.11E+03	1.16E+03	7.50E+01	1.59E+03	2.34E+03	2.06E+03	3.30E+03	1.27E+03
	median	3.32E+03	2.03E+04	4.75E+03	2.79E+04	3.79E+03	1.61E+04	1.06E+04	3.46E+03	8.97E+03	1.13E+04	1.91E+04	6.79E+03	1.14E+04
	rank	1	12	4	13	3	10	7	2	6	8	11	5	9
C17-F29	mean	4.45E+03	1.83E+05	9.61E+03	3.49E+05	6.87E+03	1.84E+04	1.65E+04	8.67E+03	8.29E+03	1.24E+04	2.49E+04	8.63E+03	1.18E+04
	best	4.17E+03	1.04E+05	8.33E+03	1.87E+05	6.06E+03	1.41E+04	1.38E+04	7.72E+03	8.12E+03	1.16E+04	2.05E+04	7.97E+03	1.16E+04
	worst	4.83E+03	2.50E+05	1.04E+04	4.84E+05	7.65E+03	2.33E+04	1.89E+04	9.32E+03	8.61E+03	1.30E+04	3.27E+04	9.54E+03	1.23E+04
	std	3.07E+02	6.75E+04	9.67E+02	1.38E+05	7.09E+02	4.19E+03	2.76E+03	7.56E+02	2.41E+02	6.77E+02	6.15E+03	8.10E+02	3.44E+02
	median	4.40E+03	1.89E+05	9.86E+03	3.62E+05	6.89E+03	1.81E+04	1.67E+04	8.82E+03	8.22E+03	1.25E+04	2.32E+04	8.51E+03	1.17E+04
	rank	1	12	6	13	2	10	9	5	3	8	11	4	7
C17-F30	mean	5.41E+03	2.31E+10	2.76E+07	3.76E+10	4.68E+06	1.33E+10	1.49E+09	1.03E+08	1.83E+09	3.77E+09	7.32E+09	6.02E+08	6.63E+08
	best	5.34E+03	2.02E+10	1.57E+07	3.51E+10	2.08E+06	8.12E+09	1.23E+09	6.31E+07	7.51E+08	1.42E+09	5.22E+09	1.47E+08	5.53E+08
	worst	5.56E+03	2.51E+10	4.86E+07	4.06E+10	7.64E+06	1.65E+10	2.02E+09	1.26E+08	2.39E+09	6.99E+09	8.86E+09	1.87E+09	7.11E+08
	std	1.10E+02	2.22E+09	1.60E+07	2.59E+09	2.79E+06	4.00E+09	3.92E+08	3.05E+07	8.03E+08	3.04E+09	1.67E+09	9.18E+08	8.03E+07
	median	5.37E+03	2.35E+10	2.31E+07	3.73E+10	4.49E+06	1.44E+10	1.36E+09	1.11E+08	2.09E+09	3.34E+09	7.60E+09	1.98E+08	6.94E+08
	rank	1	12	3	13	2	11	7	4	8	9	10	5	6
Sum rank		29	336	140	355	65	293	265	114	156	249	272	162	203
Mean rank		1.00E+00	1.16E+01	4.83E+00	1.22E+01	2.24E+00	1.01E+01	9.14E+00	3.93E+00	5.38E+00	8.59E+00	9.38E+00	5.59E+00	7.00E+00
Total rank		1	12	4	13	2	11	9	3	5	8	10	6	7

**Table 6:** Wilcoxon rank sum test results.

Compared Algorithm	Objective Function Type			
	CEC 2017			
	D = 10	D = 30	D = 50	D = 100
TVETBO vs. WSO	2.02E-21	1.97E-21	1.97E-21	1.97E-21
TVETBO vs. AVOA	3.77E-19	3.02E-21	1.97E-21	1.97E-21
TVETBO vs. RSA	1.97E-21	1.97E-21	1.97E-21	1.97E-21
TVETBO vs. MPA	2E-18	1.56E-16	6.62E-18	1.97E-21
TVETBO vs. TSA	9.5E-21	1.97E-21	1.97E-21	1.97E-21
TVETBO vs. WOA	9.5E-21	1.97E-21	1.97E-21	1.97E-21
TVETBO vs. MVO	9.03E-19	2.13E-21	1.97E-21	1.97E-21
TVETBO vs. GWO	5.23E-21	1.97E-21	1.97E-21	1.97E-21
TVETBO vs. TLBO	3.69E-21	1.97E-21	1.97E-21	1.97E-21
TVETBO vs. GSA	1.6E-18	2.02E-21	1.97E-21	1.97E-21
TVETBO vs. PSO	1.54E-19	2.35E-21	1.97E-21	1.97E-21
TVETBO vs. GA	2.71E-19	1.97E-21	1.97E-21	1.97E-21

**Table 7:** Optimization results of the CEC 2011 test suite.

		TVETBO	WSO	AVOA	RSA	MPA	TSA	WOA	MVO	GWO	TLBO	GSA	PSO	GA
C11-F1	mean	5.92E+00	1.84E+01	1.34E+01	2.29E+01	7.66E+00	1.91E+01	1.37E+01	1.45E+01	1.11E+01	1.92E+01	2.26E+01	1.86E+01	2.44E+01
	best	2.00E-10	1.62E+01	9.34E+00	2.12E+01	3.92E-01	1.82E+01	8.67E+00	1.17E+01	1.18E+00	1.73E+01	2.07E+01	1.10E+01	2.35E+01
	worst	1.23E+01	2.11E+01	1.72E+01	2.52E+01	1.27E+01	2.04E+01	1.77E+01	1.69E+01	1.83E+01	2.10E+01	2.40E+01	2.50E+01	2.63E+01
	std	7.20E+00	2.48E+00	4.56E+00	2.00E+00	5.91E+00	1.05E+00	4.32E+00	2.53E+00	7.60E+00	1.62E+00	1.47E+00	6.96E+00	1.42E+00
	median	5.69E+00	1.81E+01	1.34E+01	2.25E+01	8.77E+00	1.90E+01	1.42E+01	1.46E+01	1.25E+01	1.91E+01	2.29E+01	1.93E+01	2.38E+01
	rank	1	7	4	12	2	9	5	6	3	10	11	8	13
C11-F2	mean	−2.63E+01	−1.38E+01	−2.08E+01	−1.09E+01	−2.50E+01	−1.06E+01	−1.83E+01	−8.01E+00	−2.25E+01	−1.02E+01	−1.51E+01	−2.25E+01	−1.23E+01
	best	−2.71E+01	−1.53E+01	−2.13E+01	−1.14E+01	−2.57E+01	−1.45E+01	−2.19E+01	−1.01E+01	−2.47E+01	−1.14E+01	−2.03E+01	−2.39E+01	−1.48E+01
	worst	−2.54E+01	−1.26E+01	−2.00E+01	−1.04E+01	−2.37E+01	−8.30E+00	−1.41E+01	−6.41E+00	−1.87E+01	−9.13E+00	−1.08E+01	−2.00E+01	−1.05E+01
	std	7.39E-01	1.45E+00	6.13E-01	5.16E-01	9.87E-01	3.10E+00	4.22E+00	1.68E+00	2.76E+00	9.99E-01	4.56E+00	1.81E+00	2.09E+00
	median	−2.64E+01	−1.38E+01	−2.09E+01	−1.09E+01	−2.54E+01	−9.79E+00	−1.86E+01	−7.75E+00	−2.32E+01	−1.01E+01	−1.45E+01	−2.31E+01	−1.20E+01
	rank	1	8	5	10	2	11	6	13	4	12	7	3	9
C11-F3	mean	1.15E-05	1.15E-05	1.15E-05	1.15E-05	1.15E-05	1.15E-05	1.15E-05	1.15E-05	1.15E-05	1.15E-05	1.15E-05	1.15E-05	1.15E-05
	best	1.15E-05	1.15E-05	1.15E-05	1.15E-05	1.15E-05	1.15E-05	1.15E-05	1.15E-05	1.15E-05	1.15E-05	1.15E-05	1.15E-05	1.15E-05
	worst	1.15E-05	1.15E-05	1.15E-05	1.15E-05	1.15E-05	1.15E-05	1.15E-05	1.15E-05	1.15E-05	1.15E-05	1.15E-05	1.15E-05	1.15E-05
	std	2.00E-19	2.29E-11	2.63E-09	5.15E-11	1.28E-15	2.46E-14	6.39E-19	1.03E-12	3.85E-15	8.09E-14	2.07E-19	6.02E-20	2.85E-18
	median	1.15E-05	1.15E-05	1.15E-05	1.15E-05	1.15E-05	1.15E-05	1.15E-05	1.15E-05	1.15E-05	1.15E-05	1.15E-05	1.15E-05	1.15E-05
	rank	1	11	13	12	6	8	4	10	7	9	3	2	5
C11-F4	mean	0.00E+00	0.00E+00	0.00E+00	0.00E+00	0.00E+00	0.00E+00	0.00E+00	0.00E+00	0.00E+00	0.00E+00	0.00E+00	0.00E+00	0.00E+00
	best	0.00E+00	0.00E+00	0.00E+00	0.00E+00	0.00E+00	0.00E+00	0.00E+00	0.00E+00	0.00E+00	0.00E+00	0.00E+00	0.00E+00	0.00E+00
	worst	0.00E+00	0.00E+00	0.00E+00	0.00E+00	0.00E+00	0.00E+00	0.00E+00	0.00E+00	0.00E+00	0.00E+00	0.00E+00	0.00E+00	0.00E+00
	std	0.00E+00	0.00E+00	0.00E+00	0.00E+00	0.00E+00	0.00E+00	0.00E+00	0.00E+00	0.00E+00	0.00E+00	0.00E+00	0.00E+00	0.00E+00
	median	0.00E+00	0.00E+00	0.00E+00	0.00E+00	0.00E+00	0.00E+00	0.00E+00	0.00E+00	0.00E+00	0.00E+00	0.00E+00	0.00E+00	0.00E+00
	rank	1	1	1	1	1	1	1	1	1	1	1	1	1
C11-F5	mean	−3.41E+01	−2.45E+01	−2.79E+01	−1.94E+01	−3.32E+01	−2.69E+01	−2.74E+01	−2.67E+01	−3.15E+01	−9.88E+00	−2.71E+01	−7.63E+00	−8.53E+00
	best	−3.47E+01	−2.57E+01	−2.90E+01	−2.17E+01	−3.38E+01	−3.15E+01	−2.76E+01	−3.16E+01	−3.42E+01	−1.21E+01	−3.14E+01	−1.13E+01	−1.00E+01
	worst	−3.34E+01	−2.35E+01	−2.74E+01	−1.70E+01	−3.19E+01	−2.13E+01	−2.70E+01	−2.42E+01	−2.73E+01	−8.17E+00	−2.39E+01	−5.83E+00	−6.77E+00
	std	5.90E-01	9.89E-01	7.80E-01	2.59E+00	9.67E-01	4.40E+00	2.76E-01	3.66E+00	3.09E+00	1.77E+00	3.49E+00	2.72E+00	1.50E+00
	median	−3.42E+01	−2.44E+01	−2.76E+01	−1.95E+01	−3.36E+01	−2.74E+01	−2.75E+01	−2.55E+01	−3.22E+01	−9.62E+00	−2.66E+01	−6.69E+00	−8.66E+00
	rank	1	9	4	10	2	7	5	8	3	11	6	13	12
C11-F6	mean	−2.41E+01	−1.37E+01	−1.88E+01	−1.26E+01	−2.26E+01	−6.93E+00	−1.98E+01	−8.98E+00	−1.95E+01	−1.49E+00	−2.18E+01	−2.39E+00	−3.33E+00
	best	−2.74E+01	−1.43E+01	−2.02E+01	−1.34E+01	−2.57E+01	−1.63E+01	−2.30E+01	−1.72E+01	−2.22E+01	−1.69E+00	−2.67E+01	−5.29E+00	−8.79E+00
	worst	−2.30E+01	−1.33E+01	−1.70E+01	−1.16E+01	−2.13E+01	−3.58E+00	−1.26E+01	−1.42E+00	−1.78E+01	−1.42E+00	−1.74E+01	−1.42E+00	−1.42E+00
	std	2.32E+00	4.57E-01	1.55E+00	8.67E-01	2.22E+00	6.58E+00	5.18E+00	9.04E+00	2.23E+00	1.43E-01	4.20E+00	2.03E+00	3.83E+00
	median	−2.30E+01	−1.35E+01	−1.91E+01	−1.28E+01	−2.16E+01	−3.92E+00	−2.18E+01	−8.63E+00	−1.89E+01	−1.42E+00	−2.15E+01	−1.42E+00	−1.56E+00
	rank	1	7	6	8	2	10	4	9	5	13	3	12	11
C11-F7	mean	8.61E-01	1.63E+00	1.30E+00	1.95E+00	9.32E-01	1.32E+00	1.77E+00	8.82E-01	1.07E+00	1.75E+00	1.09E+00	1.13E+00	1.77E+00
	best	5.82E-01	1.58E+00	1.15E+00	1.70E+00	7.63E-01	1.15E+00	1.65E+00	8.12E-01	8.08E-01	1.54E+00	8.94E-01	8.27E-01	1.37E+00
	worst	1.03E+00	1.74E+00	1.44E+00	2.14E+00	1.01E+00	1.69E+00	1.95E+00	9.59E-01	1.31E+00	1.89E+00	1.29E+00	1.38E+00	1.98E+00
	std	2.12E-01	7.90E-02	1.61E-01	1.94E-01	1.21E-01	2.63E-01	1.31E-01	7.85E-02	2.17E-01	1.57E-01	1.91E-01	3.05E-01	2.86E-01
	median	9.18E-01	1.60E+00	1.30E+00	1.99E+00	9.76E-01	1.21E+00	1.74E+00	8.78E-01	1.09E+00	1.78E+00	1.08E+00	1.16E+00	1.86E+00
	rank	1	9	7	13	3	8	12	2	4	10	5	6	11
C11-F8	mean	2.20E+02	2.87E+02	2.41E+02	3.29E+02	2.23E+02	2.59E+02	2.68E+02	2.24E+02	2.28E+02	2.24E+02	2.47E+02	4.78E+02	2.23E+02
	best	2.20E+02	2.60E+02	2.24E+02	2.87E+02	2.20E+02	2.20E+02	2.46E+02	2.20E+02	2.20E+02	2.20E+02	2.20E+02	2.49E+02	2.20E+02
	worst	2.20E+02	3.23E+02	2.59E+02	3.75E+02	2.25E+02	3.59E+02	3.15E+02	2.37E+02	2.35E+02	2.37E+02	2.96E+02	5.83E+02	2.30E+02
	std	0.00E+00	2.92E+01	1.58E+01	3.82E+01	3.07E+00	7.10E+01	3.37E+01	8.88E+00	9.22E+00	8.88E+00	3.79E+01	1.66E+02	5.42E+00
	median	2.20E+02	2.83E+02	2.41E+02	3.27E+02	2.23E+02	2.28E+02	2.55E+02	2.20E+02	2.28E+02	2.20E+02	2.37E+02	5.41E+02	2.20E+02
	rank	1	10	6	11	2	8	9	4	5	4	7	12	3
C11-F9	mean	8.79E+03	5.77E+05	3.92E+05	1.10E+06	2.06E+04	6.83E+04	3.88E+05	1.38E+05	4.43E+04	4.23E+05	8.53E+05	1.12E+06	2.01E+06
	best	5.46E+03	3.86E+05	3.47E+05	7.18E+05	1.12E+04	4.91E+04	2.15E+05	7.80E+04	1.88E+04	3.50E+05	7.30E+05	9.00E+05	1.93E+06
	worst	1.40E+04	6.64E+05	4.22E+05	1.29E+06	2.96E+04	8.69E+04	6.58E+05	2.09E+05	7.77E+04	5.43E+05	9.19E+05	1.37E+06	2.13E+06
	std	3.89E+03	1.38E+05	3.47E+04	2.73E+05	8.52E+03	1.69E+04	2.12E+05	5.67E+04	2.62E+04	8.92E+04	8.83E+04	2.66E+05	1.04E+05
	median	7.83E+03	6.30E+05	4.00E+05	1.20E+06	2.09E+04	6.87E+04	3.40E+05	1.32E+05	4.03E+04	4.00E+05	8.82E+05	1.11E+06	2.00E+06
	rank	1	9	7	11	2	4	6	5	3	8	10	12	13
C11-F10	mean	−2.15E+01	−1.37E+01	−1.68E+01	−1.20E+01	−1.89E+01	−1.41E+01	−1.26E+01	−1.45E+01	−1.38E+01	−1.09E+01	−1.29E+01	−1.10E+01	−1.07E+01
	best	−2.18E+01	−1.50E+01	−1.70E+01	−1.23E+01	−1.93E+01	−1.88E+01	−1.33E+01	−2.12E+01	−1.43E+01	−1.10E+01	−1.34E+01	−1.11E+01	−1.08E+01
	worst	−2.08E+01	−1.31E+01	−1.64E+01	−1.17E+01	−1.86E+01	−1.17E+01	−1.21E+01	−1.11E+01	−1.26E+01	−1.09E+01	−1.20E+01	−1.10E+01	−1.07E+01
	std	4.99E-01	9.00E-01	2.70E-01	3.04E-01	4.23E-01	3.35E+00	5.42E-01	4.77E+00	8.54E-01	7.62E-02	6.82E-01	3.28E-02	4.23E-02
	median	-2.17E+01	−1.34E+01	−1.68E+01	−1.19E+01	−1.89E+01	−1.31E+01	−1.25E+01	−1.28E+01	−1.42E+01	−1.09E+01	−1.30E+01	−1.10E+01	−1.07E+01
	rank	1	7	3	10	2	5	9	4	6	12	8	11	13
C11-F11	mean	5.72E+05	5.99E+06	1.01E+06	9.15E+06	1.70E+06	6.14E+06	1.24E+06	1.33E+06	3.95E+06	5.37E+06	1.44E+06	5.39E+06	6.32E+06
	best	2.61E+05	5.71E+06	7.90E+05	8.86E+06	1.58E+06	5.11E+06	1.13E+06	6.10E+05	3.75E+06	5.35E+06	1.29E+06	5.37E+06	6.29E+06
	worst	8.29E+05	6.36E+06	1.18E+06	9.34E+06	1.83E+06	7.42E+06	1.40E+06	2.81E+06	4.33E+06	5.39E+06	1.62E+06	5.40E+06	6.40E+06
	std	2.61E+05	3.16E+05	1.80E+05	2.18E+05	1.23E+05	1.00E+06	1.20E+05	1.05E+06	2.74E+05	1.61E+04	1.42E+05	1.62E+04	5.50E+04
	median	5.99E+05	5.94E+06	1.02E+06	9.21E+06	1.69E+06	6.01E+06	1.21E+06	9.58E+05	3.86E+06	5.38E+06	1.43E+06	5.38E+06	6.30E+06
	rank	1	10	2	13	6	11	3	4	7	8	5	9	12
C11-F12	mean	1.20E+06	8.64E+06	3.45E+06	1.37E+07	1.28E+06	5.16E+06	5.98E+06	1.33E+06	1.43E+06	1.48E+07	5.95E+06	2.35E+06	1.50E+07
	best	1.16E+06	8.29E+06	3.35E+06	1.27E+07	1.20E+06	4.88E+06	5.54E+06	1.17E+06	1.26E+06	1.39E+07	5.65E+06	2.18E+06	1.48E+07
	worst	1.25E+06	8.96E+06	3.52E+06	1.45E+07	1.36E+06	5.31E+06	6.19E+06	1.48E+06	1.57E+06	1.55E+07	6.17E+06	2.57E+06	1.51E+07
	std	4.72E+04	2.95E+05	7.96E+04	7.89E+05	7.23E+04	2.10E+05	3.15E+05	1.32E+05	1.35E+05	6.83E+05	2.34E+05	1.71E+05	1.14E+05
	median	1.20E+06	8.66E+06	3.47E+06	1.37E+07	1.28E+06	5.23E+06	6.09E+06	1.34E+06	1.45E+06	1.49E+07	5.99E+06	2.33E+06	1.50E+07
	rank	1	10	6	11	2	7	9	3	4	12	8	5	13
C11-F13	mean	1.54E+04	1.59E+04	1.54E+04	1.63E+04	1.55E+04	1.55E+04	1.55E+04	1.55E+04	1.55E+04	1.60E+04	1.32E+05	1.55E+04	3.05E+04
	best	1.54E+04	1.57E+04	1.54E+04	1.59E+04	1.55E+04	1.55E+04	1.55E+04	1.55E+04	1.55E+04	1.56E+04	9.56E+04	1.55E+04	1.55E+04
	worst	1.54E+04	1.63E+04	1.54E+04	1.74E+04	1.55E+04	1.55E+04	1.56E+04	1.56E+04	1.55E+04	1.65E+04	1.82E+05	1.55E+04	7.54E+04
	std	9.09E-03	3.29E+02	9.65E-01	7.57E+02	3.04E+00	1.21E+01	5.20E+01	2.96E+01	9.12E+00	4.28E+02	4.11E+04	2.68E+01	3.14E+04
	median	1.54E+04	1.57E+04	1.54E+04	1.60E+04	1.55E+04	1.55E+04	1.55E+04	1.55E+04	1.55E+04	1.58E+04	1.26E+05	1.55E+04	1.56E+04
	rank	1	9	2	11	3	4	8	7	6	10	13	5	12
C11-F14	mean	1.83E+04	1.16E+05	1.85E+04	2.37E+05	1.86E+04	1.96E+04	1.93E+04	1.95E+04	1.93E+04	3.21E+05	1.91E+04	1.92E+04	1.91E+04
	best	1.82E+04	8.80E+04	1.84E+04	1.74E+05	1.85E+04	1.93E+04	1.91E+04	1.94E+04	1.91E+04	3.07E+04	1.88E+04	1.90E+04	1.88E+04
	worst	1.84E+04	1.62E+05	1.86E+04	3.41E+05	1.87E+04	2.01E+04	1.94E+04	1.95E+04	1.95E+04	6.21E+05	1.93E+04	1.93E+04	1.95E+04
	std	7.16E+01	3.50E+04	1.08E+02	7.88E+04	7.34E+01	4.03E+02	1.36E+02	8.32E+01	1.59E+02	2.98E+05	2.35E+02	1.37E+02	2.61E+02
	median	1.83E+04	1.07E+05	1.85E+04	2.16E+05	1.86E+04	1.94E+04	1.93E+04	1.95E+04	1.92E+04	3.17E+05	1.92E+04	1.92E+04	1.91E+04
	rank	1	11	2	12	3	10	7	9	8	13	4	6	5
C11-F15	mean	3.29E+04	9.40E+05	1.11E+05	1.98E+06	3.30E+04	5.54E+04	2.25E+05	3.31E+04	3.31E+04	1.60E+07	3.10E+05	3.33E+04	8.23E+06
	best	3.28E+04	3.87E+05	4.35E+04	8.29E+05	3.29E+04	3.31E+04	3.30E+04	3.30E+04	3.30E+04	3.35E+06	2.74E+05	3.33E+04	3.74E+06
	worst	3.30E+04	2.37E+06	1.86E+05	5.18E+06	3.30E+04	1.22E+05	3.23E+05	3.32E+04	3.31E+04	2.38E+07	3.34E+05	3.33E+04	1.41E+07
	std	7.69E+01	1.00E+06	8.03E+04	2.24E+06	6.37E+01	4.67E+04	1.38E+05	6.70E+01	4.89E+01	9.79E+06	2.94E+04	8.08E+00	4.99E+06
	median	3.29E+04	5.04E+05	1.07E+05	9.63E+05	3.30E+04	3.32E+04	2.73E+05	3.31E+04	3.31E+04	1.84E+07	3.15E+05	3.33E+04	7.53E+06
	rank	1	10	7	11	2	6	8	4	3	13	9	5	12
C11-F16	mean	1.34E+05	9.75E+05	1.35E+05	2.02E+06	1.38E+05	1.46E+05	1.42E+05	1.42E+05	1.46E+05	9.22E+07	1.94E+07	8.25E+07	7.92E+07
	best	1.31E+05	2.95E+05	1.34E+05	4.86E+05	1.36E+05	1.43E+05	1.36E+05	1.33E+05	1.44E+05	8.98E+07	9.85E+06	6.82E+07	6.40E+07
	worst	1.36E+05	2.31E+06	1.36E+05	5.02E+06	1.42E+05	1.48E+05	1.48E+05	1.51E+05	1.52E+05	9.48E+07	3.51E+07	9.86E+07	1.01E+08
	std	2.39E+03	9.53E+05	1.07E+03	2.14E+06	2.72E+03	2.48E+03	5.05E+03	8.02E+03	4.00E+03	2.21E+06	1.15E+07	1.37E+07	1.67E+07
	median	1.33E+05	6.48E+05	1.36E+05	1.28E+06	1.37E+05	1.46E+05	1.43E+05	1.42E+05	1.45E+05	9.20E+07	1.63E+07	8.16E+07	7.58E+07
	rank	1	8	2	9	3	6	5	4	7	13	10	12	11
C11-F17	mean	1.93E+06	9.29E+09	2.40E+09	1.61E+10	2.30E+06	1.33E+09	1.01E+10	3.16E+06	3.06E+06	2.31E+10	1.16E+10	2.16E+10	2.27E+10
	best	1.92E+06	7.92E+09	2.18E+09	1.16E+10	1.96E+06	1.10E+09	7.17E+09	2.31E+06	2.04E+06	2.23E+10	1.02E+10	1.91E+10	2.12E+10
	worst	1.94E+06	1.03E+10	2.63E+09	1.97E+10	2.94E+06	1.52E+09	1.34E+10	3.81E+06	4.99E+06	2.42E+10	1.23E+10	2.50E+10	2.56E+10
	std	1.20E+04	1.11E+09	2.07E+08	3.66E+09	4.64E+05	2.29E+08	2.74E+09	7.28E+05	1.40E+06	8.20E+08	9.96E+08	2.80E+09	2.11E+09
	median	1.92E+06	9.47E+09	2.40E+09	1.66E+10	2.16E+06	1.35E+09	9.84E+09	3.25E+06	2.60E+06	2.31E+10	1.20E+10	2.12E+10	2.19E+10
	rank	1	7	6	10	2	5	8	4	3	13	9	11	12
C11-F18	mean	9.42E+05	5.70E+07	6.76E+06	1.23E+08	9.73E+05	2.09E+06	9.90E+06	9.90E+05	1.03E+06	3.21E+07	1.15E+07	1.40E+08	1.19E+08
	best	9.38E+05	3.92E+07	4.05E+06	8.48E+07	9.50E+05	1.82E+06	4.24E+06	9.65E+05	9.68E+05	2.54E+07	8.58E+06	1.17E+08	1.14E+08
	worst	9.45E+05	6.48E+07	1.16E+07	1.40E+08	1.03E+06	2.45E+06	1.74E+07	1.00E+06	1.21E+06	3.47E+07	1.45E+07	1.55E+08	1.23E+08
	std	2.77E+03	1.26E+07	3.71E+06	2.73E+07	4.24E+04	3.15E+05	5.84E+06	1.79E+04	1.23E+05	4.69E+06	2.79E+06	1.78E+07	3.75E+06
	median	9.43E+05	6.20E+07	5.69E+06	1.33E+08	9.54E+05	2.05E+06	8.98E+06	9.97E+05	9.80E+05	3.41E+07	1.15E+07	1.43E+08	1.19E+08
	rank	1	10	6	12	2	5	7	3	4	9	8	13	11
C11-F19	mean	1.03E+06	5.61E+07	6.86E+06	1.20E+08	1.14E+06	2.52E+06	1.06E+07	1.49E+06	1.38E+06	3.69E+07	6.46E+06	1.79E+08	1.19E+08
	best	9.68E+05	4.78E+07	6.26E+06	1.04E+08	1.07E+06	2.27E+06	2.10E+06	1.13E+06	1.24E+06	2.58E+07	2.43E+06	1.62E+08	1.16E+08
	worst	1.17E+06	7.13E+07	8.33E+06	1.51E+08	1.30E+06	2.98E+06	1.92E+07	2.00E+06	1.56E+06	4.60E+07	8.50E+06	2.07E+08	1.23E+08
	std	9.97E+04	1.11E+07	1.03E+06	2.32E+07	1.10E+05	3.33E+05	8.44E+06	3.80E+05	1.39E+05	9.19E+06	2.89E+06	2.03E+07	2.81E+06
	median	9.83E+05	5.26E+07	6.44E+06	1.13E+08	1.10E+06	2.41E+06	1.05E+07	1.42E+06	1.35E+06	3.78E+07	7.46E+06	1.73E+08	1.19E+08
	rank	1	10	7	12	2	5	8	4	3	9	6	13	11
C11-F20	mean	9.41E+05	5.96E+07	6.08E+06	1.30E+08	9.61E+05	1.86E+06	7.52E+06	9.74E+05	1.00E+06	3.58E+07	1.48E+07	1.65E+08	1.19E+08
	best	9.36E+05	5.25E+07	5.35E+06	1.14E+08	9.57E+05	1.67E+06	7.08E+06	9.64E+05	9.79E+05	3.50E+07	9.79E+06	1.51E+08	1.14E+08
	worst	9.47E+05	7.06E+07	6.85E+06	1.54E+08	9.63E+05	2.18E+06	8.10E+06	9.86E+05	1.02E+06	3.67E+07	2.29E+07	1.79E+08	1.24E+08
	std	5.01E+03	8.13E+06	6.53E+05	1.83E+07	2.67E+03	2.53E+05	4.58E+05	1.03E+04	1.77E+04	7.16E+05	6.01E+06	1.66E+07	4.51E+06
	median	9.41E+05	5.77E+07	6.05E+06	1.26E+08	9.62E+05	1.80E+06	7.44E+06	9.73E+05	1.00E+06	3.58E+07	1.32E+07	1.65E+08	1.20E+08
	rank	1	10	6	12	2	5	7	3	4	9	8	13	11
C11-F21	mean	1.27E+01	5.16E+01	2.20E+01	7.88E+01	1.61E+01	3.05E+01	3.98E+01	2.81E+01	2.27E+01	1.04E+02	4.17E+01	1.09E+02	1.06E+02
	best	9.97E+00	4.23E+01	2.07E+01	5.85E+01	1.39E+01	2.70E+01	3.63E+01	2.48E+01	2.09E+01	4.96E+01	3.66E+01	9.43E+01	6.04E+01
	worst	1.50E+01	6.16E+01	2.38E+01	9.92E+01	1.83E+01	3.22E+01	4.41E+01	3.12E+01	2.51E+01	1.53E+02	4.47E+01	1.22E+02	1.30E+02
	std	2.41E+00	8.74E+00	1.40E+00	1.89E+01	2.17E+00	2.48E+00	3.66E+00	3.73E+00	1.92E+00	4.47E+01	3.83E+00	1.41E+01	3.38E+01
	median	1.30E+01	5.13E+01	2.17E+01	7.88E+01	1.60E+01	3.13E+01	3.93E+01	2.82E+01	2.25E+01	1.06E+02	4.28E+01	1.10E+02	1.17E+02
	rank	1	9	3	10	2	6	7	5	4	11	8	13	12
C11-F22	mean	1.61E+01	4.80E+01	2.79E+01	6.53E+01	1.92E+01	3.27E+01	4.75E+01	3.29E+01	2.53E+01	1.06E+02	4.78E+01	1.10E+02	9.54E+01
	best	1.15E+01	4.15E+01	2.26E+01	4.70E+01	1.64E+01	2.87E+01	4.11E+01	2.52E+01	2.41E+01	6.84E+01	3.97E+01	9.23E+01	9.46E+01
	worst	1.96E+01	5.36E+01	3.32E+01	7.51E+01	2.13E+01	3.53E+01	5.23E+01	3.81E+01	2.63E+01	1.25E+02	5.72E+01	1.21E+02	9.70E+01
	std	4.20E+00	5.48E+00	5.29E+00	1.32E+01	2.48E+00	3.00E+00	5.30E+00	6.10E+00	1.08E+00	2.70E+01	7.53E+00	1.38E+01	1.13E+00
	median	1.67E+01	4.84E+01	2.78E+01	6.95E+01	1.95E+01	3.35E+01	4.82E+01	3.42E+01	2.55E+01	1.15E+02	4.72E+01	1.13E+02	9.51E+01
	rank	1	9	4	10	2	5	7	6	3	12	8	13	11
Sum rank		22	191	109	231	55	146	145	118	97	222	157	198	224
Mean rank		1.00E+00	8.68E+00	4.95E+00	1.05E+01	2.50E+00	6.64E+00	6.59E+00	5.36E+00	4.41E+00	1.01E+01	7.14E+00	9.00E+00	1.02E+01
Total rank		1	2	12	4	13	3	11	9	6	7	10	5	8
Wilcoxon: *p*-value			4.8E-12	8.49E-15	1.71E-15	0.001914	5.36E-15	5.76E-15	1.75E-11	2.11E-12	3.66E-15	8.8E-15	1.71E-15	2.5E-15

## Data Availability

Not applicable.
